# Supplementary material for: Diet-Dependent Cognitive Benefits of Exogenous Ketone Body Precursor, (R,S)-1,3,-Butanediol, in a Mouse Model of Tauopathy
Source: bioRxiv. 2026 Jun 8:2026.06.03.729999. Preprint. [Version 1] doi: 10.64898/2026.06.03.729999 (PMC13277812; doi:10.64898/2026.06.03.729999)
Supplement: Supplementary file 1 [file NIHPP2026.06.03.729999v1-supplement-1.pdf]

bioRxiv preprint doi: https://doi.org/10.64898/2026.06.03.729999; this version posted June 8, 2026. The copyright holder for this preprint (which was not certified by peer review) is the author/funder, who has granted bioRxiv a license to display the preprint in perpetuity. It is made available under a CC-BY 4.0 International license.

Supplemental Methods:

Enantioseparation and Quantification of D/L-βOHB: Extracted serum samples were dried in a speed-vac and derivatization was adapted from previous reference[20,21]. Briefly, freshly prepared 5 mM (S)-(+)-1-(2-Pyrrolidinylmethyl)-pyrrolidine (PMP), 10 mM triphenylphosphine (TPP), and 10 mM 2,2′- dipyridyl disulfide (DPS) were dissolved separately in ACN and equal volumes were added to the dried samples, vortexed, and incubated for 90 min at room temperature. Samples were dried again in a speedvac and then resuspended in 40 µL of 2% Methanol, 98% H_2O, and 0.0125% acetic acid. Samples were vortexed, sonicated for 5 min, centrifuged, and supernatant injected into LC-MS/MS system using positive ionization mode. Standards of D- or L-βOHB were also dried, derivatized, and injected alongside samples to confirm enantiomeric identity.

Barnes Maze Testing: Cognitive function was assessed using a Barnes Maze protocol as previously described[22,23]. The Barnes Maze test assesses spatial learning and memory in rodents by placing mice on an elevated, circular platform (36-inch high, 36-inch diameter) with 20 x 2-inch holes. One of the holes leads to an escape box. We used a maze with a tan background since rTg4510 mice present with white, agouti, or black fur. Testing occurred in a brightly lit room with a 300-lux overhead light to motivate mice to find the dark escape box. Four contextual markers were positioned around the maze. Mice were first habituated to the maze platform and guided to the escape box. For each trial, a mouse started at the center of the platform. Between trials, the maze was cleaned with 70% ethanol to minimize scent trails. Then, from days 1 to 4, mice completed four acquisition trials daily, with a 15-minute inter-trial interval. Trials ended when the mouse found the escape box or after 3 minutes, whichever occurred sooner. Primary latency to escape hole entry was recorded for each trial. If the mouse did not find the escape hole within the 3-minute trial, the mouse was gently guided to the escape box and allowed 30 seconds rest inside the escape box. On day 5, mice were subjected to a 90-second probe trial with the escape box removed. We recorded primary errors, primary latency to escape hole entry, and percentage of

bioRxiv preprint doi: https://doi.org/10.64898/2026.06.03.729999; this version posted June 8, 2026. The copyright holder for this preprint (which was not certified by peer review) is the author/funder, who has granted bioRxiv a license to display the preprint in perpetuity. It is made available under a CC-BY 4.0 International license.

time spent in the goal quadrant derived from video acquisition using AnyMaze Software (Stoelting Co.; Wood Dale, IL) provided by the UMN Behavioral Phenotyping Core.

Isotope Tracing Untargeted Metabolomics (ITUM): Mice were fasted for 18h prior to isotope tracing experiments. Fifteen minutes after an intraperitoneal injection of 5 μmol/g BW [U-$^{13}C_6$]glucose, mice were euthanized with pentobarbital and decapitated for rapid collection of the brain frontal cortex, which was immediately freeze-clamped. Blood samples were collected at t=0 min and t=15 min to quantify plasma glucose concentration. ITUM workflow proceeded as previously described[26]. Briefly, 20 mg brain samples were homogenized in (2:2:1) Methanol:ACN:H_2O extraction buffer, and then metabolites extracted from 2 mg tissue homogenate in 1 mL extraction buffer by three cycles of vortexing, freeze-thawing, and water bath sonication. Samples were incubated at -20℃ for 1 hour, followed by 10 min centrifugation. Exactly 800 μL supernatant was transferred to new tubes, and the solvent was evaporated in a speed-vac. Finally, the dried metabolite pellet was reconstituted in 100 μL (1:1) ACN:H_2O with the aid of vortexing and sonication, then incubated at 4℃ for 1 h before spinning samples and loading in vials for LC/MS analysis. To aid metabolite identification, an in-house standard mixture of 100 metabolites and a pooled sample mix were analyzed by data-dependent acquisition (DDA). Identifications were based on retention time compared to authentic standard, m/z that matches theoretical m/z predicted from molecular formula (±10 ppm), and MS2 fragmentation patter matched to online databases (e.g. mzCloud). Electrospray ionization drift was monitored by injecting a pooled quality control (QC) sample at regular intervals.

Extracted metabolites were separated using the Thermo Fisher Scientific Vanquish UHPLC system, followed by detection using high mass resolution mass spectrometry on the Thermo Fisher Scientific QExactive plus hybrid quadrupole-orbitrap mass spectrometer, fitted with heated electrospray ionization source. Atlantis Premier BEH Z-HILIC column (1.7 μm particle size, 2.1 mm x 100 mm) was used to separate metabolites using hydrophilic interaction liquid

bioRxiv preprint doi: https://doi.org/10.64898/2026.06.03.729999; this version posted June 8, 2026. The copyright holder for this preprint (which was not certified by peer review) is the author/funder, who has granted bioRxiv a license to display the preprint in perpetuity. It is made available under a CC-BY 4.0 International license.

chromatography (HILIC) mode using following mobile phases: MPA = 15 mM NH_4HCO_3 in 100% H_2O, pH = 9.0; MPB = 15 mM NH_4HCO_3 in 90% ACN, pH = 9.0. Total run time was 10 min, flow rate was 0.5 mL/min for the first 6 minutes and then increased to 1 mL/min for the remainder of the run. Column chamber was maintained at 30 °C. Mobile phase gradient was as follows: 0 to 0.1 min, 90% MPB; 0.1 to 5 min, 90 → 65% MPB; 5 to 6 min, 65% MPB; 6 to 6.5 min, 65 → 90% MPB; 6.5 to 10 min, 90% MPB. For analyses, both blanks and QC samples were injected periodically throughout the run. Blank samples containing only ACN:H_2O (1:1)[26]. Thermo Compound Discoverer 3.3 was used for the analysis. To confirm fractional enrichment of metabolites with low enrichment (<1%), manual integration using QuanBrowser on Thermo Fisher Xcalibur software was performed.

Shotgun lipidomics: Lipidomics extractions and analyses were performed as previously described[27]. Briefly, brains were freeze-clamped, weighed (~20 mg) and homogenized using zirconium oxide beads and an automated tissue homogenizer (Omni International, Bead Ruptor 12, 19-050A) using 1.0 mL ice-cold, 0.1x PBS lacking both $MgCl_2$ and $CaCl_2$. Total protein was determined using BCA assay, then internal standards were added based on the sample protein amount. Bulk lipid extraction was completed using a modified Bligh-Dyer method, as described previously[28]. Finally, samples were directly infused into a TSQ Vantage triple quadrupole mass spectrometer (Thermo Fisher Scientific, San Jose, CA) equipped with an automated Triversa Nanomate nanospray device (Advion Biosciences, Ithaca, NY) for multidimensional MS-based shotgun lipidomics.

bioRxiv preprint doi: https://doi.org/10.64898/2026.06.03.729999; this version posted June 8, 2026. The copyright holder for this preprint (which was not certified by peer review) is the author/funder, who has granted bioRxiv a license to display the preprint in perpetuity. It is made available under a CC-BY 4.0 International license.

Supplemental Figure 1

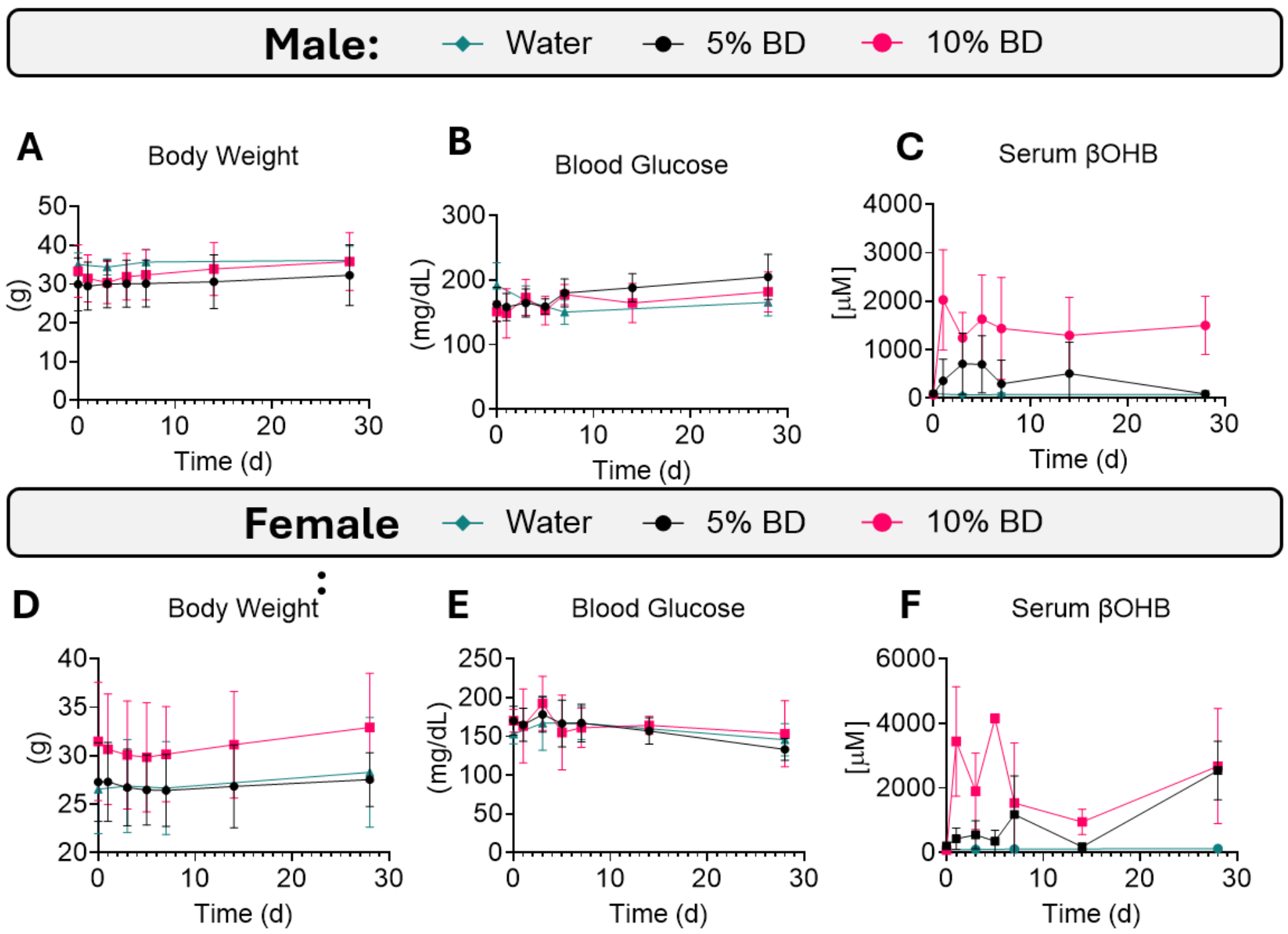

Supplemental Figure 1. Kinetics of 1,3-butanediol (BD) in male and female mice. Male and female mice were treated acutely with 5% or 10% BD in drinking water for 28 days. In male mice given the BD treatment, water controls showed no effects on body weight (**A**), blood glucose (**B**), or serum βOHB (**C**). Similarly, in female mice given the BD treatment, water controls showed no effects on body weight (**D**), blood glucose (**E**), or serum βOHB (**F**) compared to baseline measures. Data for this figure were re-plotted from figure 1 to show comparison to water controls (*n*=4-5 water controls per panel). BD = 1,3-butanediol, βOHB= β-hydroxybutyrate.

bioRxiv preprint doi: https://doi.org/10.64898/2026.06.03.729999; this version posted June 8, 2026. The copyright holder for this preprint (which was not certified by peer review) is the author/funder, who has granted bioRxiv a license to display the preprint in perpetuity. It is made available under a CC-BY 4.0 International license.

Supplemental Figure 2

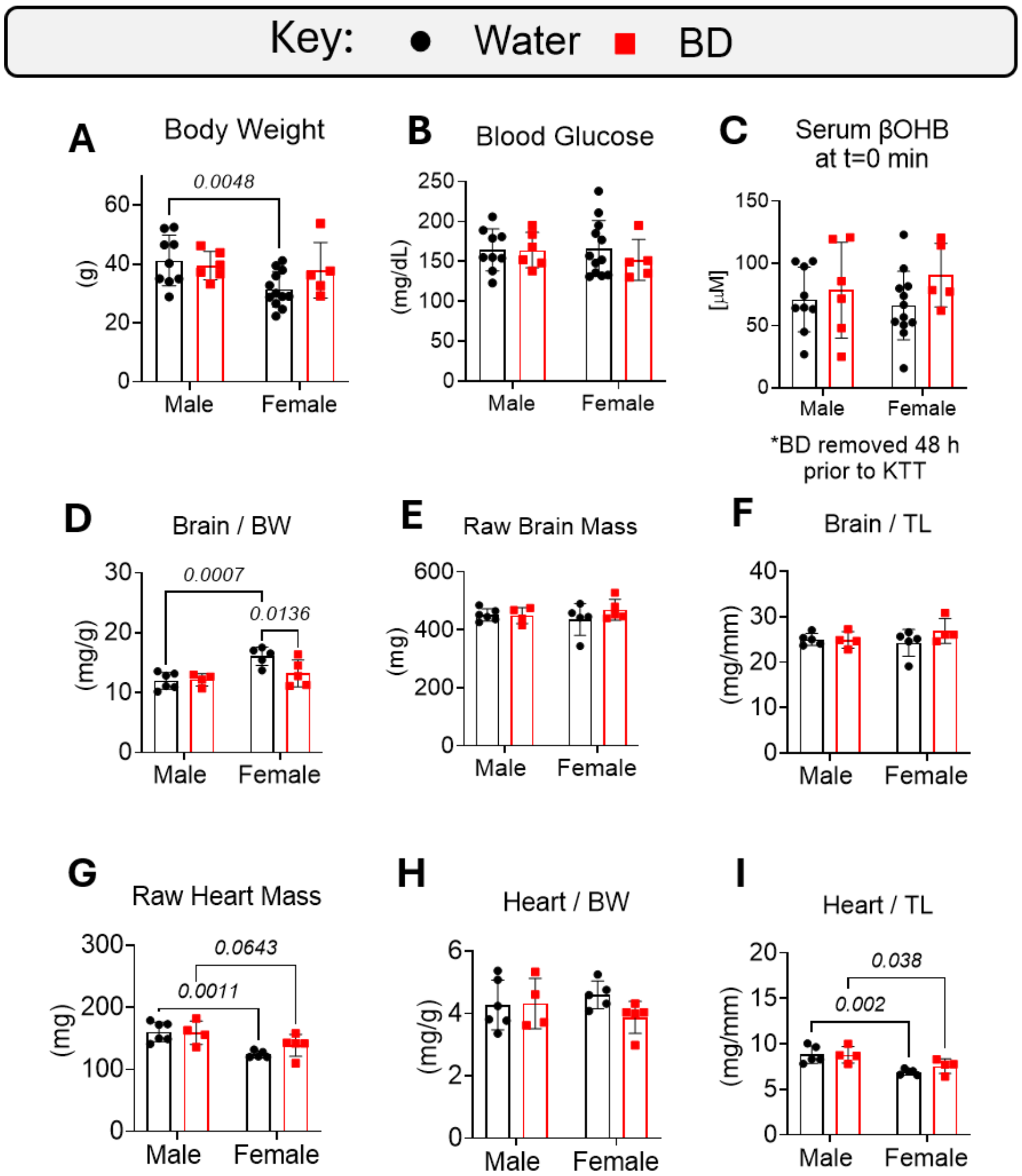

Supplemental Figure 2. Chronic BD treatment has minimal influence on body weight and tissue mass in healthy mice. Male and female mice were treated with 10% BD in drinking water for 20 weeks and then subjected to a ketone tolerance test (KTT). Effects of treatment on body weight (**A**) and non-fasted blood glucose (**B**) in BD-treated mice compared to normal water controls. After 48 h removal of BD water, serum βOHB was unchanged between groups (**C**). BD treatment had no effect on raw or normalized brain mass (**D-F**), and there were no effects of BD treatment on raw or normalized heart (**G-I**). Data are expressed as mean ± SD, *n*=4-5/group. P-values indicated where p<0.10 following two-way ANOVA and Tukey's post-hoc test. BD = 1,3-butanediol, βOHB= β-hydroxybutyrate, TL = tibia length.

bioRxiv preprint doi: https://doi.org/10.64898/2026.06.03.729999; this version posted June 8, 2026. The copyright holder for this preprint (which was not certified by peer review) is the author/funder, who has granted bioRxiv a license to display the preprint in perpetuity. It is made available under a CC-BY 4.0 International license.

Supplemental Figure 3

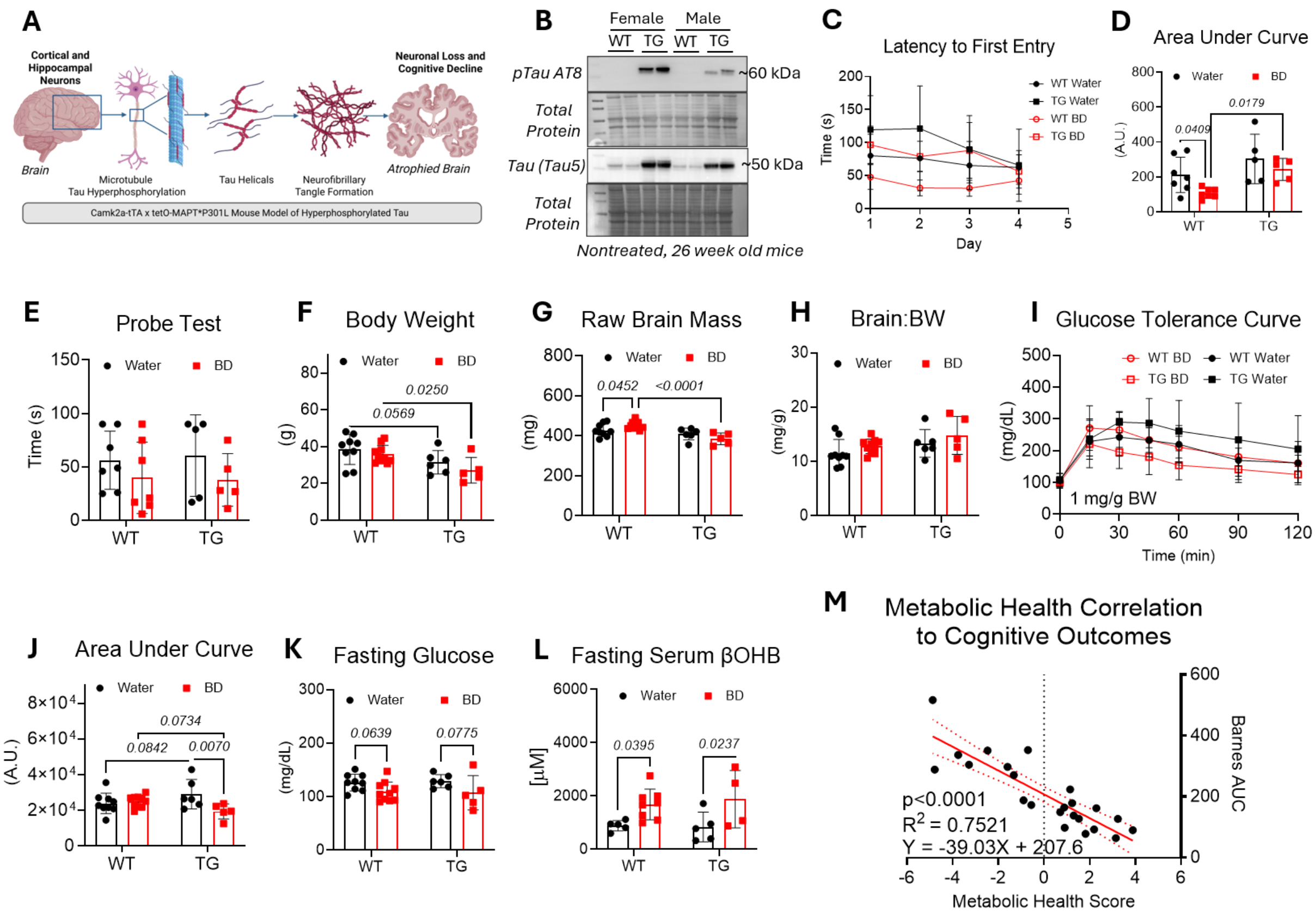

Supplemental Figure 3. Twenty-week BD treatment may improve cognitive outcomes in non-tauopathy, but not tauopathy, male mice. Schematic of tauopathy mouse model used in this study (**A**) with representative immunoblot of phosphorylated human Tau (AT8) and total Tau (Tau5) in nontreated, 26-week aged male and female tauopathy mice and non-transgenic control mice (**B**, *n*=2/group). Male tauopathy mice and non-transgenic littermate controls were also provided with 10% BD in drinking water or normal drinking water for 20 weeks, and cognitive outcomes were assessed. Mice were subjected to Barnes Maze behavioral testing following 20-week treatment. Latency to escape hole entry during memory acquisition phase, days 1-4 (**C**, *n*=5-7/group) and corresponding area under the curve for the trials (**D**). Memory recall probe test, day 5 (**E**, *n*=5-7/group) in male mice. Effects of genotype and 20 weeks BD treatment on body weight (**F**), raw brain mass (**G**), and brain mass normalized to body weight (**H**). Systemic glucose tolerance test (**I**) performed at 25 weeks of age with corresponding area under the curve (**J**) for the 120-min test. Following the 20-week intervention, mice were fasted for 18 h and then euthanized. Effects of 20-week BD treatment on fasting blood glucose (**K**) and serum βOHB while consuming BD-treated water (**L**). Pearson's correlations were calculated for variables, and a metabolic health score (MHS) was constructed to predict cognitive outcomes in male mice, as shown in (**M**). Data are presented as mean ± SD, *n*=5-10/group unless otherwise indicated. P-values indicated when $p<0.10$ following two-way ANOVA and Tukey's post-hoc test. BD = 1,3-butanediol, βOHB= β-hydroxybutyrate, ITUM = isotope tracing untargeted metabolomics, A.U. =

bioRxiv preprint doi: https://doi.org/10.64898/2026.06.03.729999; this version posted June 8, 2026. The copyright holder for this preprint (which was not certified by peer review) is the author/funder, who has granted bioRxiv a license to display the preprint in perpetuity. It is made available under a CC-BY 4.0 International license.

arbitrary units, BW = body weight, WT = non-tauopathy control mice, TG = tauopathy mice, MHS = metabolic health score

bioRxiv preprint doi: https://doi.org/10.64898/2026.06.03.729999; this version posted June 8, 2026. The copyright holder for this preprint (which was not certified by peer review) is the author/funder, who has granted bioRxiv a license to display the preprint in perpetuity. It is made available under a CC-BY 4.0 International license.

Supplemental Figure 4

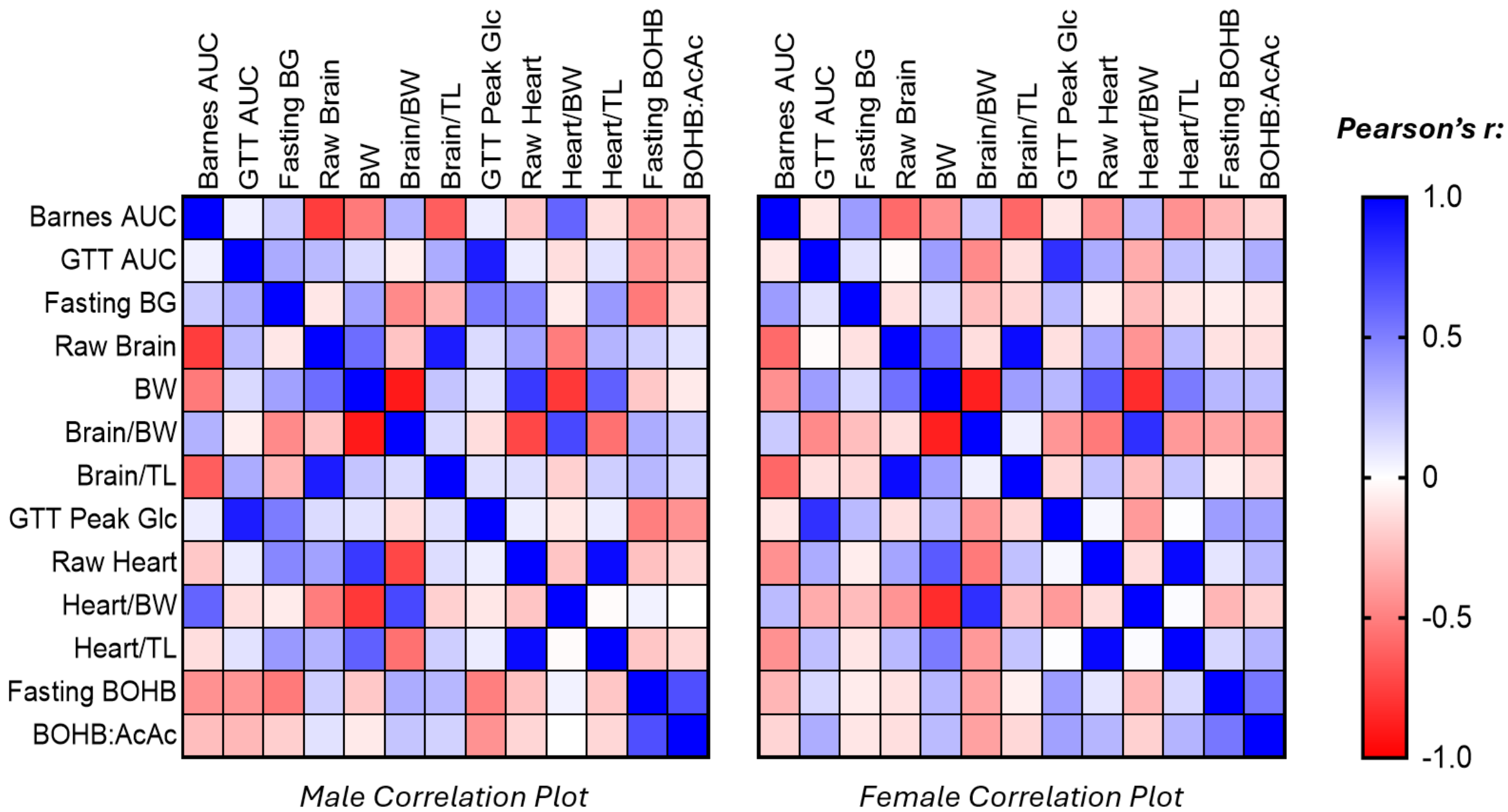

Supplemental Figure 4. Correlation heatmaps for male and female mice consuming normal chow with or without BD for 20 weeks. Heatmaps showing correlations among variables in male and female mice, with or without 10% BD in the drinking water for 20-week treatment. Data represented as Pearson's r, *n*=23-29/group. AUC = area under curve, βOHB= β-hydroxybutyrate, GTT = glucose tolerance test, BG = blood glucose, BW = body weight, Glc = glucose, TL = tibia length for normalization.

bioRxiv preprint doi: https://doi.org/10.64898/2026.06.03.729999; this version posted June 8, 2026. The copyright holder for this preprint (which was not certified by peer review) is the author/funder, who has granted bioRxiv a license to display the preprint in perpetuity. It is made available under a CC-BY 4.0 International license.

Supplemental Figure 5

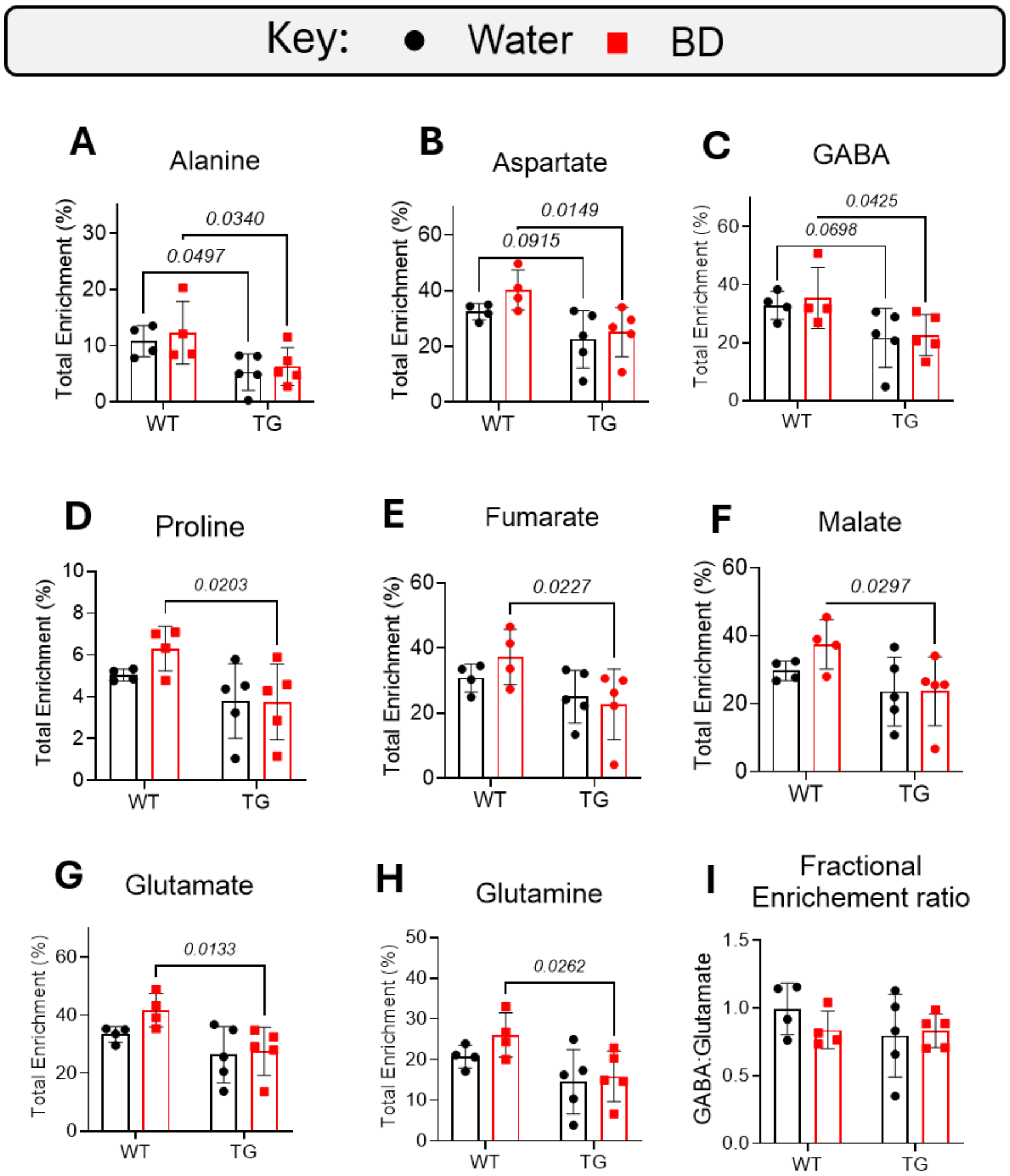

Supplemental Figure 5. Twenty-week BD treatment influences acute glucose utilization into anabolic pathways in the forebrain of male mice. Following 20-week BD treatment, male mice underwent 15-min ITUM with [U-$^{13}C_6$]-glucose delivered via I.P. injection. Total ^{13}C enrichment from glucose-derived carbon was measured in metabolites from freeze-clamped, forebrain sections of male mice. Fractional enrichment of ^{13}C was traced in Alanine (**A**), Aspartate (**B**), GABA (**C**), Proline (**D**), Fumarate (**E**), Malate (**F**), Glutamate (**G**), and Glutamine (**H**) in forebrain regions. Ratio of glucose-derived enrichment in GABA: Glutamate shown in (**I**). Data are expressed as mean ± SD, *n*=4-5/g. P-values indicated when $p<0.10$ following two-way ANOVA and Tukey's post-hoc test. GABA = gamma-aminobutyric acid, WT = non-tauopathy control mice, TG = tauopathy mice

bioRxiv preprint doi: https://doi.org/10.64898/2026.06.03.729999; this version posted June 8, 2026. The copyright holder for this preprint (which was not certified by peer review) is the author/funder, who has granted bioRxiv a license to display the preprint in perpetuity. It is made available under a CC-BY 4.0 International license.

Supplemental Figure 6

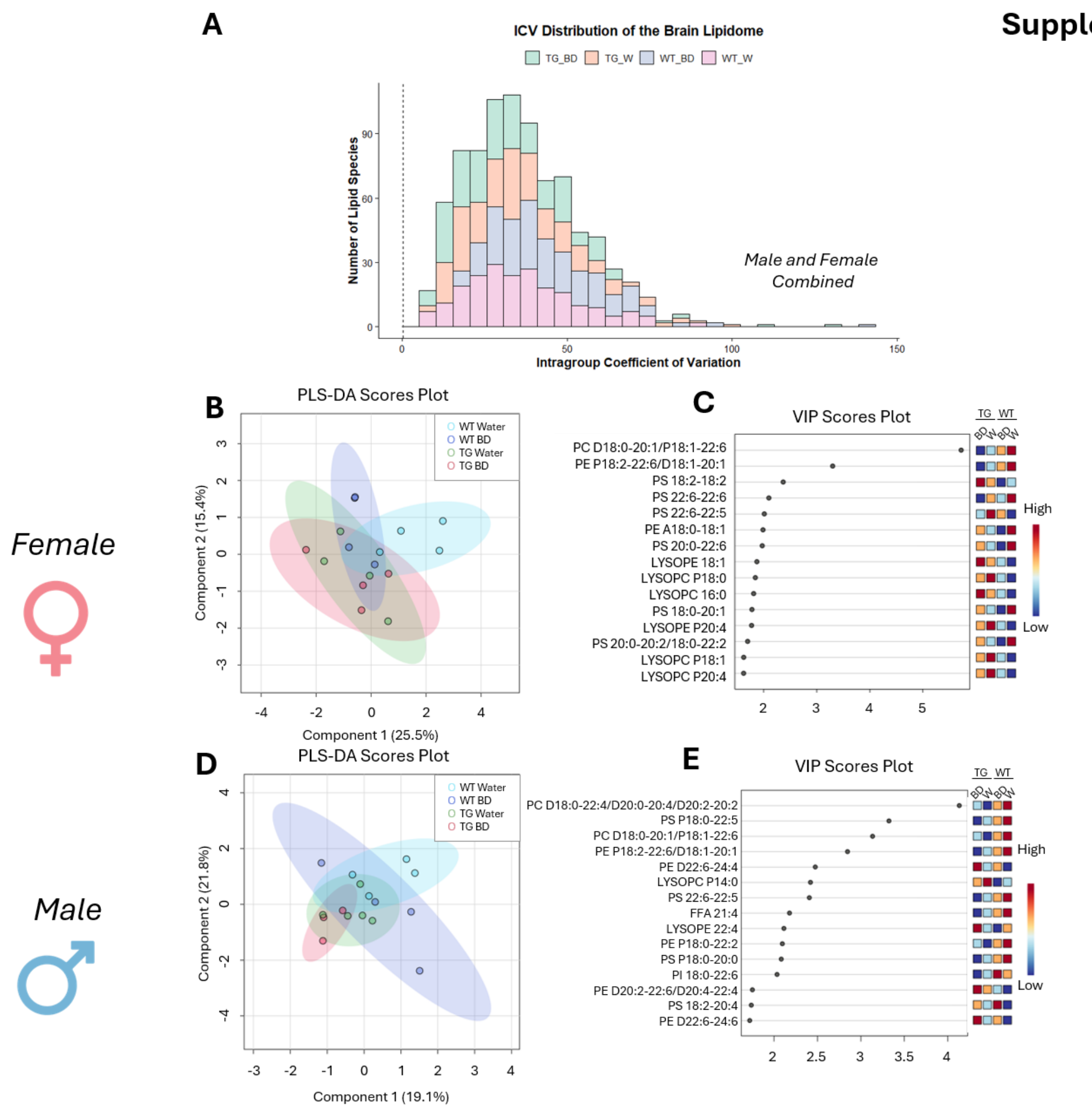

Supplemental Figure 6. BD treatment modestly influences the brain lipidome of mice in a treatment and genotype-specific manner. Following 20-week BD treatment, a subset of male and female mouse brains was freeze-clamped, and forebrain sections were subjected to lipidomic analyses. Intragroup coefficient of variation for pooled male and female samples from each treatment group is shown in (**A**). Absolute concentrations of lipid species were analyzed using Metaboanalyst 6.0 software and were log-transformed to show group separation via partial least squares-discriminant analysis in female brains (PLS-DA, **B**), highlighting the corresponding variable importance plot (VIP) scores for the top 15 metabolites (**C**). A PLS-DA plot was also constructed for male brains (**D**) with corresponding VIP scores for the top 15 metabolites (**E**). Data are expressed as *n*=3-5/group. WT = non-tauopathy control mice, TG = tauopathy mice, PC = phosphatidylcholine, PE = phosphatidylethanolamine, PA = phosphatidic acid, PI = phosphatidylinositol, PS = phosphatidylserine, SM = sphingomyelin, CBS = cerebroside, FFA = free fatty-acid, LPC = lysoPC, LPE = lysoPE, Car = acylcarnitine, W = water treatment, BD = 1,3-butanediol treatment

bioRxiv preprint doi: https://doi.org/10.64898/2026.06.03.729999; this version posted June 8, 2026. The copyright holder for this preprint (which was not certified by peer review) is the author/funder, who has granted bioRxiv a license to display the preprint in perpetuity. It is made available under a CC-BY 4.0 International license.

Supplemental Figure 7

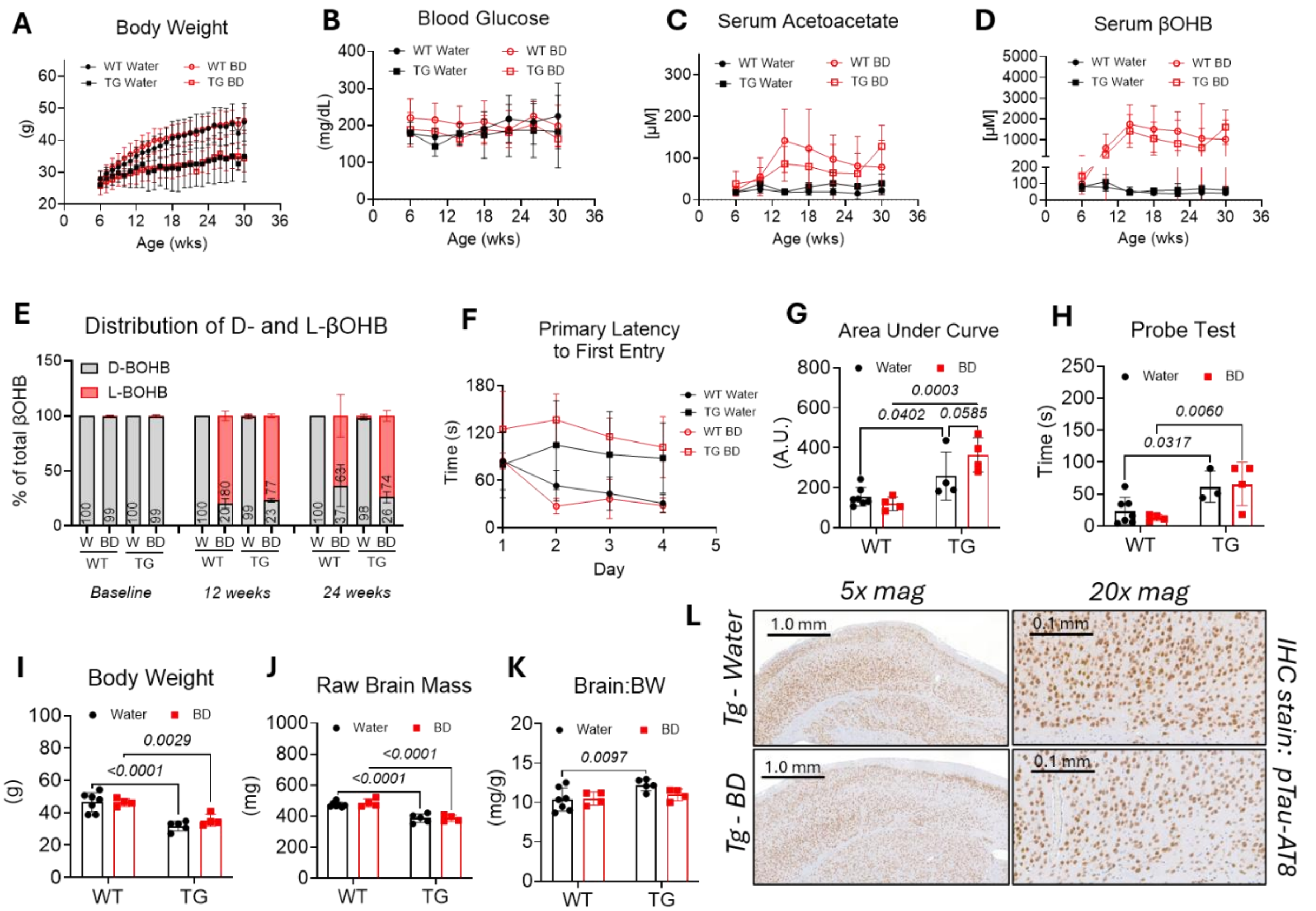

Supplemental Figure 7. Thirty-week BD treatment has no significant effects on cognitive or brain outcomes in male tauopathy-transgenic mice. Male tauopathy mice and non-transgenic littermate controls were also provided with 10% BD in drinking water or normal drinking water for 30 weeks, and cognitive outcomes were assessed. Effects of the transgene and BD treatment on body mass (**A**), non-fasted blood glucose (**B**), serum Acetoacetate (**C**), and serum βOHB (**D**) over 30-week treatment. Total percentage of D- and L-βOHB in circulation at baseline (6 weeks of age), 12-week treatment (18 weeks of age), and 24-week treatment (30 weeks of age, **E**, *n*=3/group). Mice were subjected to the Barnes-Maze for cognitive testing. Latency to escape hole entry during memory acquisition phase, days 1-4 (**F**) and corresponding area under the curve for the trials (**G**). A memory probe test was conducted on day 5 and is shown in (**H**). Thirty-week BD treatment did not influence body mass (**I**), raw brain mass (**J**), or brain mass normalized to body weight (**K**) in transgenic or non-transgenic male mice at tissue harvest. Representative images from immunohistochemistry staining of paired helical filaments in the forebrain region (**L**) from tauopathy-transgenic mice exposed to BD treatment or normal drinking water. Data are expressed as mean ± SD, *n*=4-7/group unless otherwise indicated. P-values indicated where p<0.10 following two-way ANOVA and Tukey's post-hoc test. BD = 1,3-butanediol, A.U. = arbitrary units, βOHB= β-hydroxybutyrate, mag = magnification, IHC = immunohistochemistry, WT = non-tauopathy control mice, TG = tauopathy mice

bioRxiv preprint doi: https://doi.org/10.64898/2026.06.03.729999; this version posted June 8, 2026. The copyright holder for this preprint (which was not certified by peer review) is the author/funder, who has granted bioRxiv a license to display the preprint in perpetuity. It is made available under a CC-BY 4.0 International license.

Supplemental Figure 8

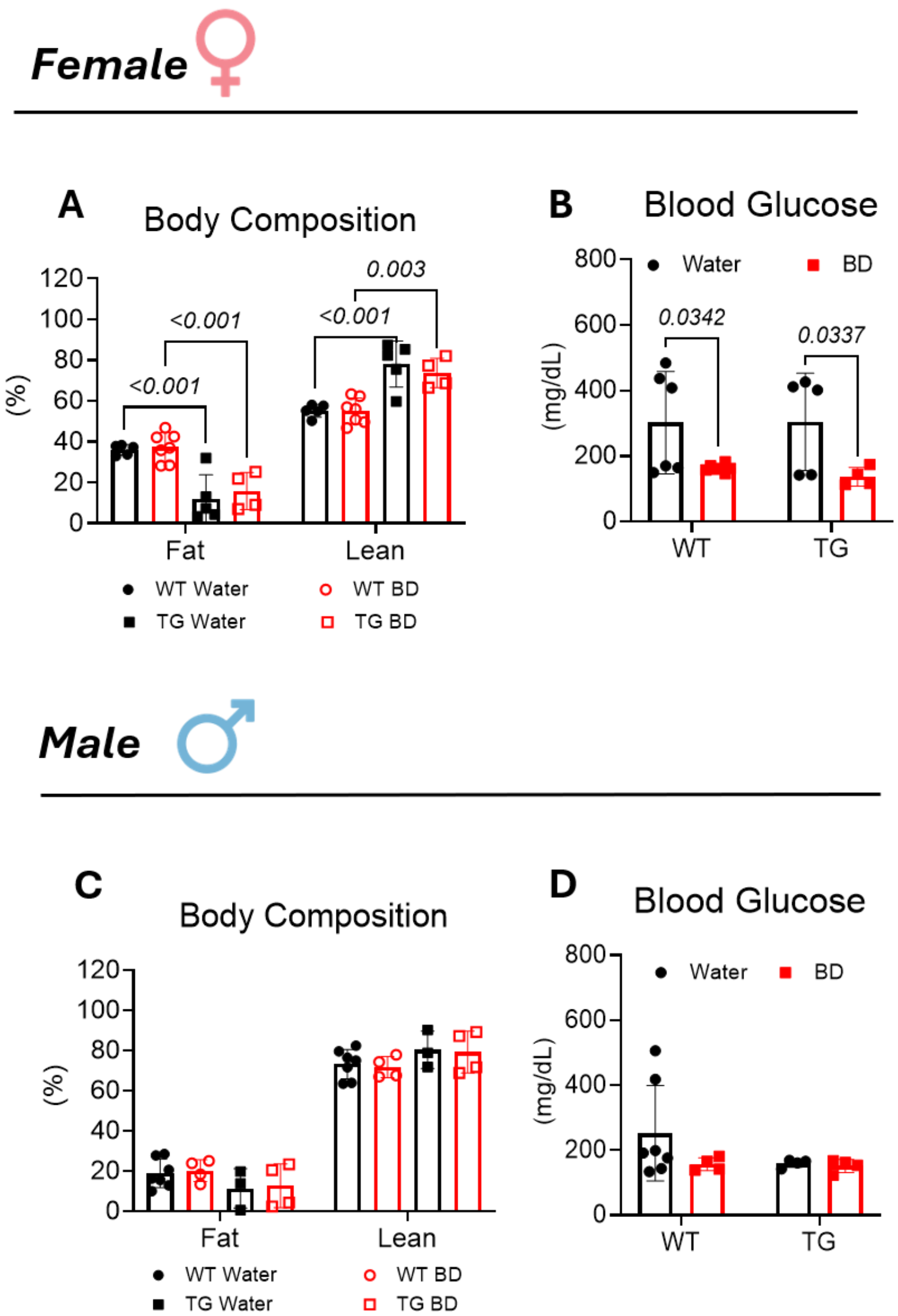

Supplemental Figure 8. Sex-dependent effects of 30-week BD treatment on body composition and blood glucose. Following 30-week BD treatment in tauopathy and non-tauopathy mice, we measured body composition in female mice (**A**) and recorded the effects of chronic BD treatment on non-fasting blood glucose (**B**). We also measured body composition in male mice (**C**) and recorded effects of chronic BD treatment on non-fasting blood glucose (**D**). Data are expressed as mean ± SD, *n*=4-7/group. P-values indicated where p<0.10 following two-way ANOVA and Tukey's post-hoc test. WT = non-tauopathy control mice, TG = tauopathy mice

bioRxiv preprint doi: https://doi.org/10.64898/2026.06.03.729999; this version posted June 8, 2026. The copyright holder for this preprint (which was not certified by peer review) is the author/funder, who has granted bioRxiv a license to display the preprint in perpetuity. It is made available under a CC-BY 4.0 International license.

Supplemental Figure 9

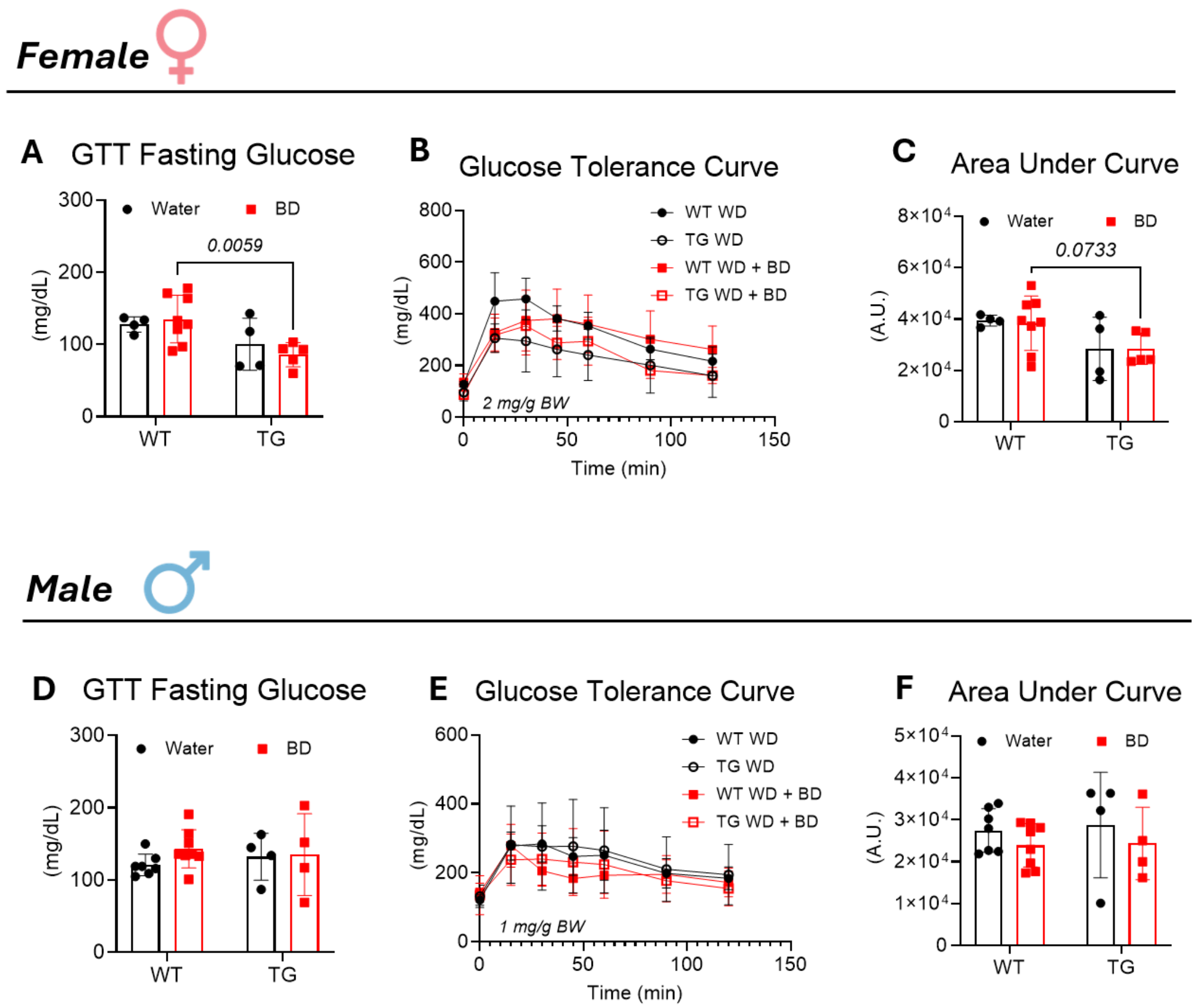

Supplemental Figure 9. BD treatment does not influence systemic glucose tolerance in mice on a high-fat, Western diet (WD). Following a 20-week high-fat, Western diet either with or without BD treatment, mice were fasted for 18 h and then subjected to a glucose tolerance test (GTT) via I.P. injection of glucose (1 mg/g BW males; 2 mg/g BW females). Effects of BD treatment on female mouse fasted blood glucose values (**A**) or glucose tolerance curve (**B**) with corresponding area under the curve (**C**) when consuming the high-fat, Western diet. Effects of the BD treatment on male mouse fasted blood glucose values (**D**) or glucose tolerance curve (**E**) with corresponding area under the curve (**F**) when mice were consuming a high-fat, Western diet. Data are expressed as mean ± SD, *n*=4-8/group. P-values indicated where $p<0.10$ following two-way ANOVA with Tukey's post-hoc test. WD = Western diet, BD = 1,3-butanediol, GTT = glucose tolerance test, WT = non-tauopathy control mice, TG = tauopathy mice

bioRxiv preprint doi: https://doi.org/10.64898/2026.06.03.729999; this version posted June 8, 2026. The copyright holder for this preprint (which was not certified by peer review) is the author/funder, who has granted bioRxiv a license to display the preprint in perpetuity. It is made available under a CC-BY 4.0 International license.

Supplemental Figure 10

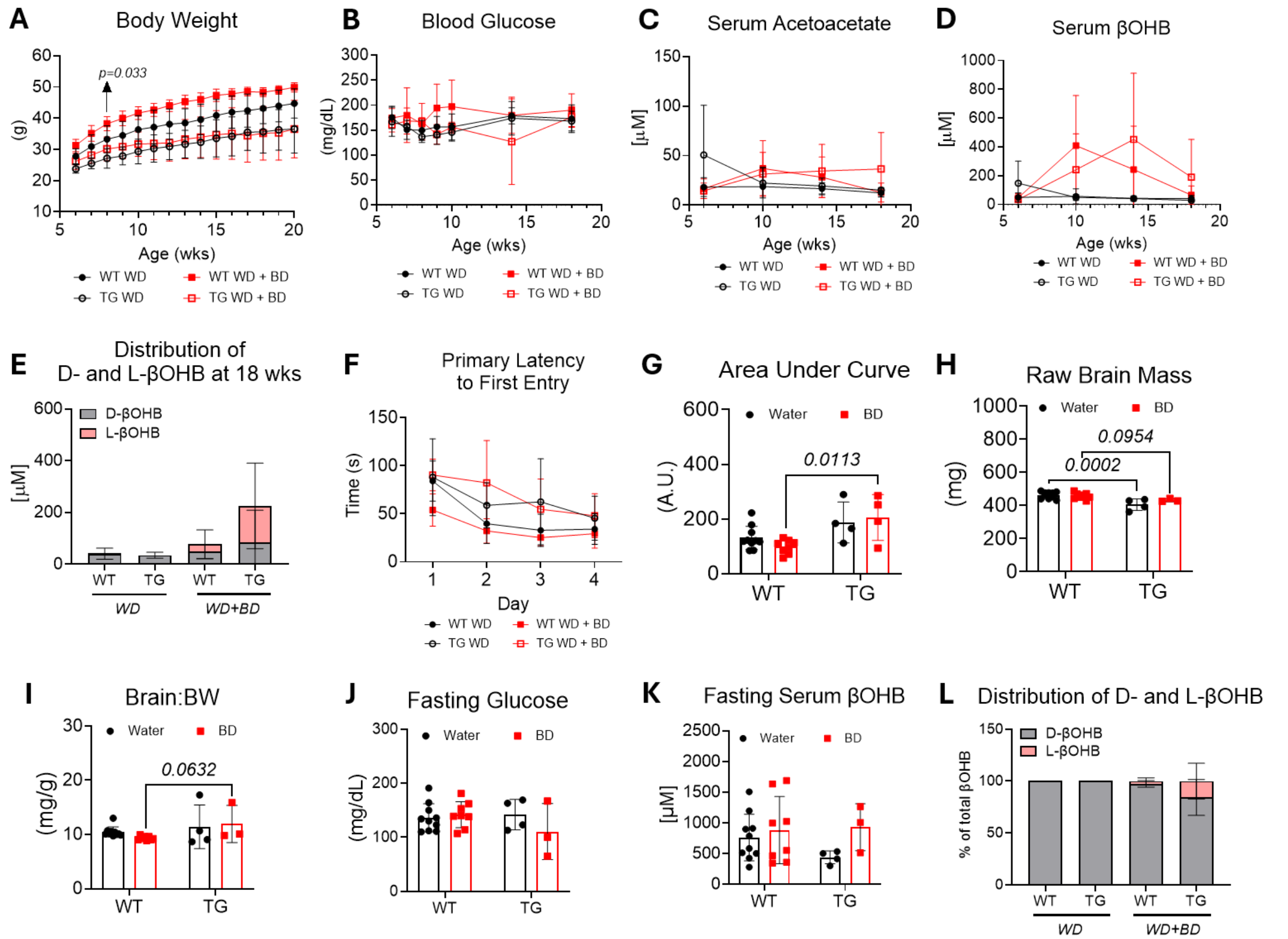

Supplemental Figure 10. High-fat, Western diet abolishes cognitive benefits of BD treatment in male mice. Male tauopathy mice and non-transgenic littermate controls, were also subjected to a 20-week high-fat Western diet while consuming 10% BD or normal drinking water. Cognitive function was assessed using a Barnes Maze before tissues were harvested for analyses. Effects of high-fat, Western diet with or without BD treatment on body weight (**A**) and non-fasted blood glucose (**B**) throughout the study. BD treatment effects on serum acetoacetate (**C**), serum βOHB (**D**), as well as the concentration and distribution of D- and L-βOHB at study midpoint (12-week treatment, 18 weeks of age, **E**). Latency to escape hole entry during memory acquisition phase, days 1-4 of Barnes Maze (**F**) with corresponding area under curve (**G**). Following cognitive testing, mice were euthanized, and the raw brain mass (**H**) was plotted with brain mass normalized to body weight (**I**). Following an 18 h fast, effects of BD treatment on fasted blood glucose (**J**), fasted serum βOHB (**K**), and distribution of D- and L-βOHB (**L**). Data are expressed as mean ± SD, *n*=3-10/group. P-values indicated when $p<0.10$ following two-way ANOVA and Tukey's post-hoc test. βOHB= β-hydroxybutyrate, WD = high-fat, Western diet, BD = 1,3-butanediol, A.U. = arbitrary units, WT = non-tauopathy control mice, TG = tauopathy mice.

bioRxiv preprint doi: https://doi.org/10.64898/2026.06.03.729999; this version posted June 8, 2026. The copyright holder for this preprint (which was not certified by peer review) is the author/funder, who has granted bioRxiv a license to display the preprint in perpetuity. It is made available under a CC-BY 4.0 International license.

Supplemental Figure 11

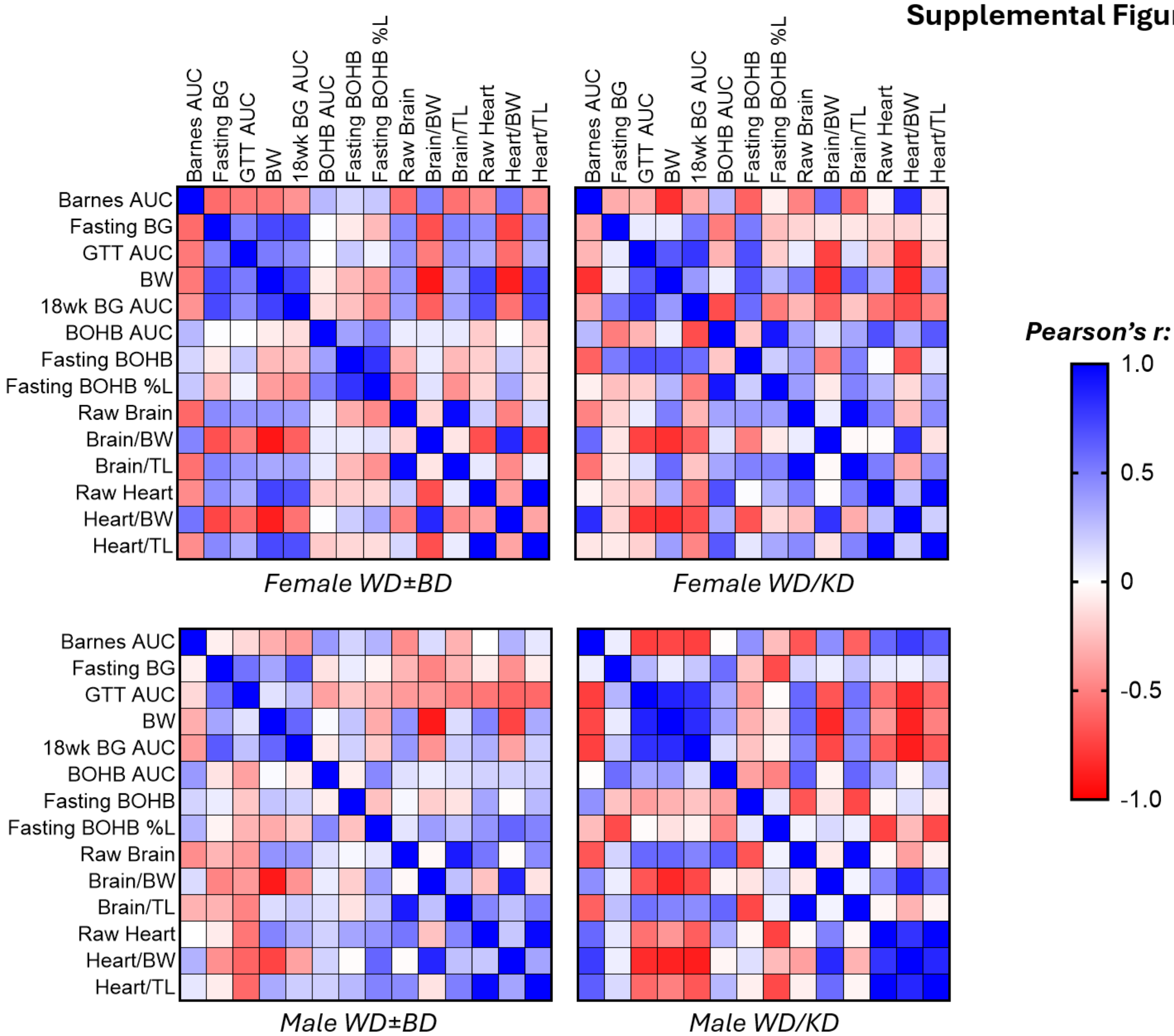

Supplemental Figure 11. Correlation heatmap plots for Western diet-fed mice. Heatmaps showing Pearson's r values for correlations in settings of Western diet (WD) with or without 10% BD in drinking water, and for weekly cycling of WD with a ketogenic diet (KD) for both male and female mice. Data are expressed as Pearson's r, *n*=10-26/group. AUC = area under the curve, BG = blood glucose, GTT = glucose tolerance test, BW = body weight, TL = tibia length for normalization.

bioRxiv preprint doi: https://doi.org/10.64898/2026.06.03.729999; this version posted June 8, 2026. The copyright holder for this preprint (which was not certified by peer review) is the author/funder, who has granted bioRxiv a license to display the preprint in perpetuity. It is made available under a CC-BY 4.0 International license.

Supplemental Figure 12

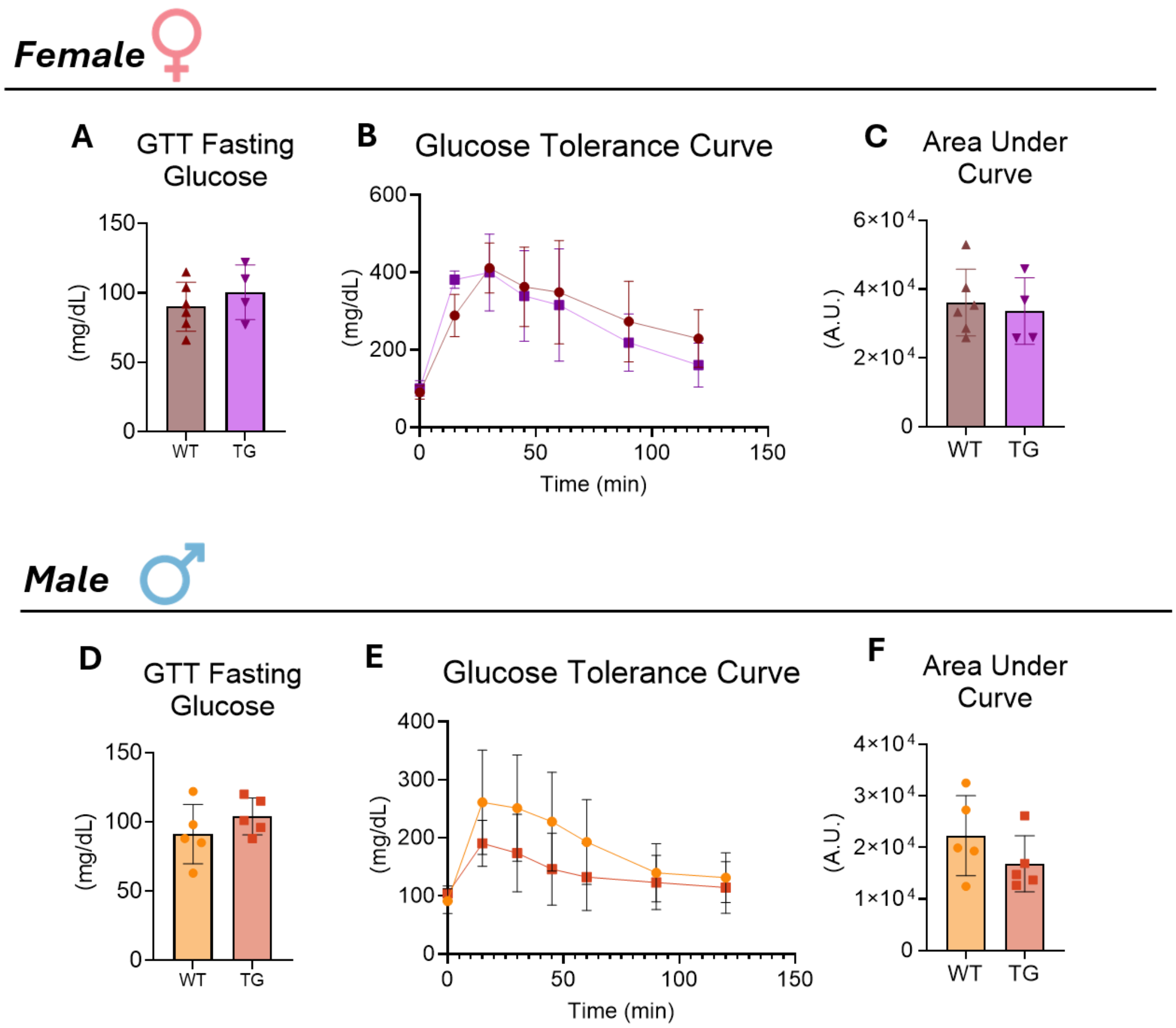

Supplemental Figure 12. Cycling a high-fat, ketogenic diet with a high-fat, Western diet has minimal effects on glucose tolerance in tauopathy male and female mice. Male and female mice were fed a 20-week dietary strategy that cycled a high-fat, Western diet weekly with a high-fat, very low-carbohydrate ketogenic diet. During a ketogenic diet week near the end of the study, mice were fasted for 18 h and then subjected to a glucose tolerance test (GTT) via I.P. injection of glucose (1 mg/g BW males; 2 mg/g BW females). Fasted blood glucose values (**A**) and glucose tolerance curve (**B**) with corresponding area under the curve (**C**) were recorded for female transgenic and non-transgenic mice. Fasted blood glucose values (**D**) and glucose tolerance curve (**E**) with corresponding area under the curve (**F**) were also recorded for male transgenic and non-transgenic mice. Data are expressed as mean ± SD, *n*=4-6/group. GTT = glucose tolerance test, A.U. = arbitrary units, WT = non-tauopathy control mice, TG = tauopathy mice

bioRxiv preprint doi: https://doi.org/10.64898/2026.06.03.729999; this version posted June 8, 2026. The copyright holder for this preprint (which was not certified by peer review) is the author/funder, who has granted bioRxiv a license to display the preprint in perpetuity. It is made available under a CC-BY 4.0 International license.

Supplemental Table 1: Genotyping primer sequences.

Supplemental Table 2: Pearson's correlation of metabolic variables with cognitive outcome in female mice.

Supplemental Table 3: Pearson's correlation of metabolic variables with cognitive outcome in male mice.

Supplemental Table 4: ANOVA results of meaningfully changed lipids in the brain.

Supplemental Table 5: Pearson's correlation of metabolic variables with cognitive outcomes in Western Diet-fed female mice.

Supplemental Table 6: Pearson's correlation of metabolic variables with cognitive outcomes in Western Diet-fed male mice.

Supplemental Table 7. Absolute concentrations of lipid species.

bioRxiv preprint doi: https://doi.org/10.64898/2026.06.03.729999; this version posted June 8, 2026. The copyright holder for this preprint (which was not certified by peer review) is the author/funder, who has granted bioRxiv a license to display the preprint in perpetuity. It is made available under a CC-BY 4.0 International license.

Gene	Forward Primer 5'-3'	Reverse Primer 5'-3'
Camk2a-tTA	5'-GAC CTG GAT GCT GAC GAA GG	5'-GCA GCT CTA ATG CGC TGT
MAPT*P301L	5'-GAT CTT AGC AAC GTC CAG TCC	5'-TGC CTA ATG AGC CAC ACT TG

Supplemental Table 1: Genotyping primer sequences. Forward and reverse primer sequences (5' – 3') to confirm tauopathy transgenic mice. Tauopathy mice are positive for both Camk2a-tTA and MAPT*P301L transgenes.

Pearson's r Correlations: 20 week NC +/- BD; Female

	**Barnes AUC**	**GTT AUC**	**Fasting BG**	**Raw Brain**	**BW**	**Brain/BW**	**Brain/TL**	**GTT Peak Glc**	**Raw Heart**	**Heart/BW**	**Heart/TL**	**Fasting BOHB**	**BOHB/AcAc**
**Barnes AUC**	1.000	-0.093	0.387	-0.586	-0.437	0.204	-0.597	-0.098	-0.432	0.265	-0.435	-0.288	-0.168
**GTT AUC**	-0.093	1.000	0.116	-0.017	0.383	-0.459	-0.128	0.810	0.323	-0.329	0.250	0.150	0.319
**Fasting BG**	0.387	0.116	1.000	-0.121	0.151	-0.258	-0.163	0.270	-0.071	-0.264	-0.099	-0.075	-0.099
**Raw Brain**	-0.586	-0.017	-0.121	1.000	0.556	-0.132	0.954	-0.125	0.350	-0.423	0.271	-0.114	-0.129
**BW**	-0.437	0.383	0.151	0.556	1.000	-0.880	0.379	0.278	0.643	-0.828	0.515	0.282	0.266
**Brain/BW**	0.204	-0.459	-0.258	-0.132	-0.880	1.000	0.061	-0.413	-0.524	0.813	-0.402	-0.362	-0.369
**Brain/TL**	-0.597	-0.128	-0.163	0.954	0.379	0.061	1.000	-0.158	0.240	-0.264	0.229	-0.063	-0.154
**GTT Peak Glc**	-0.098	0.810	0.270	-0.125	0.278	-0.413	-0.158	1.000	0.031	-0.398	0.007	0.383	0.367
**Raw Heart**	-0.432	0.323	-0.071	0.350	0.643	-0.524	0.240	0.031	1.000	-0.134	0.971	0.099	0.284
**Heart/BW**	0.265	-0.329	-0.264	-0.423	-0.828	0.813	-0.264	-0.398	-0.134	1.000	0.015	-0.288	-0.183
**Heart/TL**	-0.435	0.250	-0.099	0.271	0.515	-0.402	0.229	0.007	0.971	0.015	1.000	0.155	0.291
**Fasting BOHB**	-0.288	0.150	-0.075	-0.114	0.282	-0.362	-0.063	0.383	0.099	-0.288	0.155	1.000	0.532
**BOHB/AcAc**	-0.168	0.319	-0.099	-0.129	0.266	-0.369	-0.154	0.367	0.284	-0.183	0.291	0.532	1.000

Supplemental Table 2: Pearson's correlation of metabolic variables with cognitive outcome in female mice. Pearson's correlation coefficient, r, for various metabolic variables following a 20-week normal chow (NC) diet with or without 10% (v/v) 1,3-butanediol (BD) dissolved in drinking water. Positive values indicate positive correlation and negative values indicate negative correlation. *n*=29 females were used in correlation calculations. AUC = area under curve, GTT = glucose tolerance test, BG = blood glucose, BW = body weight, TL = tibia length, Glc = glucose, βOHB = total beta-hydroxybutyrate (both D and L enantiomers), AcAc = acetoacetate.

Pearson's r Correlations: 20 week NC +/- BD; Male

	Barnes AUC	GTT AUC	Fasting BG	Raw Brain	BW	Brain/BW	Brain/TL	GTT Peak Glc	Raw Heart	Heart/BW	Heart/TL	Fasting BOHB	BOHB/AcAc
**Barnes AUC**	1.000	0.056	0.204	-0.764	-0.525	0.298	-0.632	0.074	-0.218	0.608	-0.130	-0.432	-0.259
**GTT AUC**	0.056	1.000	0.327	0.270	0.145	-0.068	0.323	0.888	0.075	-0.131	0.111	-0.418	-0.279
**Fasting BG**	0.204	0.327	1.000	-0.096	0.365	-0.461	-0.295	0.512	0.467	-0.079	0.399	-0.523	-0.189
**Raw Brain**	-0.764	0.270	-0.096	1.000	0.572	-0.233	0.887	0.139	0.362	-0.513	0.294	0.189	0.112
**BW**	-0.525	0.145	0.365	0.572	1.000	-0.905	0.228	0.115	0.776	-0.783	0.620	-0.217	-0.086
**Brain/BW**	0.298	-0.068	-0.461	-0.233	-0.905	1.000	0.145	-0.137	-0.723	0.717	-0.558	0.323	0.225
**Brain/TL**	-0.632	0.323	-0.295	0.887	0.228	0.145	1.000	0.123	0.133	-0.186	0.190	0.280	0.176
**GTT Peak Glc**	0.074	0.888	0.512	0.139	0.115	-0.137	0.123	1.000	0.070	-0.097	0.071	-0.504	-0.429
**Raw Heart**	-0.218	0.075	0.467	0.362	0.776	-0.723	0.133	0.070	1.000	-0.229	0.959	-0.243	-0.163
**Heart/BW**	0.608	-0.131	-0.079	-0.513	-0.783	0.717	-0.186	-0.097	-0.229	1.000	-0.017	0.050	-0.001
**Heart/TL**	-0.130	0.111	0.399	0.294	0.620	-0.558	0.190	0.071	0.959	-0.017	1.000	-0.226	-0.158
**Fasting BOHB**	-0.432	-0.418	-0.523	0.189	-0.217	0.323	0.280	-0.504	-0.243	0.050	-0.226	1.000	0.687
**BOHB/AcAc**	-0.259	-0.279	-0.189	0.112	-0.086	0.225	0.176	-0.429	-0.163	-0.001	-0.158	0.687	1.000

Supplemental Table 3: Pearson's correlation of metabolic variables with cognitive outcome in male mice. Pearson's correlation coefficient, r, for various metabolic variables following a 20-week normal chow (NC) diet with or without 10% (v/v) 1,3-butanediol (BD) dissolved in drinking water. Positive values indicate positive correlation and negative values indicate negative correlation. *n*=23 males were used in correlation calculations. AUC = area under curve, GTT = glucose tolerance test, BG = blood glucose, BW = body weight, TL = tibia length, Glc = glucose, βOHB = total beta-hydroxybutyrate (both D and L enantiomers), AcAc = acetoacetate.

bioRxiv preprint doi: https://doi.org/10.64898/2026.06.03.729999; this version posted June 8, 2026. The copyright holder for this preprint (which was not certified by peer review) is the author/funder, who has granted bioRxiv a license to display the preprint in perpetuity. It is made available under a CC-BY 4.0 International license.

Lipid	Sex	term.x	p.value	contrast	std.error	df.y	adj.p.value
PS 18:1-18:2	F	TX	0.000535	TG BD - WT BD	0.023618	11	0.003998
PS 18:1-18:2	F	TX	0.000535	TG BD - TG W	0.023618	11	0.052279
PS 18:1-18:2	F	TX	0.000535	TG BD - WT W	0.025511	11	0.456682
PS 18:1-18:2	F	TX	0.000535	WT BD - TG W	0.023618	11	0.000292
PS 18:1-18:2	F	TX	0.000535	WT BD - WT W	0.025511	11	0.002713
PS 18:1-18:2	F	TX	0.000535	TG W - WT W	0.025511	11	0.207944
LYSOPC P16:0	F	TX	0.001235	TG BD - WT BD	0.129130	10	0.289465
LYSOPC P16:0	F	TX	0.001235	TG BD - TG W	0.129130	10	0.015814
LYSOPC P16:0	F	TX	0.001235	TG BD - WT W	0.158152	10	0.159361
LYSOPC P16:0	F	TX	0.001235	WT BD - TG W	0.129130	10	0.005299
LYSOPC P16:0	F	TX	0.001235	WT BD - WT W	0.158152	10	0.045509
LYSOPC P16:0	F	TX	0.001235	TG W - WT W	0.158152	10	0.289465
PS 18:1-18:2	F	TG	0.001260	TG BD - WT BD	0.023618	11	0.003998
PS 18:1-18:2	F	TG	0.001260	TG BD - TG W	0.023618	11	0.052279
PS 18:1-18:2	F	TG	0.001260	TG BD - WT W	0.025511	11	0.456682
PS 18:1-18:2	F	TG	0.001260	WT BD - TG W	0.023618	11	0.000292
PS 18:1-18:2	F	TG	0.001260	WT BD - WT W	0.025511	11	0.002713
PS 18:1-18:2	F	TG	0.001260	TG W - WT W	0.025511	11	0.207944
SM N16:0	F	TG	0.003854	TG BD - WT BD	0.069182	11	0.022260
SM N16:0	F	TG	0.003854	TG BD - TG W	0.069182	11	0.610205
SM N16:0	F	TG	0.003854	TG BD - WT W	0.074725	11	0.197420
SM N16:0	F	TG	0.003854	WT BD - TG W	0.069182	11	0.028117
SM N16:0	F	TG	0.003854	WT BD - WT W	0.074725	11	0.209816
SM N16:0	F	TG	0.003854	TG W - WT W	0.074725	11	0.257191
PS 18:2-18:2	F	TG	0.005265	TG BD - WT BD	0.048092	11	0.062875
PS 18:2-18:2	F	TG	0.005265	TG BD - TG W	0.048092	11	0.848852
PS 18:2-18:2	F	TG	0.005265	TG BD - WT W	0.051945	11	0.113324
PS 18:2-18:2	F	TG	0.005265	WT BD - TG W	0.048092	11	0.062875
PS 18:2-18:2	F	TG	0.005265	WT BD - WT W	0.051945	11	0.717002
PS 18:2-18:2	F	TG	0.005265	TG W - WT W	0.051945	11	0.115970
PE D22:6-24:6	F	TX	0.005326	TG BD - WT BD	0.201892	11	0.387333
PE D22:6-24:6	F	TX	0.005326	TG BD - TG W	0.201892	11	0.214064
PE D22:6-24:6	F	TX	0.005326	TG BD - WT W	0.218069	11	0.061274
PE D22:6-24:6	F	TX	0.005326	WT BD - TG W	0.201892	11	0.061274
PE D22:6-24:6	F	TX	0.005326	WT BD - WT W	0.218069	11	0.036282
PE D22:6-24:6	F	TX	0.005326	TG W - WT W	0.218069	11	0.356842
LYSOPE P16:1	F	TX	0.005685	TG BD - WT BD	0.128761	10	0.801232
LYSOPE P16:1	F	TX	0.005685	TG BD - TG W	0.128761	10	0.087251
LYSOPE P16:1	F	TX	0.005685	TG BD - WT W	0.157699	10	0.087251
LYSOPE P16:1	F	TX	0.005685	WT BD - TG W	0.128761	10	0.087251
LYSOPE P16:1	F	TX	0.005685	WT BD - WT W	0.157699	10	0.120163
LYSOPE P16:1	F	TX	0.005685	TG W - WT W	0.157699	10	0.932129
PE D18:1-18:2/D16:0-20:3	F	TX	0.010229	TG BD - WT BD	0.116059	11	0.195480
PE D18:1-18:2/D16:0-20:3	F	TX	0.010229	TG BD - TG W	0.116059	11	0.200356
PE D18:1-18:2/D16:0-20:3	F	TX	0.010229	TG BD - WT W	0.125359	11	0.339953
PE D18:1-18:2/D16:0-20:3	F	TX	0.010229	WT BD - TG W	0.116059	11	0.033777
PE D18:1-18:2/D16:0-20:3	F	TX	0.010229	WT BD - WT W	0.125359	11	0.051509
PE D18:1-18:2/D16:0-20:3	F	TX	0.010229	TG W - WT W	0.125359	11	0.717289
PI 16:0-18:0	F	TG:TX	0.011112	TG BD - WT BD	0.022624	11	0.435383
PI 16:0-18:0	F	TG:TX	0.011112	TG BD - TG W	0.022624	11	0.435383
PI 16:0-18:0	F	TG:TX	0.011112	TG BD - WT W	0.024437	11	0.091779
PI 16:0-18:0	F	TG:TX	0.011112	WT BD - TG W	0.022624	11	0.879141
PI 16:0-18:0	F	TG:TX	0.011112	WT BD - WT W	0.024437	11	0.028786
PI 16:0-18:0	F	TG:TX	0.011112	TG W - WT W	0.024437	11	0.028786
PE A20:0-20:4/P18:0-22:3	F	TX	0.011602	TG BD - WT BD	0.107563	11	0.654373
PE A20:0-20:4/P18:0-22:3	F	TX	0.011602	TG BD - TG W	0.107563	11	0.187814
PE A20:0-20:4/P18:0-22:3	F	TX	0.011602	TG BD - WT W	0.116182	11	0.087491
PE A20:0-20:4/P18:0-22:3	F	TX	0.011602	WT BD - TG W	0.107563	11	0.295490
PE A20:0-20:4/P18:0-22:3	F	TX	0.011602	WT BD - WT W	0.116182	11	0.093576
PE A20:0-20:4/P18:0-22:3	F	TX	0.011602	TG W - WT W	0.116182	11	0.307641
LYSOPC P20:4	F	TX	0.014342	TG BD - WT BD	0.109611	10	0.523815
LYSOPC P20:4	F	TX	0.014342	TG BD - TG W	0.109611	10	0.173425
LYSOPC P20:4	F	TX	0.014342	TG BD - WT W	0.134246	10	0.171909
LYSOPC P20:4	F	TX	0.014342	WT BD - TG W	0.109611	10	0.089079
LYSOPC P20:4	F	TX	0.014342	WT BD - WT W	0.134246	10	0.089079
LYSOPC P20:4	F	TX	0.014342	TG W - WT W	0.134246	10	0.629329
PC D18:0-20:2/P18:2-22:6	F	TG:TX	0.014892	TG BD - WT BD	0.145125	11	0.135474
PC D18:0-20:2/P18:2-22:6	F	TG:TX	0.014892	TG BD - TG W	0.145125	11	0.573642
PC D18:0-20:2/P18:2-22:6	F	TG:TX	0.014892	TG BD - WT W	0.156753	11	0.211317
PC D18:0-20:2/P18:2-22:6	F	TG:TX	0.014892	WT BD - TG W	0.145125	11	0.211317
PC D18:0-20:2/P18:2-22:6	F	TG:TX	0.014892	WT BD - WT W	0.156753	11	0.036088
PC D18:0-20:2/P18:2-22:6	F	TG:TX	0.014892	TG W - WT W	0.156753	11	0.135474

bioRxiv preprint doi: https://doi.org/10.64898/2026.06.03.729999; this version posted June 8, 2026. The copyright holder for this preprint (which was not certified by peer review) is the author/funder, who has granted bioRxiv a license to display the preprint in perpetuity. It is made available under a CC-BY 4.0 International license.

PE P18:0-20:3	F	TX	0.016734	TG BD - WT BD	0.150340	11	0.145588
PE P18:0-20:3	F	TX	0.016734	TG BD - TG W	0.150340	11	0.676816
PE P18:0-20:3	F	TX	0.016734	TG BD - WT W	0.162386	11	0.124412
PE P18:0-20:3	F	TX	0.016734	WT BD - TG W	0.150340	11	0.124412
PE P18:0-20:3	F	TX	0.016734	WT BD - WT W	0.162386	11	0.021557
PE P18:0-20:3	F	TX	0.016734	TG W - WT W	0.162386	11	0.145588
PC D18:0-20:3	F	TG:TX	0.022866	TG BD - WT BD	0.119137	11	0.118437
PC D18:0-20:3	F	TG:TX	0.022866	TG BD - TG W	0.119137	11	0.978675
PC D18:0-20:3	F	TG:TX	0.022866	TG BD - WT W	0.128683	11	0.118437
PC D18:0-20:3	F	TG:TX	0.022866	WT BD - TG W	0.119137	11	0.118437
PC D18:0-20:3	F	TG:TX	0.022866	WT BD - WT W	0.128683	11	0.023853
PC D18:0-20:3	F	TG:TX	0.022866	TG W - WT W	0.128683	11	0.118437
SM N20:1	F	TG:TX	0.023053	TG BD - WT BD	0.045240	11	0.311586
SM N20:1	F	TG:TX	0.023053	TG BD - TG W	0.045240	11	0.363824
SM N20:1	F	TG:TX	0.023053	TG BD - WT W	0.048865	11	0.363824
SM N20:1	F	TG:TX	0.023053	WT BD - TG W	0.045240	11	0.665941
SM N20:1	F	TG:TX	0.023053	WT BD - WT W	0.048865	11	0.149306
SM N20:1	F	TG:TX	0.023053	TG W - WT W	0.048865	11	0.154241
PC D18:0-20:3	F	TX	0.030340	TG BD - WT BD	0.119137	11	0.118437
PC D18:0-20:3	F	TX	0.030340	TG BD - TG W	0.119137	11	0.978675
PC D18:0-20:3	F	TX	0.030340	TG BD - WT W	0.128683	11	0.118437
PC D18:0-20:3	F	TX	0.030340	WT BD - TG W	0.119137	11	0.118437
PC D18:0-20:3	F	TX	0.030340	WT BD - WT W	0.128683	11	0.023853
PC D18:0-20:3	F	TX	0.030340	TG W - WT W	0.128683	11	0.118437
CARN 24:0	F	TX	0.030592	TG BD - WT BD	0.003697	10	0.444856
CARN 24:0	F	TX	0.030592	TG BD - TG W	0.003697	10	0.277506
CARN 24:0	F	TX	0.030592	TG BD - WT W	0.004528	10	0.277506
CARN 24:0	F	TX	0.030592	WT BD - TG W	0.003697	10	0.160314
CARN 24:0	F	TX	0.030592	WT BD - WT W	0.004528	10	0.160314
CARN 24:0	F	TX	0.030592	TG W - WT W	0.004528	10	0.822221
CHOLESTEROL	F	TX	0.032807	TG BD - WT BD	0.179436	11	0.090055
CHOLESTEROL	F	TX	0.032807	TG BD - TG W	0.179436	11	0.884253
CHOLESTEROL	F	TX	0.032807	TG BD - WT W	0.193814	11	0.306424
CHOLESTEROL	F	TX	0.032807	WT BD - TG W	0.179436	11	0.090055
CHOLESTEROL	F	TX	0.032807	WT BD - WT W	0.193814	11	0.033677
CHOLESTEROL	F	TX	0.032807	TG W - WT W	0.193814	11	0.306424
PI 18:0-22:6	F	TX	0.032900	TG BD - WT BD	0.081945	11	0.994171
PI 18:0-22:6	F	TX	0.032900	TG BD - TG W	0.081945	11	0.414664
PI 18:0-22:6	F	TX	0.032900	TG BD - WT W	0.088511	11	0.085694
PI 18:0-22:6	F	TX	0.032900	WT BD - TG W	0.081945	11	0.414664
PI 18:0-22:6	F	TX	0.032900	WT BD - WT W	0.088511	11	0.085694
PI 18:0-22:6	F	TX	0.032900	TG W - WT W	0.088511	11	0.273224
LYSOPC P18:0	F	TX	0.033019	TG BD - WT BD	0.132231	10	0.454230
LYSOPC P18:0	F	TX	0.033019	TG BD - TG W	0.132231	10	0.219554
LYSOPC P18:0	F	TX	0.033019	TG BD - WT W	0.161949	10	0.454230
LYSOPC P18:0	F	TX	0.033019	WT BD - TG W	0.132231	10	0.138762
LYSOPC P18:0	F	TX	0.033019	WT BD - WT W	0.161949	10	0.219554
LYSOPC P18:0	F	TX	0.033019	TG W - WT W	0.161949	10	0.674601
CHOLESTEROL	F	TG:TX	0.034220	TG BD - WT BD	0.179436	11	0.090055
CHOLESTEROL	F	TG:TX	0.034220	TG BD - TG W	0.179436	11	0.884253
CHOLESTEROL	F	TG:TX	0.034220	TG BD - WT W	0.193814	11	0.306424
CHOLESTEROL	F	TG:TX	0.034220	WT BD - TG W	0.179436	11	0.090055
CHOLESTEROL	F	TG:TX	0.034220	WT BD - WT W	0.193814	11	0.033677
CHOLESTEROL	F	TG:TX	0.034220	TG W - WT W	0.193814	11	0.306424
PE P18:0-20:3	F	TG:TX	0.034409	TG BD - WT BD	0.150340	11	0.145588
PE P18:0-20:3	F	TG:TX	0.034409	TG BD - TG W	0.150340	11	0.676816
PE P18:0-20:3	F	TG:TX	0.034409	TG BD - WT W	0.162386	11	0.124412
PE P18:0-20:3	F	TG:TX	0.034409	WT BD - TG W	0.150340	11	0.124412
PE P18:0-20:3	F	TG:TX	0.034409	WT BD - WT W	0.162386	11	0.021557
PE P18:0-20:3	F	TG:TX	0.034409	TG W - WT W	0.162386	11	0.145588
SM N20:0	F	TX	0.035854	TG BD - WT BD	0.194716	11	0.583336
SM N20:0	F	TX	0.035854	TG BD - TG W	0.194716	11	0.421529
SM N20:0	F	TX	0.035854	TG BD - WT W	0.210317	11	0.233896
SM N20:0	F	TX	0.035854	WT BD - TG W	0.194716	11	0.304354
SM N20:0	F	TX	0.035854	WT BD - WT W	0.210317	11	0.187800
SM N20:0	F	TX	0.035854	TG W - WT W	0.210317	11	0.421529
LYSOPC P14:1	F	TX	0.036326	TG BD - WT BD	0.167732	10	0.786733
LYSOPC P14:1	F	TX	0.036326	TG BD - TG W	0.167732	10	0.310127
LYSOPC P14:1	F	TX	0.036326	TG BD - WT W	0.205428	10	0.340251
LYSOPC P14:1	F	TX	0.036326	WT BD - TG W	0.167732	10	0.310127
LYSOPC P14:1	F	TX	0.036326	WT BD - WT W	0.205428	10	0.394947
LYSOPC P14:1	F	TX	0.036326	TG W - WT W	0.205428	10	0.786733
CARN 18:2	F	TX	0.037723	TG BD - WT BD	0.005045	10	0.564716

bioRxiv preprint doi: https://doi.org/10.64898/2026.06.03.729999; this version posted June 8, 2026. The copyright holder for this preprint (which was not certified by peer review) is the author/funder, who has granted bioRxiv a license to display the preprint in perpetuity. It is made available under a CC-BY 4.0 International license.

CARN 18:2	F	TX	0.037723	TG BD - TG W	0.005045	10	0.288543
CARN 18:2	F	TX	0.037723	TG BD - WT W	0.006179	10	0.368994
CARN 18:2	F	TX	0.037723	WT BD - TG W	0.005045	10	0.250626
CARN 18:2	F	TX	0.037723	WT BD - WT W	0.006179	10	0.284615
CARN 18:2	F	TX	0.037723	TG W - WT W	0.006179	10	0.952586
PC D18:1-20:4/D16:0-22:5	F	TX	0.038051	TG BD - WT BD	0.197059	11	0.787573
PC D18:1-20:4/D16:0-22:5	F	TX	0.038051	TG BD - TG W	0.197059	11	0.266527
PC D18:1-20:4/D16:0-22:5	F	TX	0.038051	TG BD - WT W	0.212848	11	0.266527
PC D18:1-20:4/D16:0-22:5	F	TX	0.038051	WT BD - TG W	0.197059	11	0.266527
PC D18:1-20:4/D16:0-22:5	F	TX	0.038051	WT BD - WT W	0.212848	11	0.266527
PC D18:1-20:4/D16:0-22:5	F	TX	0.038051	TG W - WT W	0.212848	11	0.787573
PC D18:0-18:2/D18:1-18:1	F	TG:TX	0.038378	TG BD - WT BD	0.133103	11	0.285337
PC D18:0-18:2/D18:1-18:1	F	TG:TX	0.038378	TG BD - TG W	0.133103	11	0.976841
PC D18:0-18:2/D18:1-18:1	F	TG:TX	0.038378	TG BD - WT W	0.143768	11	0.143105
PC D18:0-18:2/D18:1-18:1	F	TG:TX	0.038378	WT BD - TG W	0.133103	11	0.285337
PC D18:0-18:2/D18:1-18:1	F	TG:TX	0.038378	WT BD - WT W	0.143768	11	0.052829
PC D18:0-18:2/D18:1-18:1	F	TG:TX	0.038378	TG W - WT W	0.143768	11	0.143105
LYSOPC P16:0	F	TG	0.040553	TG BD - WT BD	0.129130	10	0.289465
LYSOPC P16:0	F	TG	0.040553	TG BD - TG W	0.129130	10	0.015814
LYSOPC P16:0	F	TG	0.040553	TG BD - WT W	0.158152	10	0.159361
LYSOPC P16:0	F	TG	0.040553	WT BD - TG W	0.129130	10	0.005299
LYSOPC P16:0	F	TG	0.040553	WT BD - WT W	0.158152	10	0.045509
LYSOPC P16:0	F	TG	0.040553	TG W - WT W	0.158152	10	0.289465
PE P16:0-20:4	F	TG:TX	0.041740	TG BD - WT BD	0.178932	11	0.262516
PE P16:0-20:4	F	TG:TX	0.041740	TG BD - TG W	0.178932	11	0.262516
PE P16:0-20:4	F	TG:TX	0.041740	TG BD - WT W	0.193269	11	0.817434
PE P16:0-20:4	F	TG:TX	0.041740	WT BD - TG W	0.178932	11	0.925116
PE P16:0-20:4	F	TG:TX	0.041740	WT BD - WT W	0.193269	11	0.323192
PE P16:0-20:4	F	TG:TX	0.041740	TG W - WT W	0.193269	11	0.323192
PS 16:0-18:1	F	TG	0.042394	TG BD - WT BD	0.166443	11	0.065690
PS 16:0-18:1	F	TG	0.042394	TG BD - TG W	0.166443	11	0.955379
PS 16:0-18:1	F	TG	0.042394	TG BD - WT W	0.179779	11	0.946636
PS 16:0-18:1	F	TG	0.042394	WT BD - TG W	0.166443	11	0.065690
PS 16:0-18:1	F	TG	0.042394	WT BD - WT W	0.179779	11	0.101032
PS 16:0-18:1	F	TG	0.042394	TG W - WT W	0.179779	11	0.946636
SM N24:0	F	TX	0.043113	TG BD - WT BD	0.121684	11	0.391265
SM N24:0	F	TX	0.043113	TG BD - TG W	0.121684	11	0.572440
SM N24:0	F	TX	0.043113	TG BD - WT W	0.131434	11	0.271516
SM N24:0	F	TX	0.043113	WT BD - TG W	0.121684	11	0.271516
SM N24:0	F	TX	0.043113	WT BD - WT W	0.131434	11	0.116247
SM N24:0	F	TX	0.043113	TG W - WT W	0.131434	11	0.358214
CARN 26:0-OH	F	TX	0.046506	TG BD - WT BD	0.002419	10	0.225406
CARN 26:0-OH	F	TX	0.046506	TG BD - TG W	0.002419	10	0.282729
CARN 26:0-OH	F	TX	0.046506	TG BD - WT W	0.002962	10	0.667757
CARN 26:0-OH	F	TX	0.046506	WT BD - TG W	0.002419	10	0.061981
CARN 26:0-OH	F	TX	0.046506	WT BD - WT W	0.002962	10	0.225406
CARN 26:0-OH	F	TX	0.046506	TG W - WT W	0.002962	10	0.592589
PI 18:0-22:4	F	TG	0.047210	TG BD - WT BD	0.030847	11	0.403935
PI 18:0-22:4	F	TG	0.047210	TG BD - TG W	0.030847	11	0.362730
PI 18:0-22:4	F	TG	0.047210	TG BD - WT W	0.033319	11	0.611698
PI 18:0-22:4	F	TG	0.047210	WT BD - TG W	0.030847	11	0.150747
PI 18:0-22:4	F	TG	0.047210	WT BD - WT W	0.033319	11	0.611698
PI 18:0-22:4	F	TG	0.047210	TG W - WT W	0.033319	11	0.262417
LYSOPC P18:2	M	TX	0.003945	TG BD - WT BD	0.066713	10	0.859884
LYSOPC P18:2	M	TX	0.003945	TG BD - TG W	0.059670	10	0.194437
LYSOPC P18:2	M	TX	0.003945	TG BD - WT W	0.066713	10	0.011970
LYSOPC P18:2	M	TX	0.003945	WT BD - TG W	0.059670	10	0.176435
LYSOPC P18:2	M	TX	0.003945	WT BD - WT W	0.066713	10	0.011970
LYSOPC P18:2	M	TX	0.003945	TG W - WT W	0.059670	10	0.048893
LYSOPE P18:0	M	TG:TX	0.006253	TG BD - WT BD	0.154988	10	0.059502
LYSOPE P18:0	M	TG:TX	0.006253	TG BD - TG W	0.138626	10	0.059502
LYSOPE P18:0	M	TG:TX	0.006253	TG BD - WT W	0.154988	10	0.997927
LYSOPE P18:0	M	TG:TX	0.006253	WT BD - TG W	0.138626	10	0.803344
LYSOPE P18:0	M	TG:TX	0.006253	WT BD - WT W	0.154988	10	0.059502
LYSOPE P18:0	M	TG:TX	0.006253	TG W - WT W	0.138626	10	0.059502
FFA 18:0	M	TX	0.010906	TG BD - WT BD	0.128579	11	0.234333
FFA 18:0	M	TX	0.010906	TG BD - TG W	0.122945	11	0.234333
FFA 18:0	M	TX	0.010906	TG BD - WT W	0.137457	11	0.306074
FFA 18:0	M	TX	0.010906	WT BD - TG W	0.112933	11	0.034511
FFA 18:0	M	TX	0.010906	WT BD - WT W	0.128579	11	0.051301
FFA 18:0	M	TX	0.010906	TG W - WT W	0.122945	11	0.841599
FFA 20:0	M	TG	0.014809	TG BD - WT BD	0.040946	11	0.144235
FFA 20:0	M	TG	0.014809	TG BD - TG W	0.039152	11	0.871681

bioRxiv preprint doi: https://doi.org/10.64898/2026.06.03.729999; this version posted June 8, 2026. The copyright holder for this preprint (which was not certified by peer review) is the author/funder, who has granted bioRxiv a license to display the preprint in perpetuity. It is made available under a CC-BY 4.0 International license.

FFA 20:0	M	TG	0.014809	TG BD - WT W	0.043773	11	0.144235
FFA 20:0	M	TG	0.014809	WT BD - TG W	0.035963	11	0.144235
FFA 20:0	M	TG	0.014809	WT BD - WT W	0.040946	11	0.871681
FFA 20:0	M	TG	0.014809	TG W - WT W	0.039152	11	0.144235
PS P20:0-22:5	M	TG	0.024942	TG BD - WT BD	0.028866	11	0.072705
PS P20:0-22:5	M	TG	0.024942	TG BD - TG W	0.027602	11	0.850439
PS P20:0-22:5	M	TG	0.024942	TG BD - WT W	0.030859	11	0.850439
PS P20:0-22:5	M	TG	0.024942	WT BD - TG W	0.025354	11	0.052345
PS P20:0-22:5	M	TG	0.024942	WT BD - WT W	0.028866	11	0.090453
PS P20:0-22:5	M	TG	0.024942	TG W - WT W	0.027602	11	0.850439
LYSOPE P20:4	M	TG:TX	0.025024	TG BD - WT BD	0.214730	10	0.310809
LYSOPE P20:4	M	TG:TX	0.025024	TG BD - TG W	0.192061	10	0.310809
LYSOPE P20:4	M	TG:TX	0.025024	TG BD - WT W	0.214730	10	0.505478
LYSOPE P20:4	M	TG:TX	0.025024	WT BD - TG W	0.192061	10	0.759340
LYSOPE P20:4	M	TG:TX	0.025024	WT BD - WT W	0.214730	10	0.135888
LYSOPE P20:4	M	TG:TX	0.025024	TG W - WT W	0.192061	10	0.135888
LYSOPC 14:1	M	TX	0.025341	TG BD - WT BD	0.105541	10	0.414359
LYSOPC 14:1	M	TX	0.025341	TG BD - TG W	0.094399	10	0.142381
LYSOPC 14:1	M	TX	0.025341	TG BD - WT W	0.105541	10	0.142381
LYSOPC 14:1	M	TX	0.025341	WT BD - TG W	0.094399	10	0.414359
LYSOPC 14:1	M	TX	0.025341	WT BD - WT W	0.105541	10	0.375843
LYSOPC 14:1	M	TX	0.025341	TG W - WT W	0.094399	10	0.677237
FFA 21:4	M	TG	0.027669	TG BD - WT BD	0.047026	11	0.205720
FFA 21:4	M	TG	0.027669	TG BD - TG W	0.044965	11	0.558489
FFA 21:4	M	TG	0.027669	TG BD - WT W	0.050272	11	0.205720
FFA 21:4	M	TG	0.027669	WT BD - TG W	0.041303	11	0.242362
FFA 21:4	M	TG	0.027669	WT BD - WT W	0.047026	11	0.982993
FFA 21:4	M	TG	0.027669	TG W - WT W	0.044965	11	0.242362
LYSOPE 18:0	M	TG:TX	0.029442	TG BD - WT BD	0.070987	10	0.351608
LYSOPE 18:0	M	TG:TX	0.029442	TG BD - TG W	0.063493	10	0.193187
LYSOPE 18:0	M	TG:TX	0.029442	TG BD - WT W	0.070987	10	0.784096
LYSOPE 18:0	M	TG:TX	0.029442	WT BD - TG W	0.063493	10	0.626511
LYSOPE 18:0	M	TG:TX	0.029442	WT BD - WT W	0.070987	10	0.305854
LYSOPE 18:0	M	TG:TX	0.029442	TG W - WT W	0.063493	10	0.193187
FFA 22:3	M	TG	0.030182	TG BD - WT BD	0.042362	11	0.145904
FFA 22:3	M	TG	0.030182	TG BD - TG W	0.040506	11	0.742198
FFA 22:3	M	TG	0.030182	TG BD - WT W	0.045287	11	0.742198
FFA 22:3	M	TG	0.030182	WT BD - TG W	0.037207	11	0.088041
FFA 22:3	M	TG	0.030182	WT BD - WT W	0.042362	11	0.230452
FFA 22:3	M	TG	0.030182	TG W - WT W	0.040506	11	0.606629
PE P16:0-22:6/D18:0-18:0/P18:2-20:4	M	TX	0.030923	TG BD - WT BD	0.216742	11	0.443627
PE P16:0-22:6/D18:0-18:0/P18:2-20:4	M	TX	0.030923	TG BD - TG W	0.207245	11	0.599539
PE P16:0-22:6/D18:0-18:0/P18:2-20:4	M	TX	0.030923	TG BD - WT W	0.231706	11	0.196067
PE P16:0-22:6/D18:0-18:0/P18:2-20:4	M	TX	0.030923	WT BD - TG W	0.190366	11	0.196067
PE P16:0-22:6/D18:0-18:0/P18:2-20:4	M	TX	0.030923	WT BD - WT W	0.216742	11	0.070706
PE P16:0-22:6/D18:0-18:0/P18:2-20:4	M	TX	0.030923	TG W - WT W	0.207245	11	0.196067
LYSOPE 20:4	M	TG:TX	0.032120	TG BD - WT BD	0.091440	10	0.258290
LYSOPE 20:4	M	TG:TX	0.032120	TG BD - TG W	0.081787	10	0.221748
LYSOPE 20:4	M	TG:TX	0.032120	TG BD - WT W	0.091440	10	0.936214
LYSOPE 20:4	M	TG:TX	0.032120	WT BD - TG W	0.081787	10	0.878835
LYSOPE 20:4	M	TG:TX	0.032120	WT BD - WT W	0.091440	10	0.258290
LYSOPE 20:4	M	TG:TX	0.032120	TG W - WT W	0.081787	10	0.221748
LYSOPE 16:1	M	TG	0.032816	TG BD - WT BD	0.059770	10	0.995759
LYSOPE 16:1	M	TG	0.032816	TG BD - TG W	0.053460	10	0.232015
LYSOPE 16:1	M	TG	0.032816	TG BD - WT W	0.059770	10	0.248545
LYSOPE 16:1	M	TG	0.032816	WT BD - TG W	0.053460	10	0.232015
LYSOPE 16:1	M	TG	0.032816	WT BD - WT W	0.059770	10	0.248545
LYSOPE 16:1	M	TG	0.032816	TG W - WT W	0.053460	10	0.053661
LYSOPC P22:6	M	TX	0.034539	TG BD - WT BD	0.145274	10	0.820926
LYSOPC P22:6	M	TX	0.034539	TG BD - TG W	0.129937	10	0.820926
LYSOPC P22:6	M	TX	0.034539	TG BD - WT W	0.145274	10	0.025077
LYSOPC P22:6	M	TX	0.034539	WT BD - TG W	0.129937	10	0.820926
LYSOPC P22:6	M	TX	0.034539	WT BD - WT W	0.145274	10	0.025077
LYSOPC P22:6	M	TX	0.034539	TG W - WT W	0.129937	10	0.025077
LYSOPC 20:0	M	TX	0.034596	TG BD - WT BD	0.143626	10	0.824476
LYSOPC 20:0	M	TX	0.034596	TG BD - TG W	0.128463	10	0.824476
LYSOPC 20:0	M	TX	0.034596	TG BD - WT W	0.143626	10	0.024952
LYSOPC 20:0	M	TX	0.034596	WT BD - TG W	0.128463	10	0.824476
LYSOPC 20:0	M	TX	0.034596	WT BD - WT W	0.143626	10	0.024952
LYSOPC 20:0	M	TX	0.034596	TG W - WT W	0.128463	10	0.024952
PS 18:0-20:0	M	TG	0.038108	TG BD - WT BD	0.056749	11	0.250422
PS 18:0-20:0	M	TG	0.038108	TG BD - TG W	0.054263	11	0.709033
PS 18:0-20:0	M	TG	0.038108	TG BD - WT W	0.060668	11	0.250422

bioRxiv preprint doi: https://doi.org/10.64898/2026.06.03.729999; this version posted June 8, 2026. The copyright holder for this preprint (which was not certified by peer review) is the author/funder, who has granted bioRxiv a license to display the preprint in perpetuity. It is made available under a CC-BY 4.0 International license.

PS 18:0-20:0	M	TG	0.038108	WT BD - TG W	0.049844	11	0.250422
PS 18:0-20:0	M	TG	0.038108	WT BD - WT W	0.056749	11	0.891343
PS 18:0-20:0	M	TG	0.038108	TG W - WT W	0.054263	11	0.293293
SM N20:0	M	TX	0.040460	TG BD - WT BD	0.189305	11	0.364170
SM N20:0	M	TX	0.040460	TG BD - TG W	0.181011	11	0.201102
SM N20:0	M	TX	0.040460	TG BD - WT W	0.202376	11	0.201102
SM N20:0	M	TX	0.040460	WT BD - TG W	0.166269	11	0.527799
SM N20:0	M	TX	0.040460	WT BD - WT W	0.189305	11	0.364170
SM N20:0	M	TX	0.040460	TG W - WT W	0.181011	11	0.527799
PI A18:0-22:3	M	TG	0.040585	TG BD - WT BD	0.022000	11	0.095164
PI A18:0-22:3	M	TG	0.040585	TG BD - TG W	0.021036	11	0.189119
PI A18:0-22:3	M	TG	0.040585	TG BD - WT W	0.023519	11	0.095164
PI A18:0-22:3	M	TG	0.040585	WT BD - TG W	0.019323	11	0.452666
PI A18:0-22:3	M	TG	0.040585	WT BD - WT W	0.022000	11	0.950571
PI A18:0-22:3	M	TG	0.040585	TG W - WT W	0.021036	11	0.452666
PC D18:0-20:1/P18:1-22:6	M	TG	0.040725	TG BD - WT BD	0.158270	11	0.670602
PC D18:0-20:1/P18:1-22:6	M	TG	0.040725	TG BD - TG W	0.151335	11	0.558294
PC D18:0-20:1/P18:1-22:6	M	TG	0.040725	TG BD - WT W	0.169197	11	0.253325
PC D18:0-20:1/P18:1-22:6	M	TG	0.040725	WT BD - TG W	0.139010	11	0.319955
PC D18:0-20:1/P18:1-22:6	M	TG	0.040725	WT BD - WT W	0.158270	11	0.280037
PC D18:0-20:1/P18:1-22:6	M	TG	0.040725	TG W - WT W	0.151335	11	0.090326
LYSOPC 22:6	M	TG:TX	0.040806	TG BD - WT BD	0.085862	10	0.424645
LYSOPC 22:6	M	TG:TX	0.040806	TG BD - TG W	0.076798	10	0.903130
LYSOPC 22:6	M	TG:TX	0.040806	TG BD - WT W	0.085862	10	0.133492
LYSOPC 22:6	M	TG:TX	0.040806	WT BD - TG W	0.076798	10	0.424645
LYSOPC 22:6	M	TG:TX	0.040806	WT BD - WT W	0.085862	10	0.075029
LYSOPC 22:6	M	TG:TX	0.040806	TG W - WT W	0.076798	10	0.107376
PS 18:1-22:6	M	TX	0.041060	TG BD - WT BD	0.192906	11	0.959385
PS 18:1-22:6	M	TX	0.041060	TG BD - TG W	0.184454	11	0.272689
PS 18:1-22:6	M	TX	0.041060	TG BD - WT W	0.206226	11	0.272689
PS 18:1-22:6	M	TX	0.041060	WT BD - TG W	0.169432	11	0.272689
PS 18:1-22:6	M	TX	0.041060	WT BD - WT W	0.192906	11	0.272689
PS 18:1-22:6	M	TX	0.041060	TG W - WT W	0.184454	11	0.695597
LYSOPE P16:0	M	TG:TX	0.045161	TG BD - WT BD	0.115592	10	0.466185
LYSOPE P16:0	M	TG:TX	0.045161	TG BD - TG W	0.103388	10	0.491772
LYSOPE P16:0	M	TG:TX	0.045161	TG BD - WT W	0.115592	10	0.466185
LYSOPE P16:0	M	TG:TX	0.045161	WT BD - TG W	0.103388	10	0.615706
LYSOPE P16:0	M	TG:TX	0.045161	WT BD - WT W	0.115592	10	0.201219
LYSOPE P16:0	M	TG:TX	0.045161	TG W - WT W	0.103388	10	0.201219
PC D18:0-20:2/P18:2-22:6	M	TX	0.047000	TG BD - WT BD	0.180405	11	0.234358
PC D18:0-20:2/P18:2-22:6	M	TX	0.047000	TG BD - TG W	0.172500	11	0.234358
PC D18:0-20:2/P18:2-22:6	M	TX	0.047000	TG BD - WT W	0.192861	11	0.110410
PC D18:0-20:2/P18:2-22:6	M	TX	0.047000	WT BD - TG W	0.158452	11	0.887694
PC D18:0-20:2/P18:2-22:6	M	TX	0.047000	WT BD - WT W	0.180405	11	0.234358
PC D18:0-20:2/P18:2-22:6	M	TX	0.047000	TG W - WT W	0.172500	11	0.234358
PS 18:0-22:6...139	M	TX	0.047179	TG BD - WT BD	0.223230	11	0.730131
PS 18:0-22:6...139	M	TX	0.047179	TG BD - TG W	0.213448	11	0.438238
PS 18:0-22:6...139	M	TX	0.047179	TG BD - WT W	0.238642	11	0.272536
PS 18:0-22:6...139	M	TX	0.047179	WT BD - TG W	0.196065	11	0.272536
PS 18:0-22:6...139	M	TX	0.047179	WT BD - WT W	0.223230	11	0.272536
PS 18:0-22:6...139	M	TX	0.047179	TG W - WT W	0.213448	11	0.602235
PS 18:0-22:6...158	M	TX	0.047179	TG BD - WT BD	0.223230	11	0.730131
PS 18:0-22:6...158	M	TX	0.047179	TG BD - TG W	0.213448	11	0.438238
PS 18:0-22:6...158	M	TX	0.047179	TG BD - WT W	0.238642	11	0.272536
PS 18:0-22:6...158	M	TX	0.047179	WT BD - TG W	0.196065	11	0.272536
PS 18:0-22:6...158	M	TX	0.047179	WT BD - WT W	0.223230	11	0.272536
PS 18:0-22:6...158	M	TX	0.047179	TG W - WT W	0.213448	11	0.602235
LYSOPE 16:0	M	TG:TX	0.048299	TG BD - WT BD	0.079026	10	0.262780
LYSOPE 16:0	M	TG:TX	0.048299	TG BD - TG W	0.070683	10	0.262780
LYSOPE 16:0	M	TG:TX	0.048299	TG BD - WT W	0.079026	10	0.632918
LYSOPE 16:0	M	TG:TX	0.048299	WT BD - TG W	0.070683	10	0.900213
LYSOPE 16:0	M	TG:TX	0.048299	WT BD - WT W	0.079026	10	0.365703
LYSOPE 16:0	M	TG:TX	0.048299	TG W - WT W	0.070683	10	0.365703
PI 18:0-22:6	M	TG	0.048390	TG BD - WT BD	0.107479	11	0.262899
PI 18:0-22:6	M	TG	0.048390	TG BD - TG W	0.102770	11	0.548242
PI 18:0-22:6	M	TG	0.048390	TG BD - WT W	0.114900	11	0.262899
PI 18:0-22:6	M	TG	0.048390	WT BD - TG W	0.094400	11	0.332859
PI 18:0-22:6	M	TG	0.048390	WT BD - WT W	0.107479	11	0.836794
PI 18:0-22:6	M	TG	0.048390	TG W - WT W	0.102770	11	0.332859
PI 18:2-20:4/16:0-22:6	M	TG	0.048909	TG BD - WT BD	0.070808	11	0.266370
PI 18:2-20:4/16:0-22:6	M	TG	0.048909	TG BD - TG W	0.067705	11	0.476756
PI 18:2-20:4/16:0-22:6	M	TG	0.048909	TG BD - WT W	0.075697	11	0.278940
PI 18:2-20:4/16:0-22:6	M	TG	0.048909	WT BD - TG W	0.062191	11	0.278940

bioRxiv preprint doi: https://doi.org/10.64898/2026.06.03.729999; this version posted June 8, 2026. The copyright holder for this preprint (which was not certified by peer review) is the author/funder, who has granted bioRxiv a license to display the preprint in perpetuity. It is made available under a CC-BY 4.0 International license.

PI 18:2-20:4/16:0-22:6	M	TG	0.048909	WT BD - WT W	0.070808	11	0.582821
PI 18:2-20:4/16:0-22:6	M	TG	0.048909	TG W - WT W	0.067705	11	0.476756
LYSOPC 20:4	M	TG:TX	0.049207	TG BD - WT BD	0.127010	10	0.328583
LYSOPC 20:4	M	TG:TX	0.049207	TG BD - TG W	0.113601	10	0.287838
LYSOPC 20:4	M	TG:TX	0.049207	TG BD - WT W	0.127010	10	0.963501
LYSOPC 20:4	M	TG:TX	0.049207	WT BD - TG W	0.113601	10	0.861471
LYSOPC 20:4	M	TG:TX	0.049207	WT BD - WT W	0.127010	10	0.328583
LYSOPC 20:4	M	TG:TX	0.049207	TG W - WT W	0.113601	10	0.287838

Supplemental Table 4: ANOVA results of meaningfully changed lipids in the brain. Absolute lipid concentrations in the brain were measured and compared within female or male cohorts. Data reflect two-way ANOVA with estimated marginal means and Benjamini-Hochberg false-discovery. term.x = specific model of interaction being tested, p.value = raw p-value for the interaction following ANOVA, contrast = specific pairwise comparison performed, std.error = standard error of estimated contrast, df.y = degrees of freedom associated with statistical test, adj.p.value = multiple-testing correction of p-value (q), F = female, M = male TG = transgenic, WT = nontransgenic, TX = treatment, W = water, BD = 1,3-butanediol, PC = phosphatidylcholine, PE = phosphatidylethanolamine, PA = phosphatidic acid, PI = phosphatidylinositol, PS = phosphatidylserine, SM = sphingomyelin, CBS = cerebroside, FFA = free fatty-acid, LPC = lysoPC, LPE = lysoPE, Car = acylcarnitine.

Pearson's r Correlations: 20 week WD +/- BD; Female

	Barnes AUC	Fasting BG	GTT AUC	BW	18wk BG AUC	BOHB AUC	Fasting BOHB	Fasting BOHB %L	Raw Brain	Brain/BW	Brain/TL	Raw Heart	Heart/BW	Heart/TL
**Barnes AUC**	1.000	-0.582	-0.529	-0.528	-0.427	0.278	0.164	0.219	-0.592	0.481	-0.557	-0.458	0.543	-0.448
**Fasting BG**	-0.582	1.000	0.494	0.716	0.716	0.006	-0.086	-0.274	0.458	-0.690	0.483	0.441	-0.730	0.464
**GTT AUC**	-0.529	0.494	1.000	0.516	0.445	0.002	0.209	0.052	0.415	-0.516	0.399	0.326	-0.574	0.323
**BW**	-0.528	0.716	0.516	1.000	0.744	-0.078	-0.274	-0.385	0.418	-0.916	0.340	0.734	-0.882	0.712
**18wk BG AUC**	-0.427	0.716	0.445	0.744	1.000	-0.134	-0.248	-0.430	0.386	-0.629	0.360	0.684	-0.557	0.690
**BOHB AUC**	0.278	0.006	0.002	-0.078	-0.134	1.000	0.366	0.508	0.071	0.082	0.083	-0.203	0.005	-0.207
**Fasting BOHB**	0.164	-0.086	0.209	-0.274	-0.248	0.366	1.000	0.798	-0.319	0.075	-0.276	-0.192	0.200	-0.160
**Fasting BOHB %L**	0.219	-0.274	0.052	-0.385	-0.430	0.508	0.798	1.000	-0.462	0.110	-0.435	-0.168	0.338	-0.139
**Raw Brain**	-0.592	0.458	0.415	0.418	0.386	0.071	-0.319	-0.462	1.000	-0.164	0.975	0.192	-0.492	0.155
**Brain/BW**	0.481	-0.690	-0.516	-0.916	-0.629	0.082	0.075	0.110	-0.164	1.000	-0.102	-0.694	0.850	-0.691
**Brain/TL**	-0.557	0.483	0.399	0.340	0.360	0.083	-0.276	-0.435	0.975	-0.102	1.000	0.089	-0.463	0.077
**Raw Heart**	-0.458	0.441	0.326	0.734	0.684	-0.203	-0.192	-0.168	0.192	-0.694	0.089	1.000	-0.375	0.992
**Heart/BW**	0.543	-0.730	-0.574	-0.882	-0.557	0.005	0.200	0.338	-0.492	0.850	-0.463	-0.375	1.000	-0.360
**Heart/TL**	-0.448	0.464	0.323	0.712	0.690	-0.207	-0.160	-0.139	0.155	-0.691	0.077	0.992	-0.360	1.000

Pearson's r Correlations: 20 week WD cycled weekly with KD; Female

	Barnes AUC	Fasting BG	GTT AUC	BW	18wk BG AUC	BOHB AUC	Fasting BOHB	Fasting BOHB %L	Raw Brain	Brain/BW	Brain/TL	Raw Heart	Heart/BW	Heart/TL
**Barnes AUC**	1.000	-0.326	-0.292	-0.810	-0.336	0.272	-0.617	-0.063	-0.490	0.586	-0.546	-0.054	0.824	-0.099
**Fasting BG**	-0.326	1.000	0.080	0.074	0.529	-0.512	0.515	-0.253	-0.169	-0.102	-0.105	-0.164	-0.166	-0.089
**GTT AUC**	-0.292	0.080	1.000	0.653	0.788	-0.294	0.692	-0.194	0.078	-0.744	0.133	-0.236	-0.795	-0.183
**BW**	-0.810	0.074	0.653	1.000	0.398	0.070	0.666	0.289	0.503	-0.815	0.583	0.316	-0.830	0.378
**18wk BG AUC**	-0.336	0.529	0.788	0.398	1.000	-0.699	0.576	-0.516	-0.287	-0.621	-0.233	-0.547	-0.701	-0.479
**BOHB AUC**	0.272	-0.512	-0.294	0.070	-0.699	1.000	-0.221	0.914	0.355	0.114	0.347	0.685	0.312	0.655
**Fasting BOHB**	-0.617	0.515	0.692	0.666	0.576	-0.221	1.000	0.197	0.392	-0.499	0.492	0.010	-0.674	0.096
**Fasting BOHB %L**	-0.063	-0.253	-0.194	0.289	-0.516	0.914	0.197	1.000	0.386	-0.088	0.489	0.286	-0.152	0.340
**Raw Brain**	-0.490	-0.169	0.078	0.503	-0.287	0.355	0.392	0.386	1.000	0.072	0.988	0.501	-0.254	0.455
**Brain/BW**	0.586	-0.102	-0.744	-0.815	-0.621	0.114	-0.499	-0.088	0.072	1.000	-0.026	-0.018	0.802	-0.117
**Brain/TL**	-0.546	-0.105	0.133	0.583	-0.233	0.347	0.492	0.489	0.988	-0.026	1.000	0.507	-0.328	0.485
**Raw Heart**	-0.054	-0.164	-0.236	0.316	-0.547	0.685	0.010	0.286	0.501	-0.018	0.507	1.000	0.257	0.987
**Heart/BW**	0.824	-0.166	-0.795	-0.830	-0.701	0.312	-0.674	-0.152	-0.254	0.802	-0.328	0.257	1.000	0.191
**Heart/TL**	-0.099	-0.089	-0.183	0.378	-0.479	0.655	0.096	0.340	0.455	-0.117	0.485	0.987	0.191	1.000

Supplemental Table 5: Pearson's correlation of metabolic variables with cognitive outcome in Western Diet-fed female mice. Pearson's correlation coefficient, r, for various metabolic variables following a 20-week Western diet (WD) with or without 10% BD dissolved in drinking water, or WD cycling weekly with ketogenic diet (KD). Positive values indicate positive correlation and negative values indicate negative correlation. *n*=10-21 females in correlation calculations. BD = 1,3-butanediol, AUC = area under curve, GTT = glucose tolerance test, BG = blood glucose, BW = body weight, TL = tibia length, Glc = glucose, βOHB = total beta-hydroxybutyrate (both D and L enantiomers), AcAc = acetoacetate.

Pearson's r Correlations: 20 week WD +/- BD; Male

	Barnes AUC	Fasting BG	GTT AUC	BW	18wk BG AUC	BOHB AUC	Fasting BOHB	Fasting BOHB %L	Raw Brain	Brain/BW	Brain/TL	Raw Heart	Heart/BW	Heart/TL
**Barnes AUC**	1.000	-0.065	-0.156	-0.318	-0.394	0.396	0.172	0.295	-0.450	0.138	-0.304	-0.003	0.299	0.092
**Fasting BG**	-0.065	1.000	0.552	0.350	0.647	-0.113	0.074	-0.048	-0.291	-0.484	-0.300	-0.082	-0.439	-0.076
**GTT AUC**	-0.156	0.552	1.000	0.116	0.245	-0.372	-0.220	-0.294	-0.396	-0.403	-0.489	-0.539	-0.609	-0.588
**BW**	-0.318	0.350	0.116	1.000	0.597	0.016	0.228	-0.333	0.420	-0.903	0.135	0.481	-0.735	0.333
**18wk BG AUC**	-0.394	0.647	0.245	0.597	1.000	-0.079	0.182	-0.210	0.404	-0.425	0.193	0.313	-0.368	0.194
**BOHB AUC**	0.396	-0.113	-0.372	0.016	-0.079	1.000	-0.067	0.466	0.120	0.076	0.123	0.180	0.176	0.185
**Fasting BOHB**	0.172	0.074	-0.220	0.228	0.182	-0.067	1.000	-0.244	0.029	-0.195	-0.112	0.351	-0.014	0.274
**Fasting BOHB %L**	0.295	-0.048	-0.294	-0.333	-0.210	0.466	-0.244	1.000	0.105	0.384	0.237	0.417	0.602	0.485
**Raw Brain**	-0.450	-0.291	-0.396	0.420	0.404	0.120	0.029	0.105	1.000	-0.029	0.894	0.536	-0.019	0.451
**Brain/BW**	0.138	-0.484	-0.403	-0.903	-0.425	0.076	-0.195	0.384	-0.029	1.000	0.244	-0.240	0.854	-0.110
**Brain/TL**	-0.304	-0.300	-0.489	0.135	0.193	0.123	-0.112	0.237	0.894	0.244	1.000	0.475	0.247	0.500
**Raw Heart**	-0.003	-0.082	-0.539	0.481	0.313	0.180	0.351	0.417	0.536	-0.240	0.475	1.000	0.217	0.970
**Heart/BW**	0.299	-0.439	-0.609	-0.735	-0.368	0.176	-0.014	0.602	-0.019	0.854	0.247	0.217	1.000	0.355
**Heart/TL**	0.092	-0.076	-0.588	0.333	0.194	0.185	0.274	0.485	0.451	-0.110	0.500	0.970	0.355	1.000

Pearson's r Correlations: 20 week WD cycled weekly with KD; Male

	Barnes AUC	Fasting BG	GTT AUC	BW	18wk BG AUC	TKB AUC	Fasting BOHB	Fasting BOHB %L	Raw Brain	Brain/BW	Brain/TL	Raw Heart	Heart/BW	Heart/TL
**Barnes AUC**	1.000	0.068	-0.760	-0.722	-0.750	-0.010	0.428	-0.267	-0.664	0.442	-0.621	0.592	0.761	0.630
**Fasting BG**	0.068	1.000	0.289	0.082	0.221	0.572	-0.243	-0.709	0.171	0.068	0.249	0.093	0.065	0.146
**GTT AUC**	-0.760	0.289	1.000	0.864	0.808	0.334	-0.379	-0.018	0.588	-0.667	0.550	-0.557	-0.832	-0.592
**BW**	-0.722	0.082	0.864	1.000	0.828	0.384	-0.304	-0.119	0.592	-0.845	0.479	-0.415	-0.868	-0.501
**18wk BG AUC**	-0.750	0.221	0.808	0.828	1.000	0.143	-0.235	-0.063	0.482	-0.720	0.448	-0.632	-0.888	-0.662
**TKB AUC**	-0.010	0.572	0.334	0.384	0.143	1.000	-0.371	-0.491	0.624	-0.054	0.597	0.309	-0.040	0.277
**Fasting BOHB**	0.428	-0.243	-0.379	-0.304	-0.235	-0.371	1.000	0.097	-0.668	-0.110	-0.721	-0.038	0.125	-0.064
**Fasting BOHB %L**	-0.267	-0.709	-0.018	-0.119	-0.063	-0.491	0.097	1.000	0.047	0.148	0.067	-0.744	-0.271	-0.717
**Raw Brain**	-0.664	0.171	0.588	0.592	0.482	0.624	-0.668	0.047	1.000	-0.083	0.981	-0.031	-0.373	-0.065
**Brain/BW**	0.442	0.068	-0.667	-0.845	-0.720	-0.054	-0.110	0.148	-0.083	1.000	0.045	0.487	0.840	0.574
**Brain/TL**	-0.621	0.249	0.550	0.479	0.448	0.597	-0.721	0.067	0.981	0.045	1.000	-0.038	-0.299	-0.045
**Raw Heart**	0.592	0.093	-0.557	-0.415	-0.632	0.309	-0.038	-0.744	-0.031	0.487	-0.038	1.000	0.796	0.991
**Heart/BW**	0.761	0.065	-0.832	-0.868	-0.888	-0.040	0.125	-0.271	-0.373	0.840	-0.299	0.796	1.000	0.850
**Heart/TL**	0.630	0.146	-0.592	-0.501	-0.662	0.277	-0.064	-0.717	-0.065	0.574	-0.045	0.991	0.850	1.000

Supplemental Table 6: Pearson's correlation of metabolic variables with cognitive outcome in Western Diet-fed male mice. Pearson's correlation coefficient, r, for various metabolic variables following a 20-week Western diet (WD) with or without 10% BD dissolved in drinking water, or WD cycling weekly with ketogenic diet (KD). Positive values indicate positive correlation and negative values indicate negative correlation. *n*=10-26 males in correlation calculations. BD = 1,3-butanediol, AUC = area under curve, GTT = glucose tolerance test, BG = blood glucose, BW = body weight, TL = tibia length, Glc = glucose, βOHB = total beta-hydroxybutyrate (both D and L enantiomers), AcAc = acetoacetate.

Sex	M	M	M	M	M	M	M	M	M	M	M	M	M	M	M	M	F	F	F	F	F	F	F	F	F	F	F	F	F	F	F	F
Genotype	WT	WT	WT	WT	WT	WT	WT	WT	TG	TG	TG	TG	TG	TG	TG	TG	WT	WT	WT	WT	WT	WT	WT	WT	TG	TG	TG	TG	TG	TG	TG	TG
Water	W	W	W	W	BD	BD	BD	BD	W	W	W	W	W	BD	BD	BD	W	W	W	W	BD	BD	BD	BD	W	W	W	W	BD	BD	BD	BD
PE P16:0-18:2	0.333	0.363	0.317	0.477	0.440	0.851	0.348	0.451	0.827	0.239	0.143	0.615	0.410	0.520	0.480	0.205	1.390	0.531	1.150	0.221	0.211	0.413	0.159	0.661	0.317	0.335	0.995	0.474	0.363	0.486	1.115	0.434
PE P18:1-16:0/P16:0-18:1	9.580	13.231	6.857	16.979	8.232	15.800	8.956	9.271	11.995	9.709	6.602	9.662	10.567	12.500	10.486	6.046	14.192	14.369	20.422	8.937	6.804	7.877	5.218	11.027	5.911	8.020	16.318	7.519	7.861	10.397	12.535	8.938
PE P18:0-16:0/P16:0-18:0	1.485	2.246	1.236	1.783	1.387	2.071	1.425	1.468	2.255	1.672	0.692	1.710	1.645	1.354	1.995	1.968	1.972	1.937	1.643	1.612	2.037	0.972	0.535	2.170	1.354	1.387	2.804	1.404	1.955	1.899	2.184	1.606
PE D16:0-18:2/D16:1-18:1	0.876	0.994	0.776	0.990	0.621	0.877	0.579	0.728	0.859	0.700	0.640	0.797	1.124	0.992	0.829	0.635	0.963	0.924	1.399	0.690	0.754	0.510	0.302	0.669	0.630	0.716	1.382	0.577	0.561	0.851	1.178	0.796
PE D16:0-18:1	5.027	5.586	3.905	5.357	3.778	5.595	4.748	4.564	5.792	5.227	4.087	5.358	5.015	4.764	4.637	4.325	5.674	6.464	6.695	4.821	4.442	3.323	3.586	4.801	4.361	4.361	6.896	4.115	3.631	4.956	5.285	4.531
PE P16:0-20:4	4.543	3.056	5.047	2.590	4.644	10.231	3.878	3.852	6.078	4.842	5.433	3.487	3.720	5.371	4.646	3.993	5.478	3.962	5.125	6.648	3.741	3.660	4.148	5.214	3.454	4.149	3.448	5.604	6.337	4.361	4.921	6.691
PE P16:0-20:3/P18:1-18:2	1.335	1.381	1.336	1.466	0.939	2.167	1.030	1.030	1.987	1.059	0.899	1.307	1.360	1.203	0.998	1.098	2.101	1.761	2.747	1.517	1.434	0.991	0.531	1.415	1.163	1.572	2.058	1.368	1.447	1.174	1.939	1.265
PE P18:1-18:1/P18:0-18:2/P16:0-20:2	15.762	25.536	12.489	26.647	10.897	35.454	18.338	17.638	21.065	19.703	11.053	17.746	19.874	21.112	22.886	13.296	28.439	24.689	37.248	18.542	14.984	16.058	9.518	20.196	10.788	12.482	30.518	14.698	11.871	22.336	22.186	17.358
PE P18:0-18:1/P16:0-20:1	11.425	17.812	9.323	27.711	8.005	24.148	13.015	12.832	13.322	14.705	9.030	12.119	14.822	14.695	15.186	8.920	16.861	19.627	24.551	11.876	9.790	11.148	7.633	13.755	8.706	8.144	23.472	10.284	7.676	16.499	13.601	12.490
PE A18:0-18:1	0.640	0.886	0.680	2.198	0.327	1.370	0.722	0.700	0.548	0.873	0.238	0.741	1.038	1.404	0.992	0.528	1.352	1.270	1.774	0.558	0.397	0.815	0.320	0.785	0.447	0.295	1.386	0.493	0.343	0.944	0.567	0.493
PE D16:0-20:4/D18:2-18:2	3.192	3.436	3.793	2.571	3.249	3.881	2.899	2.810	3.440	3.597	4.006	2.878	3.350	3.560	2.808	3.106	3.465	3.297	3.042	3.385	3.931	1.782	2.483	2.883	3.098	2.556	3.541	3.525	3.655	3.276	3.267	3.513
PE D18:1-18:2/D16:0-20:3	0.591	0.749	0.998	1.216	0.263	0.950	0.619	0.731	0.664	0.444	0.707	1.134	0.836	0.808	0.717	0.367	0.991	0.940	0.882	0.621	0.450	0.471	0.322	0.434	0.824	0.539	1.344	0.852	0.435	0.710	0.650	0.787
PE D18:0-18:2/D18:1-18:1/D16:0-20:2	5.236	7.489	4.487	8.721	3.736	7.822	6.387	5.314	6.843	6.537	4.055	6.783	5.636	6.394	5.087	4.156	7.286	8.045	9.757	4.821	4.322	4.142	3.441	5.110	4.953	4.793	8.294	4.024	3.842	5.570	6.060	4.745
PE D18:0-18:1/D16:0-20:1	6.336	9.925	6.396	8.745	4.446	9.886	7.051	6.445	8.379	7.360	5.400	6.876	7.672	6.674	6.663	5.468	9.464	9.625	11.150	6.773	5.929	5.259	4.217	6.804	5.684	5.006	12.298	5.467	4.715	7.348	6.699	6.168
PE P16:0-22:6/D18:0-18:0/P18:2-20:4	19.730	12.580	15.002	8.859	22.734	23.380	17.970	14.393	18.415	15.833	18.210	12.205	12.879	19.573	15.477	15.128	21.679	12.846	13.878	17.945	19.361	11.182	13.985	19.352	13.212	14.638	13.643	16.659	16.573	14.339	20.634	20.433
PE P18:1-20:4/P16:0-22:5	10.891	8.606	8.204	9.236	5.781	16.895	8.393	9.363	12.289	11.681	12.200	7.796	9.208	11.621	11.526	8.411	14.673	11.927	17.464	10.362	9.333	7.142	7.993	11.441	7.120	9.588	15.257	9.741	7.719	11.902	14.137	11.221
PE P18:0-20:4/P16:0-22:4/P18:1-20:3	16.518	15.387	17.388	14.881	12.227	26.229	13.576	15.158	17.546	20.173	16.179	14.069	16.253	18.472	15.948	16.576	16.082	16.709	20.055	17.768	17.078	11.499	13.850	17.267	13.510	13.755	19.367	17.563	14.769	17.995	15.203	17.822
PE P18:0-20:3	2.340	2.525	2.735	3.162	2.165	3.889	1.999	2.399	2.739	2.673	3.115	2.061	2.562	3.226	2.521	2.537	2.934	3.134	4.474	3.057	2.276	1.707	1.551	2.484	2.214	2.841	3.792	2.287	2.581	2.929	2.369	2.433
PE P20:1-18:1/P18:1-20:1	4.758	9.340	4.167	16.216	3.527	13.355	6.917	5.911	5.700	8.319	3.702	5.934	7.627	6.249	7.835	5.347	8.304	9.365	12.860	6.731	4.224	5.429	3.134	6.873	3.561	3.464	11.468	4.778	3.303	8.595	5.735	6.051
PE P18:1-20:0	2.670	5.124	2.511	10.956	1.799	7.834	3.949	3.577	2.969	4.748	2.806	3.490	4.629	3.411	4.127	3.146	4.055	5.421	6.869	3.428	2.628	3.320	1.774	3.780	2.510	1.744	7.111	2.810	1.784	5.303	2.807	3.393
PE D16:1-22:6	0.642	0.235	0.485	0.318	0.595	0.339	0.649	0.324	0.495	0.502	0.712	0.526	0.444	1.196	0.156	0.470	0.928	0.249	0.375	0.464	0.981	0.270	0.280	0.605	0.488	0.371	0.592	0.356	0.506	0.263	0.642	0.538
PE D16:0-22:6	12.966	9.968	9.847	9.596	9.304	12.963	15.454	10.277	11.456	11.137	13.345	11.073	10.585	10.561	11.324	12.373	15.470	9.281	9.157	11.050	11.442	7.544	10.758	13.740	11.207	9.025	9.681	10.737	8.966	9.517	13.720	12.209
PE D18:1-20:4D/16:0-22:5	4.460	4.533	4.252	5.529	2.768	6.683	3.978	3.599	4.525	5.131	5.246	4.018	4.884	4.671	4.554	3.865	4.688	4.906	6.082	4.492	4.277	3.268	3.616	4.087	4.473	3.390	6.608	4.679	3.656	4.790	4.353	4.716
PE D18:0-20:4/D16:0-22:4	25.221	24.240	31.250	16.982	22.041	38.350	21.172	24.586	25.001	28.813	28.353	18.981	22.275	28.065	20.563	24.854	22.147	22.376	21.834	27.632	33.337	17.180	21.825	22.380	20.732	19.610	22.758	24.117	26.145	23.401	18.059	26.676
PE D18:0-20:3/D18:1-20:2/D16:0-22:3	0.927	0.985	1.730	1.249	1.038	1.714	0.810	1.168	1.398	1.032	1.179	1.361	1.093	0.917	0.856	1.140	1.513	0.850	1.102	0.782	0.565	0.945	0.872	0.848	0.994	0.751	1.363	1.212	0.746	1.241	1.102	0.615
PE P18:2-22:6/D18:1-20:1	0.411	0.664	0.588	1.717	0.301	1.235	0.688	0.355	0.600	0.583	0.383	0.378	0.453	0.383	0.350	0.282	0.974	0.674	1.332	0.385	0.445	0.432	0.243	0.529	0.273	0.205	1.166	0.335	0.037	0.519	0.511	0.293
PE P18:1-22:6/D18:0-20:1	5.948	3.912	3.932	5.755	2.399	8.458	5.489	4.430	4.722	5.936	7.026	3.368	4.460	4.336	6.925	5.636	7.638	5.661	7.023	6.109	6.657	4.440	5.107	7.487	4.102	3.682	8.354	6.647	2.995	5.902	7.054	7.363
PE P18:0-22:6/P18:1-22:5/D18:0-20:0	24.829	29.051	22.421	23.789	14.789	31.778	26.821	23.072	24.366	28.730	27.873	22.824	22.671	23.402	22.395	26.269	26.794	25.884	28.411	23.020	30.114	17.433	22.905	28.648	21.387	19.722	27.091	22.494	18.608	23.135	22.510	25.271
PE P18:0-22:5/P18:1-22:4	5.749	6.773	5.164	9.832	3.241	11.121	5.632	5.889	5.760	8.040	6.432	5.445	6.429	7.167	6.845	5.734	7.690	8.022	9.727	7.213	6.510	4.693	4.642	7.618	5.311	5.200	10.851	6.099	3.970	7.923	6.150	6.871
PE P18:0-22:4/P20:0-20:4/P18:1-22:3	5.867	6.544	6.834	9.113	4.467	9.823	4.740	6.748	5.743	8.857	6.877	5.794	7.234	7.227	6.071	6.690	4.695	7.468	7.633	7.408	7.142	4.079	5.668	5.884	5.778	4.946	9.230	6.762	4.773	8.016	4.617	7.055
PE A20:0-20:4/P18:0-22:3	0.617	0.821	1.064	1.312	0.567	1.206	0.431	0.974	0.920	0.835	0.978	1.042	1.073	1.324	0.552	0.681	0.282	1.023	1.235	0.955	0.964	0.529	0.617	0.698	0.887	1.022	1.069	0.574	0.806	0.748	0.438	0.584
PE P18:0-22:2	0.146	0.640	0.646	1.721	0.276	0.845	0.389	0.478	0.515	0.378	0.080	0.499	0.318	0.418	0.502	0.277	0.606	0.470	0.861	0.410	0.325	0.319	0.106	0.408	0.283	0.112	1.126	0.242	0.391	0.455	0.358	0.294

PE D18:1-22:6	3.034	2.357	2.356	2.903	2.256	3.862	3.044	2.346	2.588	3.180	3.280	2.145	2.464	3.531	3.036	2.767	3.456	2.911	3.486	3.218	3.252	1.890	2.696	3.315	2.849	2.183	3.107	3.466	1.834	3.244	3.528	3.484
PE D18:0-22:6/D18:1-22:5	37.603	37.197	30.271	33.513	24.794	42.360	47.355	32.897	34.117	37.101	40.556	35.540	34.229	30.453	33.992	37.179	41.978	36.913	35.875	34.092	36.105	26.157	33.077	41.074	34.784	27.367	37.968	31.292	25.917	31.392	34.671	35.213
PE D18:0-22:5/D18:1-22:4	1.735	2.082	1.885	1.623	1.348	2.071	1.986	2.023	1.778	1.723	1.415	1.705	1.500	2.050	1.696	1.997	2.609	1.771	1.759	2.141	2.101	1.360	1.546	2.290	1.623	1.357	1.848	1.703	1.421	1.671	1.547	1.706
PE D20:0-20:4/D18:0-22:4	3.225	3.136	3.867	3.314	2.817	4.658	2.961	3.305	2.947	4.705	3.445	2.712	3.126	4.541	2.794	3.635	2.371	3.345	3.007	4.509	3.640	2.139	3.103	3.334	3.869	2.474	3.496	3.294	2.437	3.517	1.629	3.417
PE D20:6-22:7	0.205	0.134	0.577	0.555	0.335	0.493	0.293	0.270	0.257	0.576	0.606	0.433	0.280	0.309	0.067	0.314	0.239	0.128	0.316	0.295	0.512	0.089	0.138	0.163	0.317	0.106	0.572	0.362	0.255	0.210	0.138	0.197
PE D20:2-22:6/D20:4-22:4	0.471	0.415	0.687	0.641	0.623	0.508	0.838	0.358	0.789	0.814	1.231	0.810	0.332	1.318	0.632	0.626	0.984	0.473	0.601	0.257	1.098	0.462	0.827	0.541	0.860	0.758	0.869	0.503	0.617	1.048	0.622	0.716
PE P22:6-22:7	3.050	3.433	2.971	2.861	3.241	6.092	4.544	2.377	4.167	4.096	3.547	3.734	2.948	6.136	4.381	3.730	7.524	4.788	4.339	2.914	4.354	4.183	3.129	4.336	2.943	3.284	4.779	2.470	3.093	5.035	4.791	2.976
PE D22:6-22:4	0.551	0.576	0.672	0.641	0.381	1.153	0.805	0.523	0.568	0.944	1.066	0.656	0.468	0.753	0.872	0.552	1.413	0.785	0.741	0.579	1.038	0.734	0.669	0.716	0.857	0.308	1.273	0.915	0.660	1.099	0.734	0.765
PE D22:6-24:6	10.256	6.711	10.164	3.650	10.124	14.150	12.922	6.070	10.134	10.888	13.671	8.939	5.966	12.960	10.243	12.838	14.607	7.286	5.433	8.450	16.203	8.951	12.848	12.121	10.394	10.135	5.907	7.862	12.736	9.563	10.543	10.221
PE D22:6-24:4	0.263	0.160	0.900	0.148	0.506	0.550	0.296	0.301	0.138	0.800	0.826	0.264	0.291	0.786	0.435	0.586	0.599	0.342	0.186	0.423	0.930	0.422	0.626	0.454	0.491	0.567	0.545	0.261	0.453	0.691	0.156	0.367
CHOLESTEROL	428.619	516.347	455.642	535.625	652.550	469.588	553.127	462.576	689.553	764.916	709.672	455.609	524.658	492.904	452.746	492.234	407.803	557.687	550.013	851.900	471.153	374.178	414.934	360.057	580.101	551.580	583.824	468.808	507.830	677.473	405.281	583.074
FFA 12:0	0.387	0.410	0.383	0.401	0.281	0.413	0.308	0.331	0.415	0.342	0.380	0.306	0.455	0.386	0.408	0.420	0.120	0.310	0.295	0.361	0.457	0.295	0.297	0.369	0.202	0.377	0.457	0.342	0.332	0.398	0.341	0.390
FFA 14:0	1.476	1.069	1.012	1.267	0.913	1.252	1.186	0.765	1.134	1.126	1.297	0.930	1.237	0.991	1.157	0.821	0.939	1.050	1.110	1.022	1.029	0.818	1.305	0.907	0.864	0.960	1.015	1.084	0.981	1.059	1.183	0.966
FFA 15:0	0.622	0.269	0.399	0.207	0.362	0.328	0.540	0.129	0.363	0.525	0.342	0.227	0.374	0.363	0.438	0.230	0.254	0.283	0.351	0.484	0.373	0.412	0.385	0.283	0.200	0.377	0.400	0.396	0.324	0.283	0.390	0.413
FFA 16:1	1.438	1.246	1.215	0.847	1.107	1.830	1.198	0.429	1.659	0.957	1.288	0.945	1.319	0.910	0.863	1.202	0.861	1.215	1.004	1.035	1.143	0.882	2.034	0.731	1.119	1.064	0.619	1.339	0.830	1.333	1.229	0.912
FFA 16:0	20.948	14.454	17.805	14.108	17.383	24.509	18.477	15.035	15.549	15.472	15.822	14.843	16.608	19.245	16.636	15.429	19.631	12.879	18.776	20.710	15.368	13.794	23.090	13.004	14.156	14.889	14.211	20.039	14.771	14.511	16.394	18.123
FFA 17:1	0.245	0.325	0.230	0.203	0.342	0.235	0.106	0.074	0.314	0.163	0.198	0.238	0.235	0.252	0.173	0.142	0.306	0.176	0.179	0.135	0.115	0.210	0.312	0.174	0.126	0.075	0.031	0.202	0.266	0.242	0.144	0.181
FFA 17:0	0.838	0.366	0.629	0.420	0.525	0.734	0.586	0.430	0.599	0.525	0.596	0.440	0.489	0.617	0.479	0.427	0.760	0.404	0.726	0.716	0.461	0.570	0.966	0.457	0.495	0.437	0.428	0.878	0.464	0.533	0.504	0.719
FFA 18:2	3.576	3.059	3.192	2.897	2.805	4.237	2.893	3.075	4.118	2.767	2.774	2.677	3.338	3.089	3.412	2.906	2.377	2.962	3.194	3.401	2.810	2.710	4.050	2.634	3.219	3.086	2.646	3.319	2.458	3.607	3.083	3.117
FFA 18:1	17.772	15.408	16.077	13.746	15.103	24.277	16.036	15.592	18.525	14.301	14.577	14.842	15.750	14.465	15.763	15.519	15.091	15.491	18.174	17.791	15.869	14.995	23.013	14.345	17.206	14.964	13.489	16.933	14.585	19.444	17.357	17.167
FFA 18:0	12.940	9.630	12.356	9.088	11.735	17.191	12.951	12.581	9.876	9.609	10.918	11.024	9.087	12.391	10.802	11.774	13.539	8.598	13.355	14.566	11.785	10.009	18.212	9.887	9.525	9.831	10.405	13.381	10.636	8.849	11.284	12.966
FFA 19:2	0.302	0.159	0.300	0.213	0.336	0.338	0.103	0.085	0.433	0.175	0.221	0.223	0.314	0.267	0.302	0.219	0.231	0.192	0.112	0.228	0.188	0.104	0.189	0.162	0.262	0.175	0.035	0.347	0.205	0.243	0.163	0.136
FFA 19:1	0.922	0.483	0.461	0.590	0.634	0.755	0.452	0.449	0.819	0.508	0.530	0.388	0.698	0.432	0.773	0.462	0.393	0.499	0.509	0.553	0.580	0.429	0.418	0.405	0.506	0.609	0.346	0.472	0.499	0.810	0.493	0.457
FFA 19:0	1.867	1.024	1.394	0.892	1.060	1.345	0.995	0.552	1.571	0.957	1.401	0.698	1.391	0.808	0.960	0.912	1.457	0.964	0.904	1.197	0.860	0.952	1.030	0.699	1.251	0.777	0.472	1.210	0.890	1.319	1.111	0.998
FFA 20:5	0.185	0.259	0.234	0.346	0.304	0.308	0.233	0.176	0.251	0.265	0.157	0.276	0.319	0.278	0.257	0.215	0.579	0.216	0.204	0.229	0.332	0.378	0.297	0.222	0.426	0.231	0.217	0.206	0.298	0.293	0.264	0.231
FFA 20:4	9.729	6.311	7.738	6.353	6.684	16.503	8.450	10.349	8.879	8.383	7.379	5.940	6.423	6.971	9.549	9.935	8.538	6.363	7.334	9.021	11.034	7.152	9.501	6.578	7.803	7.186	8.540	8.690	7.442	8.943	8.903	9.092
FFA 20:3	0.190	0.114	0.160	0.209	0.174	0.701	0.215	0.221	0.285	0.199	0.235	0.218	0.216	0.250	0.248	0.333	0.122	0.125	0.213	0.234	0.271	0.188	0.205	0.141	0.253	0.212	0.287	0.217	0.162	0.268	0.264	0.235
FFA 20:1	0.297	0.364	0.118	0.360	0.296	0.858	0.264	0.238	0.413	0.320	0.276	0.186	0.240	0.304	0.323	0.272	0.337	0.357	0.342	0.342	0.230	0.308	0.361	0.270	0.229	0.301	0.212	0.307	0.241	0.383	0.297	0.293
FFA 20:0	0.198	0.198	0.207	0.275	0.266	0.227	0.143	0.297	0.150	0.161	0.204	0.156	0.166	0.204	0.129	0.137	0.181	0.165	0.234	0.201	0.179	0.169	0.198	0.183	0.097	0.222	0.143	0.324	0.139	0.168	0.177	0.160
FFA 21:5	0.157	0.134	0.199	0.153	0.118	0.232	0.057	0.117	0.170	0.138	0.148	0.139	0.145	0.125	0.169	0.111	0.392	0.124	0.116	0.167	0.080	0.090	0.138	0.141	0.270	0.075	0.024	0.140	0.127	0.156	0.139	0.091
FFA 21:4	0.186	0.166	0.218	0.210	0.332	0.168	0.160	0.144	0.184	0.144	0.106	0.125	0.159	0.143	0.090	0.118	0.238	0.116	0.070	0.127	0.117	0.137	0.126	0.132	0.176	0.141	0.081	0.104	0.207	0.113	0.128	0.090
FFA 21:3	0.452	0.422	0.470	0.197	0.712	0.551	0.476	0.245	0.717	0.340	0.201	0.541	0.426	0.372	0.174	0.518	0.156	0.175	0.160	0.316	0.460	0.196	0.316	0.326	0.358	0.372	0.120	0.301	0.676	0.294	0.329	0.259
FFA 22:6	4.118	1.818	2.088	2.378	0.781	3.156	2.081	2.197	2.572	1.370	2.145	1.461	1.966	1.885	3.824	2.368	2.478	2.224	4.146	2.953	2.599	1.288	2.121	1.922	2.365	1.533	2.027	2.974	0.964	3.033	2.621	2.169
FFA 22:4	0.224	0.134	0.153	0.234	0.129	0.598	0.141	0.225	0.278	0.182	0.219	0.144	0.192	0.173	0.245	0.225	0.283	0.207	0.185	0.213	0.190	0.105	0.164	0.213	0.147	0.217	0.106	0.290	0.133	0.310	0.215	0.080
FFA 22:3	0.129	0.189	0.205	0.123	0.315	0.199	0.223	0.196	0.207	0.118	0.120	0.136	0.139	0.206	0.152	0.107	0.178	0.113	0.109	0.200	0.197	0.107	0.123	0.164	0.226	0.225	0.120	0.186	0.177	0.196	0.099	0.099

FFA 22:2	0.373	0.425	0.587	0.171	0.537	0.320	0.404	0.284	0.468	0.290	0.200	0.502	0.311	0.379	0.226	0.425	0.381	0.115	0.086	0.323	0.403	0.193	0.247	0.396	0.291	0.412	0.066	0.302	0.533	0.309	0.245	0.165
FFA 23:6	0.356	0.300	0.538	0.332	0.439	0.379	0.289	0.232	0.563	0.201	0.465	0.235	0.367	0.349	0.340	0.409	0.435	0.444	0.138	0.363	0.243	0.142	0.298	0.274	0.317	0.204	0.134	0.503	0.176	0.217	0.303	0.166
FFA 23:5	0.333	0.262	0.270	0.217	0.212	0.155	0.300	0.169	0.390	0.149	0.104	0.300	0.231	0.257	0.120	0.234	0.290	0.118	0.116	0.206	0.163	0.090	0.167	0.269	0.228	0.174	0.096	0.244	0.219	0.217	0.192	0.267
FFA 24:4	0.256	0.242	0.220	0.166	0.168	0.200	0.164	0.024	0.387	0.112	0.100	0.220	0.262	0.178	0.083	0.223	0.092	0.136	0.091	0.192	0.221	0.128	0.155	0.215	0.240	0.131	0.054	0.289	0.143	0.134	0.199	0.132
SM N16:0	0.800	0.422	0.799	0.886	0.724	1.013	0.553	0.686	1.042	0.679	0.709	0.661	1.086	0.996	0.676	0.710	0.879	0.652	0.808	0.668	0.672	0.423	0.556	0.655	0.744	0.945	0.964	0.679	0.729	0.893	1.000	0.890
SM N18:1	1.884	1.706	3.006	1.078	1.368	3.103	1.996	2.322	2.655	2.292	2.391	1.673	1.883	1.444	1.479	2.414	2.129	1.406	1.908	2.114	2.250	1.375	1.866	2.508	1.699	1.992	1.433	1.661	2.626	1.468	1.994	2.050
SM N18:0	15.089	15.291	19.659	18.339	11.315	22.121	12.360	19.064	17.175	18.668	17.615	14.191	18.958	14.991	11.003	16.634	14.082	14.166	19.729	17.571	17.771	9.373	13.738	18.993	16.385	16.124	15.970	19.318	14.685	13.400	13.302	24.352
SM N20:1	0.099	0.102	0.161	0.110	0.063	0.254	0.119	0.200	0.113	0.122	0.105	0.116	0.219	0.075	0.065	0.119	0.174	0.143	0.181	0.265	0.109	0.033	0.096	0.144	0.055	0.107	0.179	0.102	0.090	0.152	0.196	0.158
SM N20:0	0.894	1.921	1.013	1.746	0.612	1.481	1.158	1.473	0.993	1.675	1.117	1.283	1.659	0.646	0.645	1.172	1.104	1.821	2.276	1.226	0.814	0.669	0.787	1.473	1.238	1.010	2.008	1.267	0.524	1.163	1.141	1.572
SM N22:1	0.238	0.309	0.234	0.404	0.224	0.703	0.289	0.690	0.494	0.376	0.247	0.321	0.394	0.254	0.153	0.204	0.679	0.315	0.951	0.366	0.176	0.242	0.182	0.605	0.210	0.320	0.623	0.386	0.218	0.398	0.621	0.436
SM N22:0	0.327	0.477	0.220	0.577	0.251	0.763	0.367	0.601	0.403	0.428	0.287	0.455	0.577	0.235	0.272	0.323	0.578	0.502	0.868	0.466	0.174	0.286	0.259	0.602	0.284	0.353	0.802	0.546	0.219	0.380	0.629	0.394
SM N24:2	0.120	0.221	0.135	0.179	0.071	0.308	0.168	0.282	0.164	0.191	0.208	0.281	0.206	0.065	0.097	0.181	0.194	0.190	0.292	0.194	0.110	0.089	0.142	0.267	0.207	0.187	0.270	0.241	0.112	0.119	0.147	0.358
SM N24:1	1.375	3.506	1.533	3.930	0.972	3.225	1.998	2.805	2.057	2.783	1.527	2.246	3.924	1.025	1.669	1.546	1.824	2.784	5.472	2.621	0.720	1.227	1.044	2.824	1.487	1.455	5.345	2.314	0.903	2.041	2.319	3.139
SM N24:0	0.129	0.433	0.188	0.583	0.216	0.354	0.177	0.561	0.307	0.354	0.250	0.336	0.571	0.233	0.221	0.257	0.330	0.479	0.639	0.432	0.198	0.143	0.055	0.344	0.245	0.199	0.624	0.378	0.089	0.225	0.354	0.520
CBS N18:0	0.923	1.323	0.636	1.611	0.610	1.846	1.178	1.170	1.417	0.704	0.728	0.870	1.255	1.066	0.753	0.752	1.797	1.089	2.477	1.105	0.632	0.613	0.455	1.544	0.509	0.722	1.546	1.228	0.962	1.053	1.744	1.011
CBS OH_N18:0	0.937	1.448	0.855	1.365	0.790	1.274	0.852	0.781	1.192	0.751	0.540	0.729	1.126	1.170	1.075	0.607	1.664	1.432	2.077	0.912	0.629	0.695	0.500	1.094	0.530	1.099	2.064	0.451	0.897	1.055	1.407	0.931
CBS N20:0	0.402	0.394	0.376	0.500	0.051	0.456	0.383	0.621	0.566	0.277	0.202	0.471	0.349	0.358	0.288	0.321	0.878	0.581	0.857	0.346	0.202	0.194	0.221	0.440	0.102	0.380	0.783	0.389	0.198	0.345	0.936	0.344
CBS OH_N20:0	1.381	1.768	1.328	1.483	0.985	2.599	1.432	1.936	1.922	1.449	1.210	1.670	1.633	1.221	1.326	0.909	3.319	2.109	2.873	1.498	0.783	1.166	0.798	2.391	1.041	2.068	3.062	1.003	1.344	1.280	3.164	0.950
CBS N22:1	0.778	1.266	0.686	0.465	0.253	1.695	0.855	1.093	1.405	0.665	0.453	1.139	0.819	0.843	0.461	0.424	2.082	0.944	2.477	1.057	0.390	0.500	0.365	1.642	0.468	0.971	1.468	0.698	0.739	0.856	1.930	0.518
CBS N22:0	1.080	1.901	1.051	1.636	0.536	2.780	1.768	1.942	1.484	1.305	1.082	1.640	1.927	1.257	0.958	1.219	2.183	1.789	3.051	1.328	0.711	0.860	0.671	2.454	0.764	1.127	4.361	1.661	0.915	1.525	2.268	1.062
CBS OH_N21:0	0.566	0.757	0.560	0.585	0.031	1.117	0.320	0.643	1.125	0.551	0.223	0.780	0.599	0.719	0.405	0.318	1.202	0.751	1.522	0.654	0.358	0.467	0.280	0.808	0.313	0.555	1.121	0.736	0.523	0.647	1.346	0.344
CBS OH_N22:2	0.201	0.667	0.203	0.101	0.157	0.670	0.288	0.422	0.557	0.361	0.171	0.447	0.519	0.235	0.288	0.130	0.755	0.487	0.848	0.388	0.145	0.293	0.235	0.559	0.275	0.214	0.780	0.407	0.190	0.425	0.543	0.527
CBS N23:0/ OH_N22:1	1.447	2.340	1.284	1.172	0.712	2.386	1.637	1.923	2.347	1.742	0.901	1.773	2.725	1.434	1.455	1.049	2.687	2.171	3.528	2.280	0.874	1.164	0.820	2.119	1.150	1.515	3.782	2.261	1.080	1.810	2.600	1.578
CBS OH_N22:0	5.806	10.792	6.170	9.626	3.173	10.789	6.063	8.191	6.729	6.331	4.752	6.072	8.354	4.773	6.164	4.390	9.141	9.660	11.979	8.097	3.729	4.512	2.966	9.017	4.117	4.686	15.975	7.477	3.291	6.505	9.104	6.283
CBS N24:1	4.702	13.238	6.154	11.349	2.781	12.572	7.647	10.854	7.554	8.823	4.373	6.733	10.393	5.228	5.804	5.096	7.954	10.479	15.972	8.794	3.891	5.071	3.679	10.835	3.788	3.729	21.357	9.300	3.274	9.945	8.162	7.310
CBS N24:0	1.625	2.837	1.801	4.146	0.312	3.608	2.120	2.735	2.136	2.538	1.234	1.689	3.716	1.341	1.818	2.059	2.373	2.865	4.287	2.371	1.192	0.917	0.850	3.050	1.301	1.212	7.137	3.140	0.742	2.536	2.372	2.439
CBS OH_N23:0	2.062	3.993	1.890	3.850	0.681	3.637	2.204	3.657	2.572	2.755	1.789	2.638	4.146	2.104	2.316	2.016	2.535	3.582	5.132	3.085	1.672	1.245	0.804	3.249	1.751	1.736	7.258	3.613	1.658	2.947	2.983	2.903
CBS N25:1/OH_N24:2	0.540	1.350	0.688	0.614	0.556	0.780	0.873	1.574	1.032	1.068	0.592	1.454	1.979	0.450	0.447	0.756	1.050	0.997	1.477	1.786	0.328	0.302	0.661	1.875	0.538	0.971	1.967	1.826	0.296	1.084	1.210	1.604
CBS OH_N24:1/N25:0	2.876	5.932	2.630	5.538	1.329	5.167	3.563	3.854	3.003	4.483	1.643	2.991	4.296	2.611	3.172	1.828	3.308	4.752	7.304	4.373	2.099	2.071	1.787	3.993	2.003	1.837	8.061	3.976	1.775	3.367	3.589	2.903
CBS OH_N24:0	2.373	6.476	3.057	7.606	1.181	5.395	3.525	4.447	3.304	4.378	2.205	2.415	5.255	2.734	3.440	2.657	3.386	5.359	7.763	3.739	1.603	1.841	1.067	3.612	1.755	1.242	10.036	4.524	1.134	4.244	3.919	3.446
PC A16:0-16:0	0.737	0.469	1.364	0.508	1.121	0.409	0.813	0.535	0.713	0.727	0.614	0.649	0.924	0.726	0.964	0.782	1.538	0.350	0.600	1.320	0.547	0.278	0.593	0.538	0.755	0.942	0.289	1.197	0.000	0.668	1.189	0.941
PC D16:1-16:0/D14:1-18:0	1.486	0.762	1.708	0.381	1.342	1.847	1.422	0.845	1.918	1.133	1.398	0.820	0.248	1.099	0.549	1.481	1.995	0.221	1.373	1.102	0.779	0.217	1.063	1.479	0.718	1.329	0.559	0.949	1.155	0.952	1.675	0.959
PC D16:0-16:0	30.102	24.075	29.461	15.501	26.271	35.833	32.659	25.344	25.726	26.180	29.541	23.814	21.248	21.796	20.447	31.520	29.830	17.656	19.378	26.205	26.824	13.632	23.290	31.288	22.336	21.197	16.194	23.126	23.657	19.068	28.431	24.785
PC P16:0-18:0/P18:0-16:0/A18:1-16:0/A16:0-18:1	1.461	1.263	1.410	1.153	0.971	2.105	1.004	1.108	1.613	1.350	1.272	0.981	0.569	1.365	1.111	1.210	0.907	0.674	1.655	1.144	1.298	0.404	0.908	1.487	1.070	1.369	0.888	1.038	1.094	1.078	1.522	1.275
PC A16:0-18:0	3.093	1.598	2.473	1.026	2.752	3.556	2.720	1.932	2.397	2.006	2.665	1.839	1.773	2.446	1.964	2.561	3.364	1.786	1.546	1.816	2.527	0.912	2.202	3.244	2.077	1.853	1.408	1.970	2.667	1.822	2.646	2.309

	4.0	2.9	3.2	3.1	2.7	5.2	3.2	3.2	3.0	3.0	3.5	1.8	2.8	3.1	3.1	2.7	3.2	2.4	3.7	3.0	2.2	1.6	1.8	3.0	2.7	2.4	3.1	2.9	2.0	2.6	3.3	3.3
PC D16:0-18:2	61	24	07	08	14	65	06	34	12	34	31	86	22	05	73	21	28	59	50	98	77	13	06	88	72	29	65	86	06	86	98	36
						108																										
	78.	80.	69.	68.	58.	.30	86.	66.	74.	75.	72.	74.	73.	68.	59.	74.	80.	66.	81.	65.	68.	39.	56.	77.	64.	60.	67.	62.	61.	66.	77.	65.
PC D16:0-18:1	489	157	538	290	932	6	027	447	316	097	929	984	095	787	699	188	120	156	019	730	173	496	502	217	955	878	510	709	424	916	418	696
	5.0	5.6	5.4	4.9	4.2	7.6	6.9	5.1	3.9	5.3	4.6	4.6	5.0	4.0	3.6	5.5	4.9	5.1	4.4	5.2	4.8	3.2	4.4	5.4	5.3	3.3	4.2	4.0	4.5	3.9	4.2	4.5
PC D16:0-18:0	77	38	52	99	07	59	25	94	94	08	95	92	09	42	70	38	61	44	63	37	24	16	99	95	60	41	14	63	59	58	80	38
PC D18:2-18:2/D16:0-	11.	9.9	11.	6.6	8.8	17.	10.	9.1	9.5	10.	13.	8.6	10.	9.3	8.9	12.	9.9	7.1	7.2	10.	11.	5.5	8.5	11.	8.7	7.9	6.9	10.	11.	8.3	9.9	10.
20:4	790	75	935	20	73	010	384	96	30	620	757	68	554	91	01	134	33	89	09	968	149	46	16	312	27	51	47	029	342	23	19	321
PC D18:1-18:2/D16:0-	2.9	2.3	2.6	2.8	2.2	5.6	2.4	2.6	2.2	2.8	2.9	1.7	2.1	2.5	2.3	2.8	2.6	2.0	2.7	2.7	2.3	1.5	2.2	2.7	2.5	1.6	2.8	2.5	2.6	2.3	2.3	2.4
20:3	65	51	76	50	90	33	30	66	78	82	42	24	81	33	86	62	97	07	30	59	79	21	88	78	88	61	30	37	37	51	28	10
PC D18:0-18:2/D18:1-	5.7	7.0	5.6	6.2	4.5	9.8	6.5	5.4	6.0	5.9	5.2	5.5	5.4	5.8	4.6	5.1	6.4	5.6	8.0	5.8	4.9	3.4	3.8	5.7	5.0	4.9	5.8	4.6	5.2	5.2	4.9	4.8
18:1	59	20	05	10	36	02	09	18	02	85	93	36	38	46	79	90	50	88	93	09	09	61	74	70	26	11	12	83	78	97	79	97
	15.	19.	14.	20.	11.	29.	16.	17.	16.	18.	12.	14.	18.	16.	12.	15.	16.	17.	25.	16.	12.	7.8	9.7	17.	12.	13.	20.	13.	10.	16.	14.	13.
PC D18:0-18:1	574	347	814	241	899	038	065	188	787	985	633	873	046	543	516	215	113	711	056	325	658	72	23	236	644	006	067	335	031	736	208	169
PC D16:0-22:6/D18:2-	8.0	8.2	5.3	8.8	5.8	9.8	11.	6.2	6.8	6.9	7.7	6.4	7.6	6.1	6.4	7.3	9.4	7.2	7.5	5.7	6.4	3.9	5.7	8.4	7.0	4.7	7.0	6.5	5.0	6.1	8.6	6.3
20:4	63	76	86	86	59	40	069	61	83	98	42	60	13	33	61	01	05	64	97	53	98	49	30	78	13	96	42	25	52	56	50	48
PC D18:1-20:4/D16:0-	2.5	3.0	3.0	1.5	2.0	4.5	1.9	1.6	2.6	2.4	3.8	1.7	1.9	2.9	2.3	2.4	2.8	1.5	1.4	2.8	3.5	1.7	3.2	2.5	2.0	1.3	2.5	2.4	2.8	2.7	2.6	3.2
22:5	63	17	83	32	19	17	22	82	68	54	82	78	63	39	34	96	29	91	62	99	54	25	43	77	18	18	62	42	67	19	22	05
PC D18:2-20:2/D18:0-	10.	11.	11.	10.	9.8	20.	10.	10.	9.6	11.	12.	7.9	10.	11.	9.1	10.	9.9	8.8	11.	12.	10.	6.0	7.7	10.	8.9	7.0	10.	10.	8.8	10.	8.1	10.
20:4	851	699	506	678	94	852	319	860	84	453	727	75	332	529	86	613	31	68	235	263	620	19	15	519	30	11	139	858	54	057	95	066
	0.9	1.7	1.1	2.1	1.0	2.1	1.0	1.4	1.3	1.4	0.7	1.0	1.5	1.4	0.8	1.1	1.1	1.0	1.6	1.2	0.7	0.5	0.6	0.7	0.8	0.6	1.1	1.1	0.6	1.1	1.2	0.8
PC D18:0-20:3	96	31	87	00	36	74	43	22	63	59	17	51	48	50	14	89	35	81	62	74	03	56	95	91	66	65	89	64	55	39	38	42
PC D18:0-20:2/P18:2-	0.8	1.8	1.3	2.0	0.9	1.9	1.0	1.2	1.5	1.2	0.7	1.5	1.5	1.1	0.6	0.8	1.0	1.3	1.8	0.9	0.7	0.3	0.5	0.9	0.9	0.8	1.1	0.6	1.2	1.1	0.9	0.7
22:6	39	66	39	46	68	41	45	87	85	61	05	95	86	23	84	84	67	52	93	58	03	98	36	77	09	88	75	49	72	16	72	22
PC D18:0-20:1/P18:1-	0.2	0.6	0.3	0.9	0.2	0.6	0.1	0.4	0.5	0.1	0.1	0.1	0.0	0.4	0.3	0.1	0.9	0.2	0.5	0.1	0.1	0.4	0.1	0.1	0.2	0.0	0.4	0.1	0.0	0.2	0.0	0.0
22:6	52	63	60	19	29	28	81	91	10	93	49	23	99	09	93	34	43	27	00	98	34	45	63	54	82	00	38	74	00	63	44	97
PC D18:1-22:6/D18:2-	2.3	1.6	1.3	2.8	1.2	3.5	2.2	1.1	1.3	2.2	2.0	0.9	1.5	2.1	2.4	1.7	2.3	2.3	2.9	2.0	1.5	1.4	1.9	2.5	2.0	1.4	2.8	2.1	0.9	2.1	2.1	2.4
22:5	45	38	58	18	58	52	54	10	65	35	28	59	92	48	61	99	48	85	83	39	80	90	78	49	43	02	42	12	79	64	11	92
	3.4	4.2	2.5	5.7	3.1	4.7	4.0	2.9	3.0	3.5	3.3	2.8	3.0	3.1	2.6	2.8	3.3	3.9	4.4	2.8	2.5	1.7	2.4	3.5	3.2	1.9	3.8	2.9	1.3	2.8	3.0	2.8
PC D18:0-22:6	14	65	70	24	19	60	40	40	58	62	49	24	38	95	03	67	75	06	62	67	08	72	92	37	96	17	25	11	50	92	23	90
PC D18:0-22:4/D20:0-	0.5	0.7	0.6	0.2	0.8	0.3	0.2	0.6	0.6	0.5	0.3	0.0	0.0	0.6	0.0	0.3	0.8	0.0	0.1	0.5	0.2	0.0	0.6	0.3	0.7	0.7	0.0	0.2	1.8	0.3	0.0	0.1
20:4/D20:2-20:2	68	11	37	18	52	60	85	43	66	40	90	00	00	60	85	84	68	95	98	78	30	00	77	47	23	38	00	91	67	24	00	57
	7.5	9.5	10.	5.7	5.2	14.	8.1	7.1	9.6	10.	6.5	6.8	7.2	6.9	3.9	8.7	7.3	8.6	7.1	8.6	7.2	5.0	6.7	6.8	8.0	7.1	7.7	5.9	7.0	7.3	6.6	9.8
PI 18:0-20:4	25	95	883	67	07	463	27	62	03	223	87	58	68	29	43	57	85	25	35	64	47	37	52	80	95	46	10	00	12	48	61	09
	0.1	0.4	0.1	0.2	0.1	0.4	0.2	0.2	0.3	0.2	0.1	0.2	0.2	0.1	0.1	0.1	0.3	0.3	0.4	0.2	0.2	0.1	0.1	0.2	0.1	0.1	0.2	0.1	0.1	0.2	0.2	0.2
PI 16:0-18:1	78	28	82	22	49	08	77	24	01	28	14	08	77	80	08	69	08	59	71	00	02	53	47	16	82	84	72	23	04	39	93	05
	0.0	0.1	0.0	0.0	0.3	0.1	0.0	0.1	0.1	0.0	0.0	0.0	0.0	0.1	0.0	0.0	0.1	0.0	0.1	0.1	0.0	0.0	0.0	0.0	0.0	0.0	0.0	0.0	0.0	0.0	0.0	0.0
PI 16:0-18:0	75	55	56	65	04	95	93	07	22	74	40	79	69	50	31	64	09	72	59	31	80	41	61	67	55	63	72	48	46	93	88	86
	1.4	1.6	1.7	1.0	0.9	2.4	1.8	1.2	1.7	1.6	1.3	1.3	1.3	1.1	0.7	1.6	1.4	1.4	1.3	1.4	1.0	0.9	1.2	1.3	1.4	1.3	1.1	1.0	1.1	1.4	1.5	1.7
PI 16:0-20:4	65	25	27	38	36	29	07	31	21	80	24	10	54	54	59	58	90	99	17	06	99	59	82	76	88	21	79	04	22	28	07	73
	0.1	0.4	0.2	0.1	0.1	0.4	0.3	0.2	0.4	0.3	0.1	0.2	0.3	0.2	0.1	0.2	0.3	0.3	0.4	0.2	0.1	0.1	0.1	0.2	0.1	0.1	0.3	0.1	0.1	0.1	0.2	0.2
PI 18:0-18:1	84	09	34	89	03	70	20	66	08	14	42	31	12	24	23	15	02	26	84	57	47	74	51	38	68	89	63	50	43	87	85	58
	0.2	0.5	0.2	0.3	0.3	0.3	0.5	0.2	0.3	0.3	0.2	0.2	0.3	0.2	0.1	0.2	0.3	0.4	0.3	0.2	0.1	0.2	0.2	0.2	0.3	0.1	0.3	0.1	0.1	0.2	0.3	0.2
PI 18:2-20:4/16:0-22:6	79	10	14	20	67	92	12	61	03	08	51	56	17	77	98	30	81	49	75	56	79	10	23	81	29	91	97	64	76	57	06	90
	1.2	1.1	1.4	0.8	0.6	2.3	1.4	1.0	1.3	1.4	1.2	0.9	0.8	1.0	0.7	1.3	1.2	1.2	0.8	1.2	0.9	0.7	1.0	1.2	1.2	0.9	1.1	1.0	0.9	0.9	1.0	1.5
PI 18:1-20:4	29	41	74	84	37	75	27	54	42	24	85	16	79	60	58	70	23	70	81	33	69	43	46	17	76	91	78	21	76	80	83	53
	7.5	9.5	10.	5.7	5.2	14.	8.1	7.1	9.6	10.	6.5	6.8	7.2	6.9	3.9	8.7	7.3	8.6	7.1	8.6	7.2	5.0	6.7	6.8	8.0	7.1	7.7	5.9	7.0	7.3	6.6	9.8
PI 18:0-20:4	25	95	883	67	07	463	27	62	03	223	87	58	68	29	43	57	85	25	35	64	47	37	52	80	95	46	10	00	12	48	61	09
	0.1	0.1	0.1	0.1	0.0	0.2	0.1	0.1	0.2	0.2	0.1	0.1	0.1	0.1	0.2	0.2	0.1	0.2	0.0	0.1	0.1	0.0	0.1	0.0	0.1	0.1	0.2	0.0	0.0	0.1	0.1	0.1
PI 18:0-20:3	70	94	57	60	83	43	71	80	30	53	31	57	91	49	05	13	76	03	40	67	51	87	61	73	41	92	39	84	97	28	43	80
	0.2	0.3	0.3	0.3	0.3	0.4	0.2	0.2	0.2	0.3	0.2	0.2	0.3	0.5	0.4	0.2	0.2	0.2	0.1	0.3	0.2	0.4	0.2	0.2	0.3	0.2	0.2	0.2	0.3	0.2	0.2	0.3
PI 18:0-20:1	98	23	59	60	60	07	38	35	62	02	45	92	03	86	74	58	36	86	64	25	66	06	37	31	17	45	56	36	85	42	58	09
	0.0	0.0	0.0	0.0	0.0	0.0	0.0	0.0	0.0	0.0	0.0	0.0	0.0	0.0	0.0	0.0	0.0	0.0	0.1	0.0	0.0	0.0	0.0	0.0	0.0	0.0	0.0	0.0	0.0	0.0	0.0	0.0
PI P18:0-22:4	53	36	44	28	24	89	65	46	41	45	37	17	59	35	29	17	28	82	34	29	55	41	36	42	48	28	46	18	25	48	45	67
	0.0	0.0	0.0	0.0	0.0	0.0	0.0	0.0	0.0	0.0	0.0	0.0	0.0	0.0	0.0	0.0	0.0	0.0	0.0	0.0	0.0	0.0	0.0	0.0	0.0	0.0	0.0	0.0	0.0	0.0	0.0	0.0
PI A18:0-22:4	35	47	66	55	66	92	58	58	81	51	24	67	54	62	48	65	56	50	08	57	81	62	36	56	56	62	38	35	64	47	55	63
	0.0	0.0	0.0	0.0	0.0	0.1	0.0	0.0	0.0	0.0	0.0	0.0	0.0	0.0	0.0	0.0	0.0	0.0	0.0	0.0	0.1	0.0	0.0	0.0	0.0	0.0	0.0	0.0	0.0	0.0	0.0	0.0
PI A18:0-22:3	49	73	84	60	46	23	60	65	68	39	44	57	80	28	20	42	47	59	65	48	04	59	41	47	63	55	41	50	57	62	27	71
	0.2	0.6	0.2	0.4	0.6	0.3	0.4	0.2	0.3	0.3	0.2	0.3	0.3	0.3	0.1	0.2	0.3	0.5	0.4	0.2	0.2	0.2	0.2	0.2	0.2	0.2	0.5	0.1	0.1	0.2	0.2	0.2
PI 18:0-22:6	76	81	60	57	49	97	57	56	42	79	26	13	28	84	58	07	55	70	64	76	33	26	03	43	77	18	20	97	18	81	80	33
	0.2	0.2	0.2	0.1	0.0	0.2	0.1	0.1	0.1	0.2	0.1	0.2	0.1	0.1	0.1	0.1	0.1	0.1	0.0	0.1	0.0	0.1	0.1	0.1	0.2	0.1	0.2	0.1	0.1	0.1	0.1	0.1
PI 18:0-22:4	19	18	01	20	67	24	58	89	71	53	86	31	83	49	47	85	56	93	87	45	89	10	63	49	26	81	05	56	32	49	59	84
	0.2	0.4	0.5	0.2	0.1	0.4	0.4	0.3	0.2	0.5	0.2	0.3	0.4	0.2	0.1	0.3	0.3	0.4	0.3	0.3	0.2	0.3	0.3	0.3	0.3	0.3	0.3	0.2	0.3	0.2	0.2	0.5
PI 22:6-22:8	70	55	13	11	78	42	31	64	78	79	75	11	15	60	68	06	42	32	20	75	13	53	73	93	82	40	30	63	45	90	99	33
	0.2	0.2	0.2	0.2	0.3	0.4	0.2	0.2	0.2	0.3	0.1	0.2	0.2	0.4	0.4	0.2	0.1	0.2	0.0	0.2	0.1	0.2	0.3	0.2	0.2	0.2	0.3	0.1	0.3	0.2	0.1	0.3
PI 22:8-24:7	28	56	87	94	61	46	49	20	21	23	75	13	46	43	07	24	61	86	85	64	97	94	13	47	95	24	09	76	30	07	86	44
						124																										
	93.	65.	63.	48.	58.	.80	71.	82.	57.	61.	74.	61.	72.	76.	89.	65.	94.	50.	85.	73.	78.	41.	61.	79.	45.	49.	66.	74.	54.	75.	67.	60.
PS 18:0-22:6	905	938	218	156	724	6	800	145	566	012	748	038	413	642	384	964	847	973	661	101	584	192	046	303	986	161	641	535	601	225	240	334
	3.1	2.4	2.3	2.7	3.1	3.5	1.8	3.2	2.4	2.2	2.4	2.3	4.0	4.0	2.8	1.8	3.3	2.1	2.9	2.9	2.1	1.1	1.7	2.2	2.3	2.5	2.8	3.5	2.1	3.5	3.0	2.5
PS 16:0-18:1	37	80	09	30	08	18	34	52	53	74	97	76	46	89	94	05	62	01	33	76	64	10	00	86	40	03	31	24	31	58	61	92

PS 16:0-18:0	0.166	0.102	0.093	0.042	0.140	0.093	0.081	0.112	0.051	0.050	0.025	0.084	0.185	0.180	0.065	0.058	0.145	0.059	0.081	0.130	0.175	0.072	0.065	0.083	0.076	0.079	0.108	0.073	0.093	0.115	0.081	0.087
PS P18:0-18:0	0.188	0.306	0.142	0.404	0.170	0.373	0.154	0.234	0.188	0.170	0.115	0.230	0.366	0.332	0.217	0.057	0.432	0.317	0.602	0.160	0.143	0.100	0.043	0.188	0.125	0.167	0.502	0.178	0.100	0.358	0.342	0.155
PS 18:2-18:2	0.230	0.168	0.195	0.124	0.440	0.226	0.106	0.159	0.170	0.152	0.289	0.147	0.452	0.485	0.150	0.095	0.237	0.166	0.023	0.222	0.118	0.066	0.097	0.168	0.212	0.182	0.205	0.267	0.224	0.248	0.218	0.206
PS 18:1-18:2	0.218	0.182	0.132	0.204	0.150	0.195	0.152	0.215	0.152	0.175	0.153	0.178	0.245	0.276	0.174	0.058	0.214	0.144	0.207	0.129	0.081	0.065	0.046	0.092	0.169	0.186	0.233	0.172	0.122	0.146	0.158	0.149
PS 18:0-18:2	8.362	9.966	5.490	12.375	5.361	12.766	6.745	9.290	7.578	7.670	5.876	8.482	10.162	10.327	9.884	4.830	10.208	8.341	18.212	6.238	7.056	3.944	5.211	6.723	5.640	6.412	11.937	7.108	4.577	10.310	8.080	5.997
PS 18:0-18:1	31.145	42.194	20.235	46.846	20.381	59.816	25.935	37.866	27.510	25.532	19.260	26.504	41.046	35.902	40.352	16.833	49.192	34.940	91.527	30.109	24.469	18.437	16.277	29.455	14.510	17.881	56.664	26.368	15.341	47.061	32.833	21.535
PS 18:0-18:0	0.602	0.888	0.346	0.977	1.309	0.822	0.431	0.791	0.513	0.497	0.354	0.530	0.956	0.618	0.729	0.363	1.076	0.699	2.572	0.582	0.479	0.273	0.260	0.472	0.366	0.313	0.908	0.602	0.196	1.053	0.819	0.495
PS P18:0-20:0	0.212	0.235	0.120	0.499	0.096	0.329	0.150	0.197	0.173	0.154	0.068	0.202	0.294	0.247	0.208	0.046	0.412	0.201	1.021	0.082	0.065	0.100	0.056	0.152	0.080	0.115	0.451	0.131	0.123	0.305	0.235	0.139
PS 18:2-20:4	0.795	0.349	0.362	0.247	4.276	0.697	0.349	0.495	0.330	0.286	0.651	0.409	0.883	2.688	0.658	0.336	0.598	0.289	0.699	0.877	0.873	0.228	0.360	0.518	0.480	0.367	0.389	0.680	0.389	0.445	0.495	0.453
PS 18:1-20:4	0.721	0.724	0.423	0.795	0.751	1.233	0.527	0.734	0.511	0.591	0.737	0.539	0.927	1.095	0.926	0.367	0.976	0.772	1.271	0.592	0.775	0.304	0.407	0.583	0.543	0.491	1.120	0.810	0.381	0.946	0.752	0.669
PS 18:0-20:4	6.752	9.525	4.395	7.075	4.052	13.282	5.694	8.066	5.236	5.595	5.279	5.104	9.102	7.753	9.153	4.028	9.426	6.922	13.867	6.254	5.898	3.666	4.040	6.050	3.370	3.805	11.141	6.473	3.748	8.711	6.537	5.167
PS 18:0-20:3	1.396	1.559	0.848	1.614	0.836	2.375	1.104	1.516	1.190	1.043	0.919	1.000	1.500	1.349	1.322	0.686	2.126	1.199	2.896	0.888	0.965	0.646	0.579	1.151	0.564	0.823	2.005	1.015	0.669	1.403	1.397	0.898
PS 18:0-20:2	0.925	1.434	0.683	2.396	0.688	2.326	0.931	1.155	1.020	1.020	0.615	0.973	1.357	1.300	1.480	0.567	1.667	1.283	4.070	0.872	0.783	0.602	0.465	1.070	0.477	0.708	2.032	0.778	0.530	1.543	1.112	0.692
PS 18:0-20:1	2.125	2.939	1.377	6.656	1.164	4.643	1.910	2.542	1.775	2.112	1.447	1.852	2.964	2.502	2.956	1.182	3.280	2.930	9.456	1.828	1.571	1.336	0.957	1.926	0.919	1.124	4.783	1.630	0.958	3.568	2.077	1.367
PS 18:0-20:0	0.210	0.273	0.170	0.313	0.229	0.339	0.157	0.314	0.141	0.213	0.148	0.183	0.256	0.210	0.145	0.135	0.221	0.153	0.427	0.129	0.284	0.081	0.066	0.153	0.127	0.120	0.245	0.241	0.183	0.227	0.138	0.163
PS P18:0-22:5	0.195	0.109	0.100	0.155	0.130	0.178	0.087	0.084	0.111	0.080	0.065	0.141	0.142	0.146	0.025	0.036	0.170	0.091	0.361	0.044	0.059	0.036	0.086	0.108	0.090	0.109	0.039	0.092	0.091	0.117	0.139	0.094
PS 18:1-22:6	1.932	1.019	0.865	1.009	1.038	2.546	1.365	1.346	0.911	1.067	1.470	0.866	1.306	1.423	2.244	1.069	2.244	1.151	1.714	1.440	1.332	0.874	1.285	1.790	0.961	0.828	1.576	1.706	0.726	1.727	1.575	1.296
PS 18:0-22:6	93.905	65.938	63.218	48.156	58.724	124.806	71.800	82.145	57.566	61.012	74.748	61.038	72.413	76.642	89.384	65.964	94.847	50.973	85.661	73.101	78.584	41.192	61.046	79.303	45.986	49.161	66.641	74.535	54.601	75.225	67.240	60.334
PS 18:0-22:5	3.732	2.353	2.344	2.227	2.748	5.095	2.480	3.175	1.873	2.365	3.101	2.300	3.252	3.281	3.166	2.560	3.210	2.152	2.557	2.977	3.743	1.560	2.175	3.002	1.593	1.843	2.982	3.264	2.390	3.147	2.659	2.532
PS 20:0-20:4/18:0-22:4	6.605	6.670	5.104	6.656	4.292	10.410	3.876	6.977	4.049	5.917	4.952	4.783	6.973	7.955	6.273	4.440	5.500	5.402	12.624	6.369	4.783	2.724	4.372	4.923	3.753	3.950	7.982	5.939	3.232	7.887	3.809	4.997
PS 20:0-20:3/18:0-22:3	0.195	0.219	0.204	0.354	0.179	0.471	0.163	0.338	0.191	0.240	0.180	0.191	0.448	0.351	0.396	0.135	0.375	0.243	0.762	0.296	0.183	0.106	0.136	0.215	0.101	0.170	0.407	0.209	0.172	0.450	0.268	0.147
PS 20:0-20:2/18:0-22:2	0.514	1.001	0.466	1.251	0.280	1.653	0.583	0.830	0.521	0.704	0.401	0.546	0.845	0.635	0.932	0.419	0.734	0.733	1.895	0.657	0.483	0.421	0.280	0.537	0.247	0.263	1.187	0.476	0.291	0.912	0.435	0.459
PS 20:0-20:1/18:0-22:1	0.395	0.799	0.363	1.437	0.283	1.290	0.568	0.725	0.359	0.614	0.473	0.455	1.087	0.545	0.860	0.446	0.526	0.686	1.328	0.573	0.363	0.317	0.237	0.448	0.285	0.193	1.442	0.592	0.177	0.990	0.381	0.521
PS P20:0-22:5	0.186	0.161	0.161	0.150	0.192	0.236	0.168	0.249	0.133	0.117	0.121	0.188	0.168	0.183	0.149	0.116	0.214	0.122	0.236	0.121	0.277	0.119	0.083	0.141	0.146	0.116	0.133	0.150	0.141	0.166	0.229	0.109
PS P20:0-22:4	0.700	0.631	0.351	0.423	0.781	0.810	0.426	0.865	0.403	0.272	0.377	0.555	0.791	0.638	0.625	0.408	0.677	0.227	1.716	0.380	1.085	0.308	0.440	0.573	0.341	0.332	0.351	0.377	0.536	0.583	0.372	0.283
PS P20:0-22:2	0.164	0.166	0.083	0.108	0.048	0.315	0.118	0.102	0.053	0.115	0.199	0.058	0.115	0.121	0.264	0.104	0.198	0.134	0.270	0.094	0.087	0.122	0.121	0.112	0.113	0.051	0.152	0.226	0.076	0.227	0.118	0.112
PS 20:3-22:6/P20:0-22:1	0.087	0.230	0.113	0.211	0.139	0.240	0.127	0.202	0.076	0.148	0.101	0.141	0.213	0.123	0.199	0.056	0.144	0.172	0.357	0.099	0.164	0.037	0.025	0.110	0.054	0.036	0.248	0.137	0.044	0.238	0.092	0.106
PS 20:2-22:6	0.166	0.277	0.105	0.442	0.103	0.391	0.183	0.206	0.080	0.209	0.192	0.154	0.460	0.168	0.321	0.160	0.183	0.226	0.146	0.148	0.110	0.072	0.083	0.132	0.123	0.040	0.541	0.291	0.051	0.458	0.153	0.206
PS 20:1-22:6	0.119	0.185	0.067	0.277	0.030	0.258	0.089	0.096	0.041	0.125	0.078	0.061	0.161	0.127	0.178	0.078	0.173	0.173	0.051	0.040	0.068	0.078	0.070	0.079	0.069	0.046	0.158	0.099	0.030	0.173	0.070	0.112
PS 20:0-22:6	0.183	0.157	0.088	0.239	0.125	0.169	0.111	0.174	0.069	0.138	0.118	0.107	0.141	0.167	0.108	0.079	0.198	0.188	0.547	0.130	0.080	0.090	0.058	0.164	0.083	0.045	0.240	0.104	0.039	0.237	0.134	0.110
PS 20:0-22:4	0.353	0.486	0.331	0.465	0.411	0.486	0.340	0.502	0.303	0.334	0.258	0.358	0.567	0.404	0.247	0.275	0.386	0.249	0.989	0.333	0.358	0.142	0.252	0.297	0.211	0.269	0.440	0.221	0.359	0.421	0.275	0.243
PS 20:0-22:2	0.177	0.453	0.183	0.655	0.106	0.533	0.210	0.317	0.121	0.293	0.162	0.176	0.545	0.271	0.302	0.176	0.201	0.256	0.411	0.203	0.161	0.126	0.075	0.175	0.103	0.059	0.633	0.219	0.091	0.536	0.162	0.203
PS 20:0-22:1	0.100	0.266	0.102	0.825	0.048	0.335	0.193	0.203	0.079	0.209	0.154	0.123	0.503	0.074	0.160	0.180	0.091	0.216	0.475	0.177	0.131	0.054	0.055	0.116	0.074	0.017	0.583	0.259	0.036	0.401	0.076	0.170
PS 22:6-22:6	0.493	0.349	0.161	1.096	0.085	0.844	0.320	0.225	0.160	0.373	0.653	0.097	0.280	0.386	0.823	0.187	0.703	0.827	1.795	0.255	0.509	0.246	0.276	0.471	0.242	0.114	1.500	0.593	0.071	0.714	0.468	0.456
PS 22:6-22:5	0.083	0.128	0.055	0.198	0.048	0.154	0.098	0.072	0.028	0.094	0.147	0.042	0.096	0.032	0.090	0.061	0.081	0.127	0.000	0.030	0.197	0.045	0.047	0.122	0.054	0.035	0.314	0.117	0.033	0.138	0.079	0.088

	1.1	0.5	0.4	1.3	0.3	2.0	0.5	0.6	0.3	0.9	1.2	0.3	0.6	0.9	1.5	0.6	0.7	0.9	1.6	0.9	1.0	0.4	0.6	0.8	0.4	0.3	1.6	1.2	0.2	1.2	0.4	0.8
PS 22:6-22:4	32	83	91	53	52	42	11	17	59	54	70	59	96	89	84	15	65	66	35	27	07	29	34	19	95	71	72	63	45	55	73	71
	0.3	1.4	1.0	0.5	0.5	0.3	0.6		1.8	0.6	0.9	0.6	0.9	0.4	0.4	0.9	0.7	1.3	0.4		0.2	1.0	0.5	0.5	1.3	0.6	1.0	0.7	0.4	0.4	0.5	0.4
LYSOPC P14:1	74	22	34	03	83	36	01		86	73	81	83	18	54	77	16	61	04	90		73	22	04	49	17	51	12	93	83	95	32	95
	0.0	0.0	0.1	0.0	0.2	0.0	0.0		0.4	0.0	0.1	0.1	0.1	0.1	0.0	0.1	0.2	0.2	0.0		0.0	0.2	0.1	0.0	0.1	0.0	0.1	0.1	0.1	0.0	0.0	0.0
LYSOPC P14:0	63	63	22	80	54	49	33		16	65	69	29	47	48	39	28	79	83	49		50	45	53	46	68	35	13	03	03	33	39	57
	0.4	0.6	0.3	0.3	0.4	0.1	0.3		0.4	0.4	0.4	0.3	0.3	0.1	0.1	0.3	0.3	0.2	0.2		0.2	0.4	0.3	0.2	0.3	0.3	0.4	0.2	0.2	0.4	0.2	0.3
LYSOPC 14:1	77	98	44	31	26	64	42		90	16	08	84	46	36	79	39	69	95	74		57	40	80	75	74	16	14	13	88	16	76	95
	0.9	3.5	2.7	1.4	3.1	0.9	1.0		4.5	1.2	2.2	1.7	2.7	2.1	1.0	1.8	1.4	2.4	1.4		1.1	1.3	0.9	1.2	2.3	2.2	2.9	1.7	1.4	1.5	1.5	1.1
LYSOPC P16:0	26	74	02	42	00	56	75		32	41	91	62	21	32	25	07	35	73	61		78	78	59	15	77	45	33	74	30	14	68	39
	0.8	4.1	3.0	2.3	3.4	1.6	1.2		3.8	1.6	2.4	2.0	3.2	2.1	1.6	2.3	1.4	3.0	1.6		1.7	3.5	1.0	1.7	2.6	2.8	2.8	2.2	2.4	1.5	1.9	1.3
LYSOPC 16:1	06	59	01	66	35	09	48		08	31	49	26	07	25	52	80	64	42	53		18	47	69	69	02	11	56	43	59	07	53	40
	1.2	1.3	1.5	1.1	1.9	1.0	1.1		1.6	1.1	1.2	1.4	1.2	1.3	0.8	1.1	1.3	1.3	1.0		1.1	1.0	1.0	0.8	1.1	1.1	1.6	0.9	1.4	1.4	1.2	1.2
LYSOPC 16:0	11	92	96	47	30	99	99		97	14	20	03	04	26	29	57	19	26	07		78	82	79	53	85	08	82	95	77	71	35	75
	0.0	0.2	0.1	0.4	0.1	0.0	0.0		0.2	0.0	0.0	0.1	0.1	0.0	0.0	0.0	0.1	0.1	0.1		0.0	0.1	0.0	0.0	0.1	0.0	0.2	0.0	0.0	0.0	0.0	0.0
LYSOPC P18:2	36	41	84	02	02	52	34		42	71	90	62	45	66	97	52	16	13	03		58	87	36	61	04	82	33	81	64	46	91	16
	0.6	3.2	1.8	1.6	2.3	0.7	1.0		2.1	0.7	1.2	1.5	1.9	1.6	1.3	1.4	1.2	2.1	1.1		0.9	1.1	0.6	1.1	1.6	1.5	2.4	0.9	1.5	0.9	1.2	1.1
LYSOPC P18:1	17	63	14	92	80	37	14		94	16	43	63	91	53	14	05	99	09	33		66	02	12	08	00	36	19	79	40	27	56	29
	0.3	1.7	1.0	0.8	1.9	0.6	0.4		1.5	0.4	0.8	0.6	1.0	1.2	0.5	0.8	0.5	1.1	0.6		0.5	0.5	0.3	0.6	0.8	0.8	1.4	0.7	0.8	0.5	0.6	0.5
LYSOPC P18:0	44	60	07	43	65	51	21		11	83	65	41	75	87	58	46	81	40	20		79	83	38	33	33	56	89	23	70	90	91	23
	0.8	1.3	1.0	0.7	1.0	0.6	0.9		0.8	0.5	0.6	1.0	0.7	0.5	0.5	0.8	0.8	0.6	0.6		0.7	0.7	0.5	0.6	0.6	0.6	1.4	0.4	0.5	0.6	0.6	0.7
LYSOPC 18:1	19	48	39	71	21	43	03		43	46	16	98	89	68	28	72	60	42	91		76	85	47	80	84	81	63	90	49	87	32	55
	0.4	0.5	0.5	0.3	0.7	0.4	0.3		0.5	0.3	0.4	0.4	0.4	0.7	0.3	0.3	0.5	0.3	0.3		0.4	0.3	0.3	0.3	0.3	0.2	0.7	0.2	0.3	0.4	0.4	0.4
LYSOPC 18:0	59	46	81	46	58	29	52		10	52	62	94	63	69	30	49	11	79	63		19	81	54	09	75	75	62	92	68	33	29	32
	0.4	1.5	1.2	0.6	0.8	0.4	0.6		1.1	0.2	0.9	0.5	0.9	0.7	0.8	0.8	0.5	0.9	0.8		0.5	0.5	0.2	0.6	0.9	0.5	1.0	0.6	0.6	0.4	0.4	0.6
LYSOPC P20:4	02	26	77	30	17	48	07		05	32	51	14	63	69	64	38	93	14	49		99	21	23	20	07	54	80	88	63	80	93	77
	0.6	0.5	0.7	0.2	0.2	0.4	0.4		0.3	0.1	0.3	0.3	0.3	0.3	0.6	0.5	0.3	0.2	0.5		0.3	0.2	0.3	0.3	0.3	0.2	0.5	0.2	0.1	0.2	0.3	0.4
LYSOPC 20:4	00	37	66	76	10	00	27		26	86	18	15	81	07	87	51	67	84	26		27	05	26	34	70	51	55	87	89	68	70	65
	0.2	1.3	0.7	0.9	0.5	0.4	0.3		0.6	0.2	0.5	0.4	0.7	0.3	0.5	0.4	0.5	0.9	0.3		0.2	0.4	0.2	0.4	0.6	0.3	1.3	0.2	0.2	0.2	0.3	0.5
LYSOPC 20:0	62	30	00	39	69	17	03		10	25	55	27	65	97	45	36	59	03	98		99	42	07	68	57	56	39	84	56	75	61	54
	0.2	1.3	0.7	0.9	0.5	0.4	0.3		0.6	0.2	0.5	0.4	0.7	0.4	0.5	0.4	0.5	0.9	0.4		0.3	0.4	0.2	0.4	0.6	0.3	1.3	0.2	0.2	0.2	0.3	0.5
LYSOPC P22:6	68	55	15	59	80	24	09		23	30	67	35	81	06	56	45	71	21	05		05	52	12	77	70	64	66	90	61	81	72	66
	0.3	0.4	0.2	0.5	0.0	0.2	0.2		0.2	0.1	0.3	0.2	0.2	0.2	0.2	0.3	0.3	0.2	0.2		0.2	0.1	0.1	0.2	0.2	0.1	0.7	0.1	0.1	0.2	0.2	0.3
LYSOPC 22:6	58	47	41	51	93	21	14		11	19	08	65	91	17	15	04	50	17	23		55	60	76	68	35	25	07	90	04	36	92	20
	0.0	0.0	0.0	0.0	0.0	0.0	0.0		0.0	0.0	0.0	0.0	0.0	0.0	0.0	0.0	0.0	0.0	0.0		0.0	0.0	0.0	0.0	0.0	0.0	0.0	0.0	0.0	0.0	0.0	0.0
CARN 14:0	47	36	28	22	72	42	27		37	23	19	34	30	47	46	21	50	36	36		30	27	19	53	14	33	40	25	34	43	49	26
	0.0	0.0	0.0	0.0	0.0	0.0	0.0		0.0	0.0	0.0	0.0	0.0	0.0	0.0	0.0	0.1	0.0	0.0		0.0	0.0	0.0	0.0	0.0	0.0	0.0	0.0	0.0	0.0	0.1	0.0
CARN 16:0	85	76	47	46	99	67	72		81	52	46	70	77	64	58	40	14	89	65		48	51	38	58	44	76	77	44	60	75	06	57
	0.0	0.0	0.0	0.0	0.0	0.0	0.0		0.0	0.0	0.0	0.0	0.0	0.0	0.0	0.0	0.0	0.0	0.0		0.0	0.0	0.0	0.0	0.0	0.0	0.0	0.0	0.0	0.0	0.0	0.0
CARN 18:2	20	11	23	18	19	39	13		09	10	11	12	21	14	08	16	21	21	14		05	12	09	11	12	28	18	13	11	10	18	09
	0.0	0.0	0.0	0.0	0.0	0.0	0.0		0.0	0.0	0.0	0.0	0.0	0.0	0.0	0.0	0.0	0.0	0.0		0.0	0.0	0.0	0.0	0.0	0.0	0.0	0.0	0.0	0.0	0.0	0.0
CARN 18:1	47	47	34	32	67	38	44		45	31	26	36	40	33	55	29	80	50	36		27	34	20	36	22	41	42	22	37	49	58	23
	0.0	0.0	0.0	0.0	0.0	0.0	0.0		0.0	0.0	0.0	0.0	0.0	0.0	0.0	0.0	0.0	0.0	0.0		0.0	0.0	0.0	0.0	0.0	0.0	0.0	0.0	0.0	0.0	0.0	0.0
CARN 20:2-OH	09	12	16	19	11	33	08		12	08	18	14	10	11	14	17	18	16	11		07	11	08	11	17	16	07	12	10	11	15	07
	0.0	0.0	0.0	0.0	0.0	0.0	0.0		0.0	0.0	0.0	0.0	0.0	0.0	0.0	0.0	0.0	0.0	0.0		0.0	0.0	0.0	0.0	0.0	0.0	0.0	0.0	0.0	0.0	0.0	0.0
CARN 24:0	12	17	20	14	13	39	10		17	11	20	18	15	20	18	21	17	22	14		08	11	13	11	23	19	11	16	11	13	17	11
	0.0	0.0	0.0	0.0	0.0	0.0	0.0		0.0	0.0	0.0	0.0	0.0	0.0	0.0	0.0	0.0	0.0	0.0		0.0	0.0	0.0	0.0	0.0	0.0	0.0	0.0	0.0	0.0	0.0	0.0
CARN 26:0-OH	12	10	21	07	09	31	11		15	10	16	11	14	14	15	16	19	15	10		05	10	08	11	14	15	11	14	12	10	14	10
	0.4	2.1	1.4	1.3	1.9	1.1	0.8		2.0	0.8	1.2	1.2	1.7	1.5	1.1	1.3	0.9	1.5	1.1		0.7	1.1	0.5	1.1	1.5	1.3	1.2	1.2	1.1	0.5	0.9	0.5
LYSOPE P16:1	41	69	10	45	58	30	67		71	62	14	30	32	66	37	72	38	60	27		99	05	69	30	13	96	98	27	70	91	58	99
	0.3	0.5	0.5	0.6	0.3	0.3	0.3		0.7	0.1	0.3	0.3	0.4	0.5	0.4	0.4	0.4	0.4	0.4		0.4	0.2	0.1	0.2	0.4	0.3	0.4	0.2	0.4	0.2	0.3	0.2
LYSOPE P16:0	52	97	69	96	86	29	27		70	74	23	75	13	41	46	76	89	13	36		33	96	75	76	60	49	71	63	84	73	87	51
	0.5	0.6	0.7	0.8	0.7	0.7	0.5		0.4	0.5	0.4	0.7	0.5	0.6	0.7	0.6	0.6	0.5	0.6		0.7	0.4	0.4	0.5	0.5	0.6	0.6	0.5	0.7	0.5	0.5	0.5
LYSOPE 16:1	54	58	68	28	20	00	49		87	29	90	05	61	22	12	33	27	93	69		68	72	85	87	62	18	48	54	52	28	57	53
	0.1	0.1	0.1	0.3	0.0	0.1	0.1		0.1	0.0	0.1	0.2	0.1	0.1	0.3	0.1	0.1	0.1	0.1		0.3	0.0	0.1	0.1	0.1	0.0	0.1	0.0	0.1	0.1	0.2	0.1
LYSOPE 16:0	84	47	53	23	79	83	19		29	61	16	37	29	97	81	79	90	40	67		48	88	35	26	34	91	72	97	38	44	01	16
	0.3	1.6	1.0	1.6	1.1	0.7	0.5		1.0	0.4	0.5	0.9	1.2	1.0	1.1	0.5	0.9	1.0	0.5		0.7	0.5	0.2	0.5	0.5	0.7	0.9	0.4	0.6	0.5	0.6	0.3
LYSOPE P18:1	97	36	20	10	62	38	04		77	35	46	11	98	29	31	91	34	85	58		34	05	44	51	72	37	68	63	80	15	75	41
	0.3	1.1	0.9	1.0	0.7	0.5	0.4		0.9	0.3	0.5	0.6	0.8	1.3	1.1	0.7	0.5	0.8	0.5		0.8	0.3	0.2	0.5	0.8	0.4	0.7	0.3	0.7	0.4	0.4	0.2
LYSOPE P18:0	84	47	79	85	03	73	72		67	31	14	42	67	83	75	08	10	51	18		15	85	08	15	01	91	55	98	63	32	96	37
	0.2	0.2	0.2	0.7	0.2	0.2	0.2		0.2	0.1	0.1	0.4	0.2	0.2	0.5	0.1	0.3	0.2	0.1		0.1	0.1	0.1	0.1	0.1	0.1	0.3	0.1	0.2	0.2	0.2	0.1
LYSOPE 18:1	20	92	17	39	80	60	26		08	74	32	64	33	80	50	64	23	02	81		88	07	05	66	61	38	02	49	62	09	79	75
	0.4	0.3	0.4	0.4	0.2	0.4	0.2		0.2	0.1	0.2	0.3	0.2	0.4	0.4	0.3	0.4	0.2	0.3		0.5	0.1	0.2	0.2	0.2	0.2	0.3	0.1	0.3	0.3	0.2	0.1
LYSOPE 18:0	42	02	47	78	58	16	44		52	65	69	81	73	04	21	38	03	87	13		36	44	55	88	88	24	37	76	24	29	20	81
	0.3	1.5	1.3	1.2	1.0	0.5	0.4		1.1	0.3	0.7	0.6	0.9	1.0	1.7	0.6	0.6	0.8	0.6		0.9	0.2	0.1	0.5	0.7	0.6	0.9	0.5	0.9	0.4	0.4	0.4
LYSOPE P20:4	32	18	37	94	80	58	74		42	06	74	94	88	49	03	76	96	16	95		00	54	74	46	91	66	88	34	43	22	71	15
	0.0	0.2	0.0	0.2	0.4	0.1	0.0		0.3	0.1	0.0	0.0	0.2	0.1	0.2	0.0	0.1	0.3	0.0		0.0	0.1	0.0	0.0	0.1	0.0	0.1	0.0	0.1	0.1	0.0	0.0
LYSOPE P20:1	47	74	84	72	13	87	54		25	09	62	96	85	33	66	67	67	39	86		54	40	30	96	21	72	56	73	44	15	93	30
	0.0	0.2	0.0	0.2	0.4	0.1	0.0		0.3	0.1	0.0	0.0	0.2	0.1	0.2	0.0	0.1	0.3	0.0		0.0	0.1	0.0	0.0	0.1	0.0	0.1	0.0	0.1	0.1	0.0	0.0
LYSOPE 19:2	47	71	83	69	08	85	53		21	08	62	95	81	32	63	67	65	35	85		54	39	30	95	19	71	55	72	43	14	92	30
	0.2	0.2	0.3	0.4	0.2	0.1	0.1		0.2	0.1	0.2	0.1	0.1	0.3	0.5	0.1	0.2	0.1	0.2		0.3	0.1	0.1	0.1	0.1	0.1	0.2	0.1	0.3	0.1	0.1	0.1
LYSOPE 20:4	35	00	72	52	80	60	98		16	37	05	91	91	00	29	84	78	92	27		61	63	46	76	42	60	47	90	64	68	88	65

LYSOPE 20:0	0.486	1.963	1.159	1.968	1.578	0.875	0.623	1.121	0.405	1.207	0.787	1.464	1.284	1.898	0.884	1.069	1.142	0.805	1.166	0.625	0.283	0.858	1.038	0.748	1.382	0.679	0.881	0.433	0.893	0.572
LYSOPE P22:6	0.497	2.005	1.185	2.005	1.611	0.894	0.635	1.145	0.414	1.232	0.804	1.496	1.310	1.938	0.901	1.091	1.165	0.822	1.195	0.638	0.289	0.875	1.059	0.764	1.410	0.692	0.899	0.441	0.911	0.584
LYSOPE P22:4	0.069	0.386	0.363	0.584	0.587	0.202	0.114	0.374	0.125	0.212	0.139	0.269	0.320	0.481	0.205	0.173	0.309	0.201	0.135	0.240	0.046	0.150	0.194	0.221	0.257	0.129	0.304	0.115	0.142	0.086
LYSOPE 22:6	0.373	0.314	0.335	0.606	0.279	0.411	0.218	0.179	0.149	0.318	0.291	0.277	0.462	0.782	0.237	0.432	0.270	0.319	0.625	0.188	0.200	0.273	0.309	0.152	0.379	0.211	0.205	0.230	0.301	0.241
LYSOPE 22:4	0.065	0.065	0.066	0.083	0.095	0.061	0.007	0.034	0.033	0.030	0.029	0.105	0.133	0.186	0.051	0.122	0.044	0.060	0.065	0.073	0.055	0.017	0.055	0.017	0.024	0.010	0.095	0.052	0.000	0.030

Supplemental Table 7: Absolute concentrations of lipid species. Quantification of lipid species (nmol / g protein) from freeze-clamped, forebrain regions of male and female, tau-transgenic and non-transgenic mice consuming 10% BD or normal drinking water for 20 weeks. M = male, F = female, WT = non-transgenic mice, TG = tau-transgenic mice, W = water, BD = 1,3-butanediol, PC = phosphatidylcholine, PE = phosphatidylethanolamine, PI = phosphatidylinositol, PS = phosphatidylserine, SM = sphingomyelin, CBS = cerebroside, FFA = free fatty-acid, LPC = lysoPC, LPE = lysoPE, Car = acylcarnitine
